# A cohort-based multi-omics identifies nuclear translocation of eIF5B /PD-L1/CD44 complex as the target to overcome Osimertinib resistance of ARID1A-deficient lung adenocarcinoma

**DOI:** 10.1186/s40164-024-00594-4

**Published:** 2025-01-07

**Authors:** Dantong Sun, Helei Hou, Feiyue Feng, Weizheng Wu, Jingyu Tan, Tongji Xie, Jiayu Liu, Jinsong Wang, Haili Qian, Junling Li, Puyuan Xing

**Affiliations:** 1 Department of Medical Oncology, National Cancer Center/National Clinical Research Center for Cancer/Cancer Hospital, Chinese Academy of Medical Sciences and Peking Union Medical College, Beijing, 100021 China; 2 State Key Laboratory of Molecular Oncology, National Cancer Center/National Clinical Research Center for Cancer/Cancer Hospital, Chinese Academy of Medical Sciences and Peking Union Medical College, Beijing, 100021 China; 3 Department of Medical Oncology, Peking University First Hospital, Beijing, 100034 China; 4 Department of Oncology, The Affiliated Hospital of Qingdao University, No. 7 Jiaxing Road, Qingdao, 266000 Shandong China; 5 Department of Thoracic Surgery, National Cancer Center/National Clinical Research Center for Cancer/Cancer Hospital, Chinese Academy of Medical Sciences and Peking Union Medical College, Beijing, 100021 China; 6 Department of General Surgery, Affiliated Hospital of Zunyi Medical University, Zunyi, 563000 Guizhou China; 7 Medical Research Center, Beijing Chao-Yang Hospital, Capital Medical University, Beijing, 100020 China; 8 Department of Pathology, Peking University People’s Hospital, Beijing, 100044 China

**Keywords:** ARID1A, Nuclear eIF5B/PD-L1/CD44 complex, Ras pathway, Osimertinib, Lung adenocarcinoma

## Abstract

**Background:**

Osimertinib has emerged as a critical element in the treatment landscape following recent clinical trials. Further investigation into the mechanisms driving resistance to Osimertinib is necessary to address the restricted treatment options and survival advantages that are compromised by resistance in patients with EGFR-mutated lung adenocarcinoma (LUAD).

**Methods:**

Spatial transcriptomic and proteomic analyses were utilized to investigate the mechanisms of Osimertinib resistance. Co-IP, MS, RNA-seq, ChIP-seq, RIP-seq, and ATAC-seq were performed in cell lines to further explore the mechanism. To validate the findings, in vitro and in vivo molecular experiments were conducted.

**Results:**

We found that the ARID1A deficiency results in resistance to Osimertinib by hindering programmed cell death through the EZH2/PTEN/E2F1 axis. This altered axis influences PD-L1 transcription through E2F1-mediated promoter activation and PD-L1 translation via the MDM2/eIF5B/PD-L1 axis. Subsequently, ARID1A deficiency results in increased expression of eIF5B and Importin-β1, promoting PD-L1 nuclear-translocation. The nuclear PD-L1 (nPD-L1) interacts with CD44, leading to nPD-L1 complex formation, activation of the RASGEF1A promoter, initiation of the Ras pathway, and contributing to Osimertinib resistance. Targeting the transcription, translation and nuclear-translocation of PD-L1 using lipid nanoparticles (LNPs) overcomes ARID1A deficiency-induced resistance.

**Conclusion:**

ARID1A deficiency promotes PD-L1 nuclear translocation and induces Osimertinib resistance.

**Supplementary Information:**

The online version contains supplementary material available at 10.1186/s40164-024-00594-4.

## Background

Approximately 40% of Asian lung adenocarcinoma (LUAD) patients have driver gene mutations and could benefit from targeted therapies, such as tyrosine kinase inhibitors (TKIs) targeting sensitizing mutations in the epidermal growth factor receptor (EGFR) [1, 2]. Although LUAD patients with sensitizing EGFR mutations might obtain favorable outcomes with EGFR-TKI therapy, a significant proportion eventually develop drug resistance and need additional treatment measures. Given the development of resistance to first-generation EGFR-TKI, Osimertinib, the third-generation EGFR-TKI, has emerged as a solution to this issue, and is now widely utilized as the first-line treatment strategy. However, the development of resistance to Osimertinib has significantly limited the efficacy of EGFR-TKI treatment. It is established that acquired molecular alterations, such as EGFR C797X mutations, MET or HER2 amplification, and other mechanisms like histologic transformation, have contributed to this resistance. However, a substantial proportion of patients (40%-50% in first-line treatment, and 30%-40% in second-line treatment) develop resistance to Osimertinib due to unknown mechanisms [3]. Therefore, further exploration of the mechanisms of resistance to Osimertinib is necessary in order to establish effective treatment strategies for these patients. The most recent study indicated that the genomic signature of the switch/sucrose nonfermenting (SWI/SNF) chromatin remodeling complex may support resistance to EGFR-TKI [4]. This finding caught our attention and led to the execution of this study. The SWI/SNF chromatin remodeling complex, especially the AT-rich interaction domain 1A (ARID1A) subunit, plays a crucial role in maintaining optimal cellular function. In addition, ARID1A interacts with enhancer of zeste 2 polycomb repressive complex 2 subunit (EZH2) and thus influences the expression of a range of tumor-related molecules. It has been suggested that EZH2 inhibitors may prove effective in combatting cancer through the mechanism of synthetic lethality in tumors with ARID1A mutations [5, 6]. Our prior research has confirmed that ARID1A functions as the tumor suppressor of LUAD, and the absence of ARID1A activity has contributed to metastasis and resistance to first generation EGFR-TKI [7–9]. It is important to conduct additional investigations to examine the relationship between ARID1A expression and the response to third-generation (3G) EGFR-TKIs, such as Osimertinib. Additionally, the mechanistic factors associated with ARID1A deficiency are currently not well understood and require further exploration.

Programmed cell death ligand 1 (PD-L1) is widely recognized for its role in facilitating immune evasion of tumor cells by interacting with its receptor, programmed cell death-1 (PD-1). Typically, PD-L1 is localized primarily in the cell membrane and cytoplasm, although it is also present in the nucleus to a lesser extent. Positive expression of nuclear PD-L1 (nPD-L1) has been observed in various types of cancer, particularly lung cancer [10]. Studies have shown that nPD-L1 plays a role in regulating cell proliferation and is associated with an unfavorable prognosis in non-small cell lung cancer (NSCLC) [11]. In our current research, we have observed that a lack of ARID1A leads to changes in the EZH2/PTEN/E2F1 axis and the downstream MDM2/eIF5B/PD-L1 axis, which play a role in the transcription of PD-L1 via E2F1-mediated activation of the PD-L1 promoter, as well as in the translation of PD-L1. ARID1A deficiency also results in increased expression of Importin-β1 and eIF5B, which help in the translocation of PD-L1 into the nucleus. Additionally, we found that CD44 is translocated along with PD-L1 and assists in the formation of the nuclear PD-L1 complex, functioning as a transcription factor. The nPD-L1 complex then activates the promoter of RasGEF domain family member 1A (RASGEF1A) through the transcription factor function of CD44. This activation initiates signaling through the downstream Ras pathway [12], ultimately leading to resistance to Osimertinib in EGFR-mutant LUAD. Fortunately, targeting either the EZH2/PTEN/E2F1 axis or nPD-L1 could overcome resistance induced by ARID1A deficiency, an approach with potential utility as a supportive treatment in combination with Osimertinib in patients with EGFR-mutant LUAD.

## Materials and methods

### Patient recruitment and prognostic evaluation

In this study, we recruited a total of 101 patients with EGFR-mutant LUAD who received treatment with any type of EGFR-TKI, including 77 patients treated with Osimertinib (Table S1). We conducted immunohistochemical (IHC) staining on samples from 77 patients treated with Osimertinib to evaluate biomarkers and assess prognosis. The expression levels of biomarkers included ARID1A, MDM2, and PD-L1 were evaluated using IHC staining. Additionally, we selected samples from the cohort of 101 patients with EGFR-mutant LUAD for proteomic analysis. The detailed procedures for IHC staining and proteomic analysis, as well as the ethical approval statement, are provided in the supplementary materials and methods. The study was approved by the Ethics Committee of Cancer Hospital Chinese Academy of Medical Sciences (No. NCC-007421).

### Culture and stable transduction of cell lines

We utilized several LUAD cell lines in this study, specifically A549, NCI-H1299, NCI-H1975, HCC4006, HCC2279, and NCI-H1563. These cell lines were cultured in RPMI-1640 medium. Additionally, we employed the gastric cancer cell line AGS, which was cultured in F12K medium. The tool cell line HEK293T was also utilized and cultured in DMEM. Supplementary file 1 provides all the necessary information regarding the short tandem repeat (STR) Cell ID assays. For the construction of stably transfected cell lines, we employed lentiviruses containing short hairpin RNAs (shRNAs) for ARID1A, MDM2 and PD-L1, a single-guide RNA (sgRNA) for eIF5B and ARID1A, and overexpression plasmids for PD-L1 and ARID1A. Notably, we utilized the Tet-on system to construct the PTEN overexpression cell line, in which PTEN expression could be induced by doxycycline. For more detailed information, please refer to Table S2 and the supplementary materials and methods. All experiments were conducted according to the rules of the Declaration of Helsinki and the guidelines of the National Health and Family Planning Commission of the Professional Regulation Commission (PRC).

### Multiomics analysis

We employed a variety of omics techniques to investigate the mechanisms of resistance to EGFR-TKIs and to understand how ARID1A contributes to the development of resistance to Osimertinib. At the cellular level, we utilized RNA sequencing (RNA-seq), mass spectrometry (MS), chromatin immunoprecipitation sequencing (ChIP-seq), assay for transposase-accessible chromatin using sequencing (ATAC-seq), and RNA immunoprecipitation and sequencing (RIP & RIP-seq) to gather supporting evidence for our subsequent experiments. The supplementary materials and methods section provides a detailed explanation of the methodologies employed for multiomics analysis.

### Spatial transcriptomics and proteomics

In this study, we utilized spatial omics, such as transcriptomics and proteomics, to investigate and confirm our discoveries in patient tissue samples. The supplementary materials and methods section provides a detailed explanation for spatial transcriptomics and proteomics.

### Cellular phenotype experiments and drug resistance validation

To investigate the effects of gene editing on the cell proliferation, migration, and invasion abilities, we conducted several experiments: the colony formation assay, the 3D cell culture, the wound healing assay, the Transwell cell migration and invasion assays, and real-time dynamic cell imaging with the IncuCyte system. Additionally, we used transmission electron microscopy (TEM) to observe intracellular autophagosomes and autolysosomes. MTS assays were also performed to determine the half-maximal inhibitory concentration (IC50) of Osimertinib in different cell lines, as well as the cell viability evaluation after the treatment of different drug formulations. All the specific steps of the experiments are outlined in the supplementary materials and methods section.

### Verification of the cellular mechanism

To validate the findings of our multiomics analysis in this study, we employed various techniques. Western blot (WB) analysis and quantitative PCR (qPCR) were used to confirm gene expression. The luciferase reporter assay was utilized to investigate transcription factor binding sites and the impact of transcription factors on protein expression. Coimmunoprecipitation (Co-IP) was performed to analyze binding interactions between proteins. Immunofluorescence (IF) staining and confocal microscopy were conducted to visualize the localization and staining intensity of proteins. Detailed experimental procedures are provided in the supplementary materials and methods section.

### In vivo xenograft animal model verification

We established a xenograft model using BALB/c nude mice to validate the findings in our study. The mice were treated with various regimens by either injection or oral administration. We calculated the tumor volume to assess the effectiveness of the treatments. The selection of pharmaceuticals is based on prior and current mechanistic studies, which target the relevant pathways of this research to achieve the intended objectives. The animal experiments were carried out by strictly following the guidelines of the Committee on the Ethics of Animal Experiments of Cancer Hospital Chinese Academy of Medical Sciences (No. NCC2023A316). Additional information regarding the in vivo experiment can be located in the supplementary materials and methods section.

### Database analysis and statistical analysis

Multiple online databases, including cBioPortal for Cancer Genomics (cBioPortal), Investigating Genetic Model of Drug Response (iGMDR), The Cancer Genome Atlas (TCGA), Gene Expression Profiling Interactive Analysis (GEPIA), Tumor Immune Estimation Resource 2.0 (TIMER 2.0), and NucleOlar localization sequence Detector (NoD), were utilized for the investigation and validation of the findings. Additional information regarding the database analysis can be found in the supplementary materials and methods section. Nonparametric tests were employed and P values were determined using two-tailed tests. Statistical significance was defined as P < 0.05 (*), P < 0.01 (**), P < 0.001 (***), or P < 0.0001 (****).

## Results

### ARID1A deficiency was strongly correlated with the Osimertinib resistance in EGFR-mutant LUAD patients

**Fig. 1:**
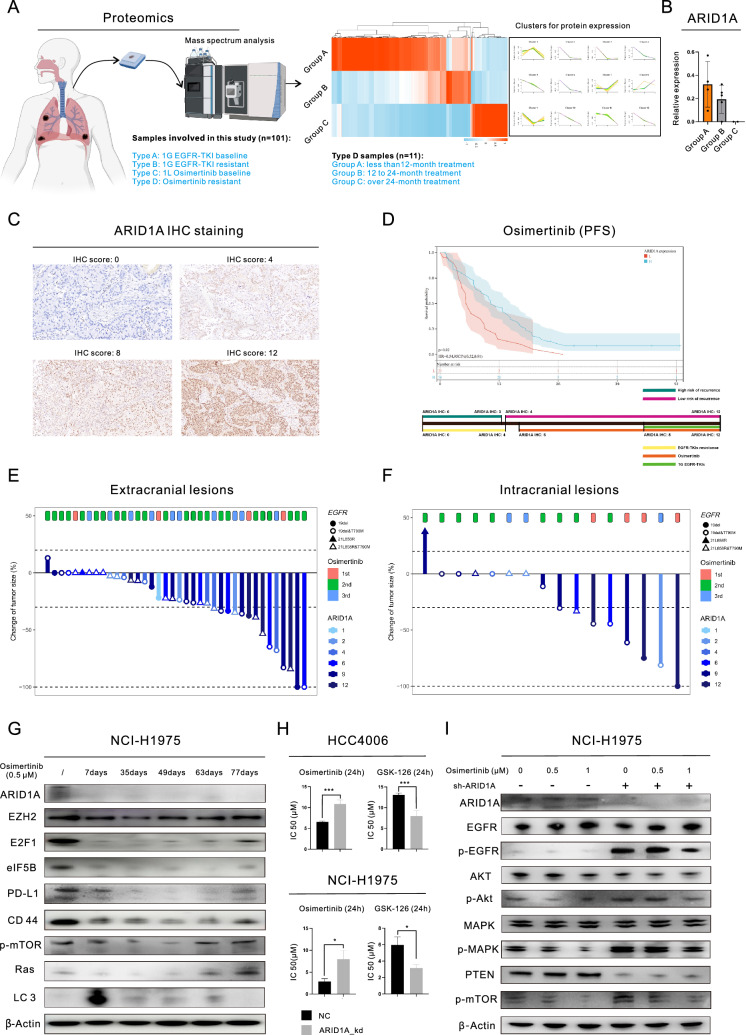
ARID1A deficiency is associated with resistance to Osimertinib in lung adenocarcinoma (LUAD). **A** Proteomic analysis based on 101 samples collected from patients after Osimertinib treatment; **B** Changes in ARID1A expression in post-Osimertinib treatments samples, based on proteomic analysis; **C** Images of IHC staining for ARID1A in LUAD tissues; **D** Progression-free survival curve of Osimertinib-treated patients with LUAD stratified by ARID1A expression (top) and the schematic diagram showing the correlation between ARID1A expression and the risk for disease relapse and EGFR-TKI resistance (bottom); **E**–**F** Waterfall plots showing the response to Osimertinib treatment in LUAD patients based on ARID1A expression; **G** WB analysis demonstrated the changes in the expression of proteins following treatment with 0.5 µM Osimertinib in LUAD cells; **H** MTS assays revealed that drug sensitivity was observed following ARID1A knockdown in LUAD cell lines, with the cells being treated with Osimertinib or GSK-126 for a duration of 24 h; **I** WB analysis demonstrated the changes in the expression of proteins after ARID1A knockdown and/or Osimertinib treatment (0.5 µM or 1 µM, 24 h) in LUAD cells

In total, 101 patients (4 types of samples) who received EGFR-TKI treatment were enrolled in this study (Fig. 1A, left) for the proteomic analysis. We obtained 11 samples from patients who had undergone Osimertinib treatment and analyzed the proteins in these samples using a proteomics approach (Fig. 1A, right). We grouped the samples based on the duration of Osimertinib treatment and examined the changes in protein expression. Our findings showed that different patterns of change across the proteins, leading to different cluster classifications. Specifically, we observed a consistent decrease in the expression of ARID1A after Osimertinib treatment (Fig. 1B). In this study, we investigated the frequency of mutations in components of the SWI/SNF complex in pancancer datasets. Figure S1A shows that ARID1A had the highest mutation frequency (9%), followed by SMARCA4 (4%) and ARID1B (3%). To identify potential drugs related to ARID1A, we searched the iGMDR database (Figure S1B) and found a likely association between ARID1A and drugs targeting EGFR. ARID1A may influence functions and pathways downstream of EGFR, including kinase activity, the PI3K/mTOR and MAPK pathways. The impacts of ARID1A mutations on protein expression were investigated, as shown in Figure S1C. ARID1A mutations (detected in the HCC2279 and NCI-H1563 cell lines) led to a significant decrease in ARID1A expression. Therefore, ARID1A protein expression was chosen as the biomarker for further experiments, as it offers greater convenience in clinical settings.

We recruited 77 individuals with EGFR-mutant LUAD who received Osimertinib treatment to examine the connection between baseline ARID1A expression and the outcome of Osimertinib therapy. Table S1 provides details about these patients. In Fig. 1C, we show IHC staining for ARID1A. Patients with low ARID1A expression had a significantly shorter median progression-free survival (PFS) time after Osimertinib treatment than those with high ARID1A expression (Fig. 1D, 6.20 months versus 11.20 months, P = 0.0183). Building upon our previous studies [7, 8], we proposed a model to predict the risk of metastasis and resistance to EGFR-TKIs in LUAD patients based on ARID1A expression. This model is shown in the bottom panel of Fig. 1D. Computed tomography (CT) and Magnetic resonance imaging (MRI) were performed to examine the effect of Osimertinib in patients (Fig. 1E and F). The findings revealed that patients with high ARID1A expression (indicated by a darker color) had a positive response to the treatment. In this group, there was a higher incidence of complete response (CR) or partial response (PR) in both extracranial (39.3% compared to 22.2%) and intracranial (54.6% compared to 25.0%) lesions, as shown in Figure S1D.

**Fig. 2:**
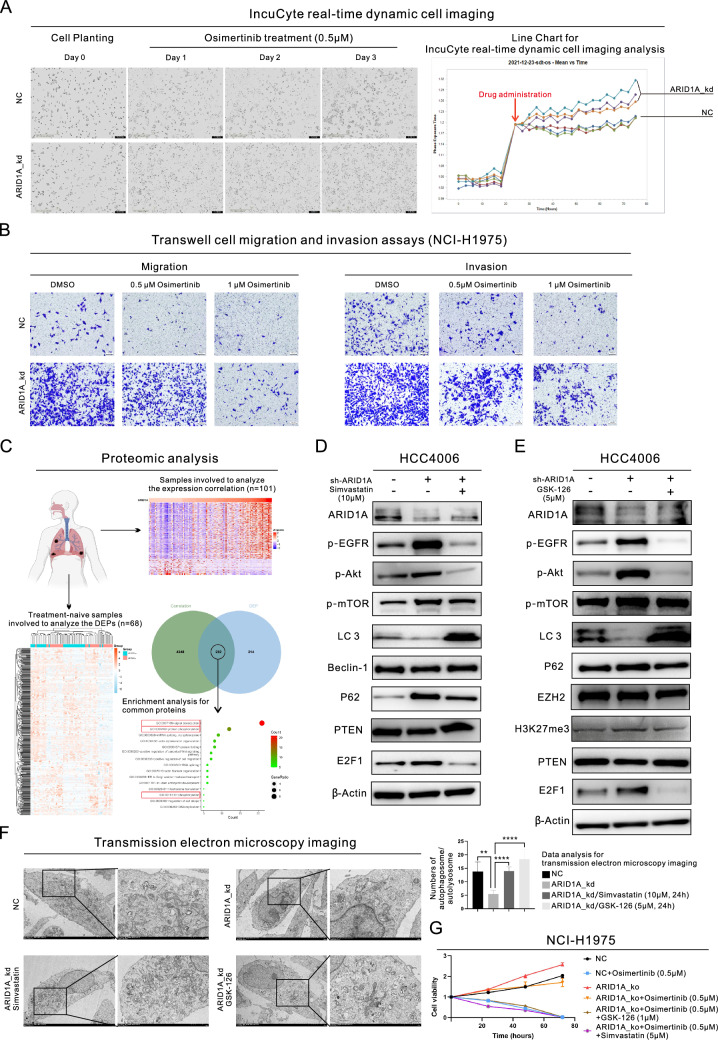
ARID1A deficiency alters EZH2 and E2F1 activity, inhibits autophagy and promotes tumor progression and Osimertinib resistance in lung adenocarcinoma (LUAD). **A** Representative images and growth curve of HCC4006 cells with or without ARID1A knockdown and exposed to 0.5 µM Osimertinib, generated by real-time dynamic cell imaging with the IncuCyte system; **B** Transwell migration and invasion assays in NCI-H1975 cells with or without ARID1A knockdown and exposed to 0.5 µM or 1 µM Osimertinib; **C** Proteomic analysis based on clinical samples revealed the proteins related to ARID1A and the subsequent enrichment analysis; **D**-**E** Western blot analysis demonstrated the changes in the expression of proteins in LUAD cells after ARID1A knockdown and treatment with the indicated drugs (Simvastatin and GSK-126); **F** Representative transmission electron micrographs of HCC4006 cells with or without ARID1A knockdown and with or without treatment with GSK-126 (5 µM for 24 h) or Simvastatin (10 µM for 24 h) showing the changes in the numbers of autophagosomes or autolysosomes; **G** The combination treatment (Osimertinib + GSK126 or Osimertinib + Simvastatin) successfully overcame the resistance to Osimertinib induced by ARID1A knockout NCI-H1975 cells

We then conducted the experimental validation through WB analysis (Fig. 1G). The findings demonstrated that the expression of ARID1A significantly decreased right after administration of 0.5 µM Osimertinib, and this reduced expression level persisted even after the onset of acquired resistance, as evidenced by the upregulation of resistance-associated proteins (We found that short-term exposure to Osimertinib resulted in the impairment of cellular viability decreased expression of resistance-associated proteins, such as EZH2 and E2F1, leading to inhibition of EGFR downstream pathways. However, with prolonged exposure, the expression of these resistance-associated proteins was gradually restored and EGFR downstream pathways were reactivated in these cells). Based on the evidence presented above, ARID1A deficiency may play a considerable role in the development of Osimertinib resistance. To confirm this hypothesis, we conducted subsequent experiments using ARID1A knockdown (ARID1A_kd) cell lines. ARID1A_kd led to a significant increase in the IC50 of Osimertinib in LUAD cell lines (HCC4006: 6.53 µM versus 10.87 µM, P = 0.0001; NCI-H1975: 2.88 µM versus 7.94 µM, P = 0.0153), as demonstrated in Fig. 1H. Furthermore, we investigated the downstream pathways of EGFR signaling (Figure S1E) and observed an increase in the level of p-EGFR, as well as the levels of the downstream proteins p-Akt, p-mTOR, and p-STAT3. Even after treatment with 0.5 µM or 1 µM Osimertinib in NCI-H1975 cells (Fig. 1I), the p-EGFR and downstream pathways remained relatively active in ARID1A_kd cells compared to the negative control (NC) cells. Figure S1F illustrated the levels of expression of total proteins. Then, we conducted cellular phenotype assays to further confirm the impact of ARID1A_kd. Our results showed a significant increase in the colony-forming ability after ARID1A_kd (Figure S1G), as well as an increase in tumorigenesis (Figure S1H). As shown in Figure S1H, we observed the presence of tumor spheres in the ARID1A_kd group after only 2 days, whereas no tumor spheres were observed in the NC group until the end of the observation period (4 days). Moreover, ARID1A_kd significantly increased the proliferation of HCC4006 cells treated with 0.5 µM Osimertinib, as observed through time-lapse imaging using the IncuCyte system (Fig. 2A). Furthermore, the Transwell assays (Fig. 2B) demonstrated that ARID1A_kd NCI-H1975 cells exhibited increased migration and invasion abilities.

### ARID1A deficiency hinders autophagy by activating the EZH2/PTEN/E2F1 axis

To investigate the mechanism by which ARID1A deficiency leads to resistance to Osimertinib, we conducted a comprehensive analysis using multiple techniques. Our RNA-seq analysis (Figure S1I) revealed the potential impact of ARID1A deficiency on programmed cell death, as well as on the increased transcription of E2F1 and the impaired transcription of PTEN (our previous study [8] showed that the E2F pathway is activated in cells with ARID1A deficiency). Additionally, we evaluated correlations of ARID1A expression using proteomic analysis in a total of 101 samples, among which 68 samples from treatment-naive individuals were used to examine the differentially expressed proteins (DEPs) based on the level of ARID1A expression (Fig. 2C). Our proteomic analysis revealed strong associations between ARID1A and signal transduction and protein phosphorylation. To further investigate these associations, we conducted MS analysis to identify phosphorylated proteins in LUAD cell lines, as shown in Figure S1J. Despite its effects on the cell cycle and downstream pathways of EGFR, such as MAPK and mTOR signaling, ARID1A_kd may contribute to regulating various types of programmed cell death, including autophagy and apoptosis. To explore this possibility further, we conducted additional experiments both in vivo and in vitro.

**Fig. 3:**
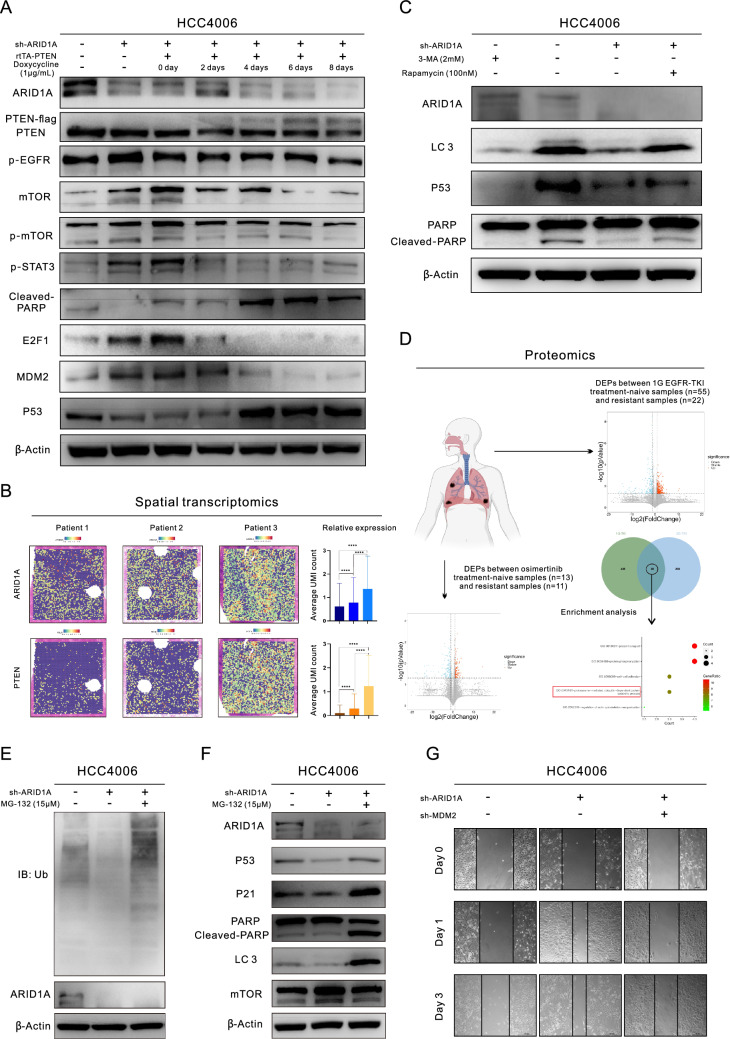
ARID1A deficiency influences the EZH2/PTEN/E2F1 axis, which targets MDM2 and induces resistance to Osimertinib. **A** Western blot (WB) analysis demonstrated the changes in the expression of proteins following PTEN overexpression; **B** Spatial transcriptomic analysis revealed the positive correlation between ARID1A and PTEN transcription; **C** WB analysis of target protein expression in HCC4006 cells with or without ARID1A knockdown and exposure to 3-MA (2 mM, 24 h) or Rapamycin (100 nM, 24 h) treatment; **D** Proteomic analysis demonstrated the common mechanism of EGFR-TKI resistance; **E**–**F** WB analysis demonstrated the changes in the expression of proteins in LUAD cells with ARID1A knockdown, or treatment with MG-132 (15 µM, 24 h); **G** Wound healing assays in control HCC4006 cells, HCC4006 cells with ARID1A knockdown and HCC4006 cells with combined ARID1A knockdown/MDM2 knockdown

We discovered that ARID1A_kd significantly increased the activity of the Akt/mTOR pathway (Fig. 1I, Figure S1E, Figure S1K, and Fig. 2D-E), which is crucial for initiating autophagy. Our WB analysis also showed that ARID1A_kd suppressed autophagy, as seen by the changes in the expression of autophagy biomarkers (Fig. 2D-E and Figure S1K). To further validate this relationship, we performed TEM analysis, as shown in Fig. 2F, and observed that ARID1A_kd led to significant decreases in the numbers of autophagosomes/autolysosomes in LUAD cells. In further validating the mechanisms involved, we discovered that ARID1A_kd resulted in an increased level of E2F1 but a decreased level of PTEN. We also observed that inhibiting E2F1 with Simvastatin reversed the inhibition of PTEN expression and autophagy induced by ARID1A_kd (Fig. 2D). Additionally, EZH2 acts as a counteracting factor for ARID1A and can be effectively inhibited by ARID1A activity [2, 7]. Therefore, we treated ARID1A_kd cells with GSK-126 to inhibit the function of EZH2. Inhibiting EZH2 also reversed the effects of ARID1A_kd on the expression of downstream proteins (Fig. 2E). Additionally, our TEM analysis results confirmed the reversal of autophagy in ARID1A_kd cells following treatment with Simvastatin and GSK-126, as demonstrated in Fig. 2F. In light of the aforementioned results, we conducted a combination therapy on the ARID1A_knockout (ARID1A_ko) cells, as illustrated in Fig. 2G and the combination treatments successfully overcame the resistance to Osimertinib induced by ARID1A loss. Additionally, through the gradual increase in PTEN expression conferred by the Tet-on system, we observed gradual inhibition of E2F1 and the downstream pathways of EGFR. This inhibition may counteract the effects of ARID1A_kd (Fig. 3A and Figure S2A). Furthermore, spatial transcriptomic analysis of clinical samples confirmed a positive correlation between ARID1A and PTEN expression, further supporting our findings (Fig. 3B). The relationships between these molecules and autophagy are shown in Figure S2B.

### EZH2/PTEN/E2F1 axis-inhibited autophagy attenuates MDM2/P53-primed apoptosis

To further validate the impact of ARID1A_kd on apoptosis, we conducted additional experiments. Initially, we observed a positive association between the two types of programmed cell death (autophagy and apoptosis) in LUAD samples (Figure S2C). Additionally, modulating the level of autophagy using 3-Methyladenine (3-MA) or Rapamycin resulted in a corresponding change in the level of apoptosis, as indicated by the abundance of cleaved PARP (Fig. 3C). The findings from our RNA-seq analysis (Figure S2D) further supported the positive correlation between autophagy and apoptosis in LUAD cells, as evidenced by the expression of various programmed cell death biomarkers. Furthermore, the RNA-seq data demonstrated a notable decrease in the transcription of P53, a crucial regulator of apoptosis, in ARID1A_kd cells (Figure S2D). We further confirmed this finding through WB analysis of cell lines (Figure S1E). Additionally, modulation of autophagy also influenced the expression of P53, which could serve as the link between autophagy and apoptosis (Fig. 3C). However, the specific mechanism through which ARID1A_kd influences P53 expression is still unclear, and we tackled this question using proteomics alongside additional experimental validation. Figure 3D shows that the proteasome-mediated ubiquitin (Ub)-dependent catabolic process was active in tissues with resistance to both 1G and 3G EGFR-TKIs. Via WB analysis (Fig. 3E), we observed that ARID1A_kd effectively enhanced proteasome activity, as evidenced by the decrease in the Ub level. Conversely, inhibiting the proteasome using MG-132 enhanced autophagy, increased the expression of P53 and P21, and promoted apoptosis (Fig. 3F). Additionally, our findings indicated that inhibiting EZH2 (using GSK-126) or E2F1 (using Simvastatin) also suppressed proteasome activity (Figure S2E). This suggests that proteasome activity and protein degradation, particularly those processes involving P53, may be influenced by ARID1A_kd, leading to resistance to Osimertinib.

P53 can be degraded via the proteasome, an event that is linked to Ub, and MDM2 is considered the most important E3 ubiquitin ligase for P53 [13]. Herein, WB analysis revealed that ARID1A_kd significantly increased the expression of MDM2 (Figure S1E and Figure S2F). Additionally, we found that the expression of MDM2 is also regulated by the EZH2/PTEN/E2F1 axis (Fig. 3A, Figure S2A and Figure S2E) in LUAD cells with or without ARID1A_kd, which ultimately affects the expression of P53. Based on our findings, MDM2 is likely to be a target downstream of ARID1A_kd and may related to Osimertinib resistance. To validate these results, we evaluated MDM2 expression using IHC staining of samples from 77 patients (Figure S2G). High expression of MDM2 was associated with poor PFS in patients undergoing Osimertinib treatment (6.70 months versus 13.77 months, P = 0.0402, Figure S2H). We proceeded to perform MDM2 knockdown (MDM2_kd) in ARID1A_kd LUAD cells (Figure S2F) and discovered that MDM2_kd greatly impaired the migration of these cells, which was preliminary enhanced by ARID1A_kd (Fig. 3G and Figure S2I), and restored Osimertinib resistance in ARID1A_kd cells (10.8 µM compared to 7.50 µM, P = 0.0005, Figure S2J). The schematic diagram for this section is presented in Figure S2K.

### 3.4 EZH2/PTEN/E2F1 axis increases PD-L1 transcription and eIF5B-facilitated PD-L1 translation in LUAD cells with ARID1A deficiency

**Fig. 4:**
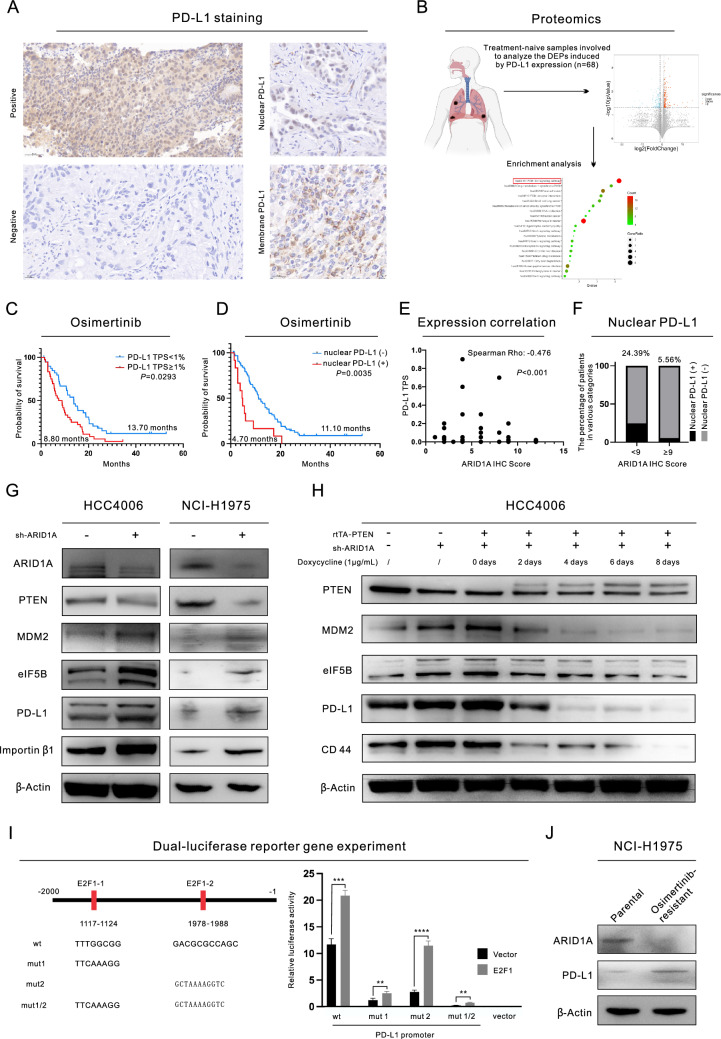
ARID1A deficiency upregulates PD-L1 expression through the EZH2/PTEN/E2F1 axis and the downstream protein MDM2. **A** Images of IHC staining for PD-L1 in lung adenocarcinoma (LUAD) tissues; **B** Proteomic analysis results showing the DEPs influenced by PD-L1 expression and the subsequent enrichment analysis; **C** Prognostic value of the PD-L1 TPS for PFS in patients with Osimertinib treatment; **D** Prognostic value of nuclear PD-L1 status for PFS in patients with Osimertinib treatment; **E** Correlation between ARID1A and PD-L1 expression in LUAD tissues collected in our center; **F** Nuclear PD-L1-positive percentages of LUAD patients with different expression levels of ARID1A; **G** Western blot (WB) analysis demonstrated the changes in the expression of proteins in LUAD cells with ARID1A knockdown; H. WB analysis demonstrated the changes in expression in LUAD cells with PTEN overexpression; **I** The dual luciferase reporter assay suggested that E2F1 could serve as an important transcription factor for PD-L1;** J** WB analysis demonstrated the changes in the expression of ARID1A and PD-L1 in Osimertinib-resistant cells

The expression level of PD-L1 in tissue samples from 77 participating patients who underwent EGFR-TKI treatment was also assessed. Images of IHC staining for PD-L1 are presented in Fig. 4A, revealing that PD-L1 was detected not only in the plasma membrane but also in the nucleus. Additionally, we included 68 treatment-naive samples in our analysis to investigate the pathways influenced by PD-L1 expression (Fig. 4B). Our findings indicated that alterations in PD-L1 expression may impact various cancer-related pathways, such as the PI3K/Akt pathway, and affect sensitivity to anticancer treatments. We utilized the tumor proportion score (TPS) to assess global PD-L1 expression in the samples. Our results showed that a higher TPS was associated with a shorter PFS time after Osimertinib treatment (8.80 months versus 13.70 months, P = 0.0293, Fig. 4C). Interestingly, while earlier research has indicated a relationship between elevated PD-L1 expression and the activation of PI3K signaling pathways as well as resistance to EGFR-TKI treatment [14], the underlying mechanisms remain ambiguous. Presently, these findings imply that nPD-L1 may serve as a more reliable biomarker than global PD-L1 for forecasting the efficacy of Osimertinib therapy, potentially acting as a pivotal initiator for subsequent pathway activation and the development of resistance. The group of patients who tested positive for nPD-L1 had a shorter PFS time than the group of patients who tested negative (4.70 months versus 11.10 months, P = 0.0035, Fig. 4D). However, no prognostic effect of PD-L1 was found in the treatment of first generation EGFR-TKI (Figure S3A and S3B). Furthermore, ARID1A deficiency was associated with a higher level of PD-L1 expression. This pattern was evident in both the TISIDB (Figure S3C) and the clinical samples in the current study (Fig. 4E). Additionally, the group with an ARID1A IHC score of < 9 had a higher rate of nPD-L1 positivity (24.39% versus 5.56%, Fig. 4F). Then, we proceeded with additional experiments to validate our discoveries. ARID1A_kd led to a significant increase in the expression of PD-L1 (Fig. 4G and Figure S3D). IF staining also revealed that ARID1A_kd increased both the expression and nuclear localization of PD-L1 (Figure S3E). Similarly, the expression of PD-L1 was also regulated by the EZH2/PTEN/E2F1 axis. Specifically, gradual overexpression of PTEN resulted in a gradual decrease in PD-L1 expression (Fig. 4H and Figure S3F). Furthermore, data in both the GEPIA database and TISIDB indicated a positive correlation between EZH2 and PD-L1 expression (Figure S3G). In addition, inhibiting EZH2 (using GSK-126) or E2F1 (using Simvastatin) effectively inhibited the expression of PD-L1 (Figure S3H and S3I). Moreover, we conducted a dual-luciferase reporter assay (Fig. 4I) to investigate the role of E2F1 as a transcription factor for PD-L1. We discovered that there are two main binding sites, namely, site 1 (1117–1124) and site 2 (1978–1988), at which PD-L1 and E2F1 interact. To further elucidate the correlation between ARID1A and PD-L1 expression, we conducted subsequent WB analyses in Osimertinib-resistant LUAD cells, revealing a loss of ARID1A expression alongside an upregulation of PD-L1 expression (Fig. 4J).

**Fig. 5:**
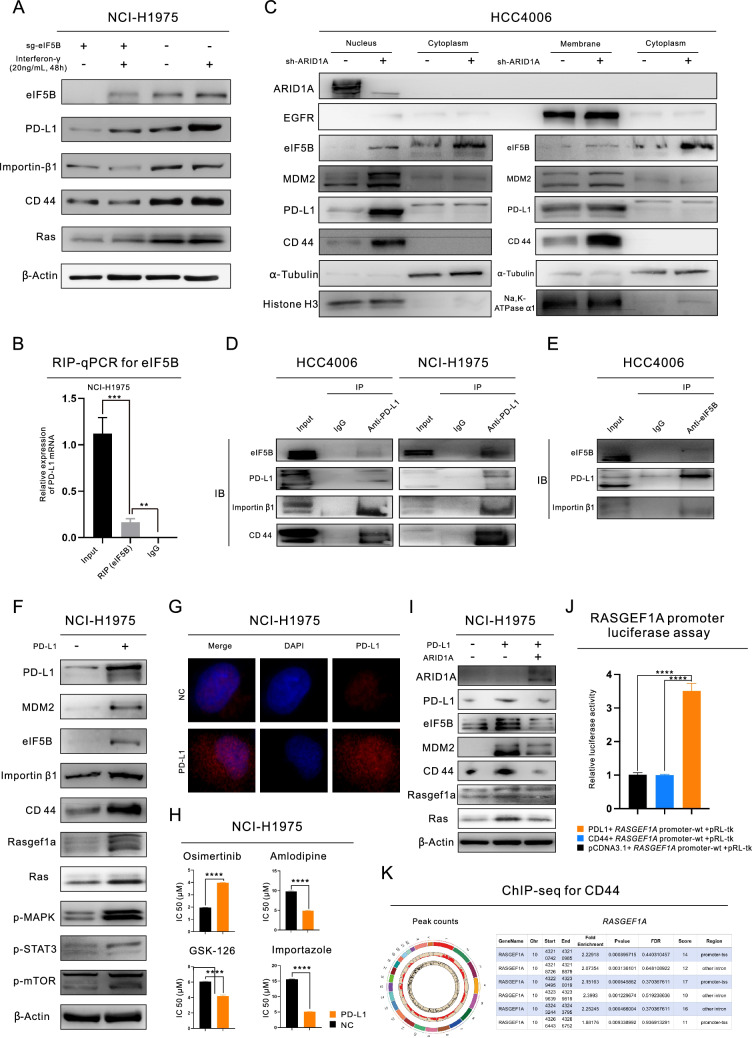
ARID1A deficiency enhances the activity of the MDM2/eIF5B/PD-L1 axis, which triggers the nuclear translocation of PD-L1 and activates Ras signaling via promotion of RASGEF1A promoter activation. **A** eIF5B is essential for PD-L1 expression, and eIF5B knockout (eIF5B ko) decreases the expression of PD-L1 even upon interferon-γ treatment; **B** The specific interaction between eIF5B and PD-L1 was identified through RNA immunoprecipitation and qPCR; **C** Separation of nuclear and membrane proteins in lung adenocarcinoma (LUAD) cells and subsequent WB analysis; **D**-**E** Coimmunoprecipitation (Co-IP) of PD-L1 and eIF5B in LUAD cells; **F** WB analysis demonstrated the changes in the expression of proteins in LUAD cells with PD-L1 overexpression (PD-L1_oe); **G** Immunofluorescence (IF) staining demonstrated the localization of PD-L1 in LUAD cells with or without PD-L1_oe;** H** MTS assays of drug sensitivity in LUAD cells with PD-L1_oe; **I** WB analysis demonstrated the changes in the expression of proteins in PD-L1_oe LUAD cells with or without ARID1A overexpression; **J** The luciferase reporter assay for RASGEF1A promoter; **K** The ChIP-seq analysis of CD44 uncovered the distinct combination of the RASGEF1A promoter

We discovered that MDM2 is an important target downstream of the EZH2/PTEN/E2F1 axis. In subsequent experiments, we found that not only the transcription but also the translation of PD-L1 is controlled by ARID1A_kd in a manner promoted by MDM2 and mediated by eIF5B. Through IP-MS and RNA-seq analyses, we identified four genes that are primarily regulated by MDM2 at both the transcriptional and translational levels (Figure S3J). However, only eIF5B could be confirmed in the GEPIA database. WB analysis showed that the expression of eIF5B is significantly increased by ARID1A_kd and/or MDM2_kd (Fig. 4G, Figure S3D, Figure S3K and Figure S3L). Furthermore, we confirmed that eIF5B is controlled by the EZH2/PTEN/E2F1 axis (Fig. 4H, Figure S3H and Figure S3I). We proceeded to establish eIF5B knockout (eIF5B_ko) cell lines for WB analysis. Our findings demonstrated that eIF5B_ko had a significant impact on reducing the expression of PD-L1 and inhibiting the downstream pathways of EGFR (Figure S3M and Fig. 5A). The presence of eIF5B is necessary for the expression of PD-L1, as shown by the response to interferon-γ treatment. It suggested that the expression level of PD-L1 was not significantly increased in eIF5B_ko cells after interferon-γ treatment (Fig. 5A). Since eIF5B acts as a functional component for initiating protein translation and binds to the ribosome, we conducted anti-eIF5B RIP and qPCR analysis. These experiments revealed that eIF5B directly binds to the mRNA of PD-L1 (Fig. 5B), suggesting that eIF5B facilitates the translation of PD-L1 mRNA on the ribosome. Additionally, RIP-seq analysis revealed that the mRNA bound to eIF5B is functionally related to the PI3K/Akt pathway and EGFR-TKI resistance (Figure S3N).

### eIF5B facilitates PD-L1 nuclear translocation in ARID1A deficient LUAD cells

Given the above observations, we were interested in determining the cellular localization of the upregulated PD-L1 protein in ARID1A_kd cells. To this end, we performed WB analysis on the separated nuclear, cytoplasmic, and membrane protein fractions. Our findings showed that the upregulated PD-L1 protein was localized predominantly in the nucleus (Fig. 5C and Figure S4A). Additionally, nuclear MDM2 and eIF5B were also upregulated in ARID1A_kd cells. PD-L1 is classified as a membrane protein, and the mechanism by which it enters the nucleus is unclear. To investigate this mechanism further, we utilized the NucleOlar localization sequence Detector (NoD) database. Our findings revealed that PD-L1 does not contain any predicted nucleolar localization sequences (NoLSs), suggesting that PD-L1 cannot be transported into the nucleus by importin on its own. Instead, other proteins must bind to PD-L1 and assist in its nuclear translocation. Interestingly, we discovered that the mRNA binding to eIF5B had additional functions related to “protein transport” and “protein import into the nucleus”, as illustrated in Figure S4B. We conducted a search in the NoD database and identified a series of NoLSs in the protein sequence of eIF5B (Figure S4C), prompting us to consider that eIF5B may play a role in facilitating the nuclear localization of PD-L1. To validate this hypothesis, we performed Co-IP and WB analysis, revealing the binding between eIF5B and PD-L1 (Fig. 5D-E and Figure S4D) and the essential role of eIF5B in promoting the nuclear localization of PD-L1 (Figure S4E). A previous study indicated that Importin-β1 is involved in transporting PD-L1 into the nucleus [11]. However, based on our findings (Fig. 5D-E and Figure S4C-S4E), we believe that eIF5B is the protein that connects Importin-β1 and PD-L1, since the protein sequence of PD-L1 does not contain any NoLSs that could be targeted by Importin-β1. ARID1A_kd has been found to play a role in regulating the expression and localization of Importin-β1 in the nucleus (Fig. 4G and Figure S4F). Importantly, Importin-β1 expression seems to be influenced by other factors that are downstream targets of the pathways activated by ARID1A_kd, such as E2F1 inhibition (Simvastatin treatment), MDM2-kd, and eIF5B (Figure S3M and Figure S4G).

### The functional characterization of nPD-L1 and the exploration of downstream targets

ARID1A_kd increased the nuclear localization of PD-L1. However, the specific role of nPD-L1 in ARID1A_kd cells is not yet understood. We conducted IP followed by MS to analyze the binding of PD-L1 in the nucleus and cytoplasm (Figure S4H). The "RNA binding" function was enriched in proteins binding specifically to nPD-L1 (Figure S4I) compared to proteins binding to cytoplasmic PD-L1 (Figure S4J). To further investigate this finding, we performed RIP-seq for PD-L1 and confirmed that PD-L1 indeed has a function in RNA binding. The mRNA binding to PD-L1 was also suggested to be involved in "signal transduction" (Figure S5A). We proceeded to establish a cell line with PD-L1 overexpression (PD-L1_oe) for subsequent experiments. Our WB analysis demonstrated the increased expression levels of MDM2, eIF5B, and proteins in EGFR downstream pathways, which indicated a positive feedback effect on the MDM2/eIF5B/PD-L1 axis in PD-L1_oe cells (Fig. 5F). IF staining revealed the clear nuclear localization of PD-L1 in PD-L1_oe cells, as shown in Fig. 5G. We then conducted drug sensitivity tests to discover potential inhibitors for addressing the Osimertinib resistance caused by ARID1A deficiency-mediated PD-L1 nuclear translocation (Fig. 5H and Figure S5B). As previously mentioned, ARID1A_kd cells demonstrated resistance to Osimertinib. Furthermore, these cells also exhibited resistance to Crizotinib (Figure S5C). PD-L1_oe cells are resistant not only to an EGFR-TKIs (Osimertinib) but also to an anaplastic lymphoma kinase (ALK)-TKI (Lorlatinib) and an autophagy inhibitor (3-MA). Fortunately, similar to ARID1A_kd cells (Fig. 1H and Figure S5D), PD-L1_oe cells showed sensitivity to an EZH2 inhibitor (GSK-126) (Fig. 5H). In addition, PD-L1_oe cells exhibited responsiveness to an Importin-β1 inhibitor (Importazole), a proteasome inhibitor (MG-132), small molecule PD-L1 inhibitors (ARB-272572 and Fraxinellone), and Amlodipine. However, PD-L1_oe cells did not exhibit increased sensitivity to an autophagy inducer (Rapamycin), an E2F1 inhibitor (Simvastatin), or a PD1/PD-L1 interaction inhibitor (BMS-1166).

To investigate the downstream effects of nPD-L1, we conducted further targeted sequencing. We performed ATAC-seq and RNA-seq to analyze chromatin accessibility and RNA expression profiles, respectively, in PD-L1_oe cells (RNA-seq 1) and ARID1A_kd cells (RNA-seq 2 and RNA-seq 3). Our findings suggested that RASGEF1A may be a coregulated target molecule of both PD-L1 and ARID1A, and we observed increased chromatin accessibility and mRNA transcription of RASGEF1A in PD-L1_oe cells (Figure S5E-S5F). To validate these results, we performed WB analysis, which confirmed that PD-L1_oe upregulates the expression of both RASGEF1A and Ras (Fig. 5F). However, in PD-L1_oe cells with ARID1A overexpression (ARID1A_oe), this upregulation was reversed. Similar expression patterns were also observed for MDM2, eIF5B, and Importin-β1 (Fig. 5I). In addition, ARID1A_kd upregulated the expression of RASGEF1A and Ras (Figure S5G).

### 3.7 Nuclear eIF5B/PD-L1/CD44 complex enhances the transcription of RASGEF1A, which is responsive for Ras signaling activation and Osimertinib resistance

The mechanism by which nPD-L1 initiates the transcription of RASGEF1A is still unknown and requires further investigation. We have observed a strong association between CD44 mRNA and protein with PD-L1 through the use of RIP-seq and Co-IP analysis (Fig. 5D). The expression corrrelaiton between PD-L1 and CD44 was further supported by the TIMER database (Figure S5H). Interestingly, we have also discovered that PD-L1 may be responsible for the transportation of CD44 into the nucleus supported by eIF5B and Importin-β1, which has not been previously reported to the best of our knowledge (Fig. 5C and Figure S4A). Additionally, the ARID1A deficient in LUAD cells has led to the upregulation of CD44, as demonstrated in Figure S1C and Figure S5I. This upregulation occurs through the involvement of the EZH2/PTEN/E2F1 axis (Fig. 4H and Figure S3H) and the downstream MDM2/eIF5B/PD-L1 axis (Fig. 5A and Figure S3K-S3M).

In this study, we designated the cluster of misplaced proteins in nucleus, such as PD-L1 and CD44, together with the nuclear translocation facilitators, like eIF5B, as the "nPD-L1 complex". Members of this complex interacts with one another and exhibits distinct functions when compared to their original cellular locations. We utilized a luciferase assay to further examine the role of the nPD-L1 complex. The assay results revealed that an PD-L1_oe could trigger the activation of the RASGEF1A promoter. However, an overexpression of CD44 did not elicit this same response (Fig. 5J). ChIP-seq analysis uncovered that PD-L1 did not possess the ability to bind to DNA. Conversely, CD44 was identified as a potential transcription factor (the enrichment analysis for DNA binding to CD44 can be seen in Figure S5J) and was found to bind to the RASGEF1A DNA in the transcription start site (TSS) region of the promoter (Fig. 5K). Altogether, our findings suggest that PD-L1 interacts with CD44, translocates into the nucleus facilitated by eIF5B and Importin-β1, and activates the RASGEF1A promoter through the transcription factor function of CD44.

**Fig. 6:**
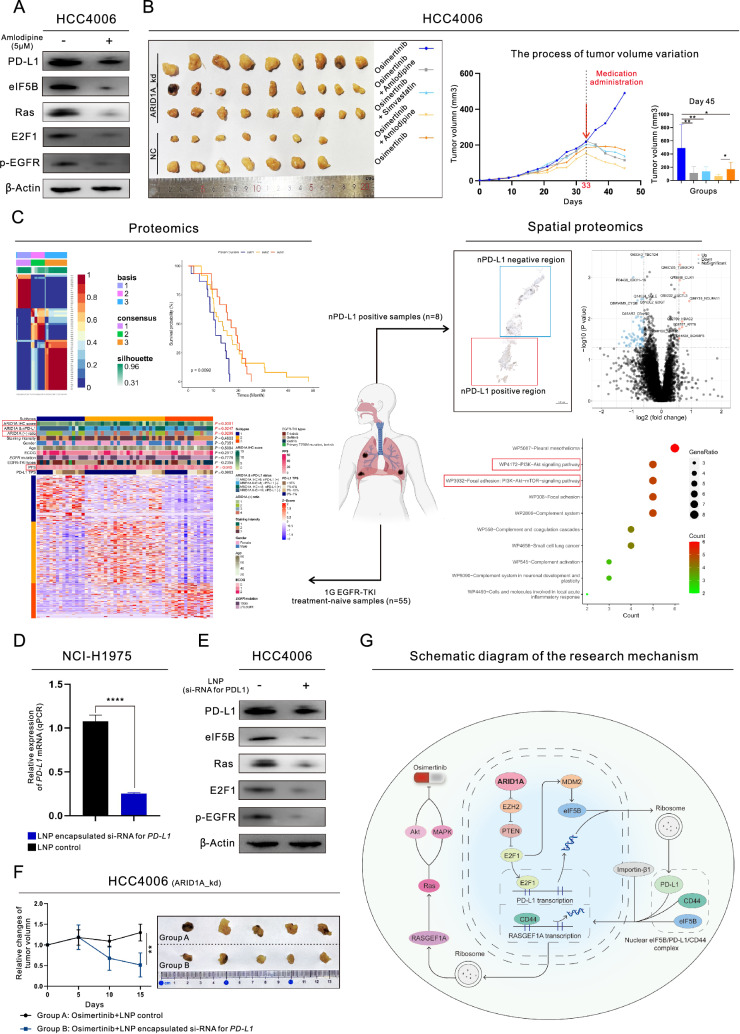
Validation of the findings in this study using an in vivo xenograft model involving lipid nanoparticles (LNPs) and spatial proteomic analysis. **A** Western blot (WB) analysis demonstrated the changes in the expression of proteins in LUAD cells treated with Amlodipine (5 μM); **B** In vivo experiments for the validation of the results of this study (The intervention was initiated on the 33rd day. Dosage and administration, Osimertinib: oral gavage, 5 mg/kg/day; Amlodipine: intraperitoneal injection, 10 mg/kg/day; Simvastatin: oral gavage, 30 mg/kg/day, the ARID1A_kd mouse model consists of 9 subjects in each treatment group, while the control group comprises 7 subjects per treatment group); **C** Spatial proteomic analysis and proteomic analysis for the validation of nuclear PD-L1 function; **D** Treatment with LNPs containing a small interfering RNA (siRNA) for PD-L1 decreased the transcription of PD-L1 (qPCR); **E** WB analysis demonstrated the changes in the expression of proteins in LUAD cells treated with LNPs (2.5 μg/mL, each group consists of five specimens); **F** In vivo experiments for LNP treatment (0.5 mg/kg/day); **G** Schematic diagram for this study

We conducted additional in vivo and in vitro experiments and analyzed the spatial distribution of proteins to validate our findings. We discovered that PD-L1_oe cells were responsive to Amlodipine, a known promoter of autophagy that accelerates the degradation of PD-L1 [15]. Using WB analysis, we also found that Amlodipine significantly reduced the expression of PD-L1 and nuclear location of EZH2 and E2F1 (Figure S5K, Fig. 6A and Figure S6A), similar to the effects of GSK-126 and Importazole (Figure S5K). Moreover, we conducted in vivo experiments (Fig. 6B) and found that ARID1A_kd cells showed significant resistance to Osimertinib, but this resistance was reversed by combining Simvastatin or Amlodipine with Osimertinib treatment. Furthermore, the combination therapy with Amlodipine exhibited increased effectiveness even in the control group. Subsequently, 55 untreated samples were employed for molecular categorization based on proteomic analysis and clinical prognostic analysis. Our findings revealed a notable correlation between the molecular categorization and the expression of ARID1A, as well as the classification based on ARID1A expression and nPD-L1 status (Fig. 6C, left). Then, in 8 samples that tested positive for nPD-L1, we analyzed the differences between the nPD-L1-positive and nPD-L1-negative regions. Our findings indicated that these two regions exhibited significant variations in the ability to regulate the PI3K/Akt/mTOR signaling pathway (Fig. 6C, right). To further validate these results, we utilized lipid nanoparticles (LNPs) containing a small interfering RNA (si-RNA) specifically targeting PD-L1 (si_PD-L1). The si_PD-L1 sequence used in the experiment is provided in Table S2. si-PD-L1 LNPs effectively inhibited the transcription (Fig. 6D) and translation (Fig. 6E and Figure S6B) of PD-L1. Additionally, si_PD-L1 LNP treatment led to a decrease in the expression of MDM2 and eIF5B and inhibited the mTOR pathway downstream of EGFR. Through in vivo experimentation, we observed a synergistic effect in the treatment of ARID1A deficient LUAD with the combination therapy of Osimertinib and si_PD-L1 LNP treatment, as demonstrated in Fig. 6F. The schematic diagram for this study is shown in Fig. 6G. To further validate the role of PD-L1 in the Osimertinib resistance induced by ARID1A_kd, we conducted in vitro experiments which confirmed that PD-L1_kd can inhibit the activation of resistance-related pathways triggered by ARID1A_kd and reverse Osimertinib resistance (Figure S6C-S6D). Lastly, we conducted further validation of the IC50 (6.57 µM versus 14.66 µM, P < 0.0001) to Osimertinib and the relevant pathways in ARID1A_ko cell line, confirming the association between the loss of ARID1A and resistance to Osimertinib (Figure S6E-S6G).

## Discussion

It is imperative to investigate the unidentified mechanisms of Osimertinib resistance and to shed new light on the treatment strategies for overcoming Osimertinib resistance. Our proteomic analysis revealed the absence of ARID1A in Osimertinib resistant tissues, leading us to conduct further experiments to identify a novel target for overcoming Osimertinib resistance. Given the potential synthetic lethality relationship between ARID1A and EZH2 in cancer cells, the current evidence suggests that EZH2 could be a new target for synthetic lethality that can be targeted with drugs in cancers with ARID1A deficiency [5, 6, 16], as confirmed in this study. Additionally, ARID1A is commonly believed to regulate the Akt/mTOR pathway by inhibiting phosphoinositide-3-kinase interacting protein 1 (PIK3IP1) [5, 17]. The present study uncovers a new EZH2/PTEN/E2F1 axis that participates in the regulation of the Akt/mTOR pathway and is responsible for the modulation of programmed cell death, as supported by our previous result [18], which also explains why cancer cells with ARID1A deficiency would be sensitive to EZH2 inhibitors. Additionally, the EZH2/PTEN/E2F1 axis is involved in the regulation of MDM2 expression, and MDM2 expression is upregulated in ARID1A deficient cancers via this axis, which promotes MDM2-mediated P53 degradation and exacerbates the malignant phenotype of these cancers. MDM2 is a widely recognized oncogene that is involved in disease progression and resistance to several types of anticancer therapeutics [13]. Our previous studies have shown that MDM2 amplification in LUAD patients with sensitizing EGFR mutations can lead to resistance to 1G EGFR-TKIs, thus indicating that MDM2 is a potential target for the treatment of EGFR mutant LUAD [19]. In this study, our results showed that inhibiting MDM2 can indeed attenuate the enhanced malignant phenotypes induced by ARID1A deficiency, including cancer progression and Osimertinib resistance. Importantly, it has been proposed that MDM2 expression could be an independent predictor of poor efficacy of Osimertinib treatment.

In this study, we observed a significant increase in the expression of PD-L1 in ARID1A-deficient tumor cell. PD-L1 expression is regulated by the EZH2/PTEN/E2F1 axis and the downstream target MDM2, with E2F1 acting as an important transcription factor. Our findings shed new light on the MDM2/eIF5B/PD-L1 axis, which plays a role in controlling PD-L1 expression and nuclear translocation. Importantly, we discovered that the nuclear translocation of PD-L1 requires the assistance of a helper protein, and in this study, we propose that eIF5B serves as the helper protein for PD-L1. It not only regulates the expression of PD-L1 but also interacts with PD-L1 and facilitates its nuclear translocation through binding to Importin-β1. Importantly, we found that PD-L1 could interacted with CD44 and bring it into the nucleus (we designated them as the nPD-L1 complex) and functioned as a potential transcription factor. Specifically, nPD-L1 complex directly increases the activity of RASGEF1A by activating its promoter. Based on a previous study on RASGEF1A [20], RASGEF1A may be able to increase the activity of the Ras signaling pathway, which promotes the growth and progression of cancer cells. Therefore, in this study, we believe that in ARID1A-deficient tumor cells, nPD-L1 is signaled to activate RASGEF1A and the Ras signaling pathway, ultimately leading to resistance to Osimertinib. Fortunately, We identified a collection of pharmacological agents that may offer therapeutic advantages for patients with ARID1A deficient LUAD, as supported by relevant mechanistic studies. In this research, we utilized two cardiovascular-related agents, specifically, Simvastatin (recognized as an E2F1 inhibitor [21]) and Amlodipine (known as autophagy promoter that acelerates the PD-L1 degradation [15]), as supplementary treatments for patients with ARID1A deficient LUAD undergoing Osimertinib treatment. Both of these drugs reversed the suppression of programmed cell death induced by ARID1A deficiency and increased the effectiveness of Osimertinib without causing any significant adverse effects. Importantly, we developed the si_PDL1 LNP as an effective treatment strategy for adjunctive therapy in ARID1A deficient LUAD, which proved to be a compelling approach for enhancing the efficacy of Osimertinib. LNP, as a biodegradable material within the human body, offers numerous advantages such as a high encapsulation efficiency for nucleic acids and an ability to effectively transfect cells. It also demonstrates excellent tissue permeability, low cytotoxicity, and minimal immunogenicity, which enhances its suitability for drug delivery. These benefits establish LNP as an exceptional delivery system for nucleic acid-based therapeutics [22, 23]. The aforementioned research data further validates the significance of our research direction in the subsequent development of targeted therapies for LUAD. Furthermore, based on previous studies and the results of this research, the significant role of nPD-L1 in regulating cell proliferation and drug resistance has been highlighted. To date, there have been no reports regarding the resistance associated with other medications. Our current study focuses exclusively on the specific role of nPD-L1 in resistance to targeted therapies. Despite this, the core mechanisms suggest that nPD-L1 may exert considerable regulatory effects on other therapeutic approaches, including alterations in the tumor immunomicroenvironment (such as the interactions among mTOR signaling, autophagy, type I interferon, and immune cells) and the efficacy of immunotherapy [2, 24]. This highlights the need for additional investigation. In this research, we examined the specific targets and mechanisms responsible for the resistance to Osimertinib caused by ARID1A deficiency, alongside the design and synthesis of relevant LNP liposomal formulations. However, there is a significant lack of trials and studies assessing the efficacy and safety of these formulations within the broader population. Going forward, we will prioritize the translational aspect of our research outcomes and will develop advanced drug delivery systems leveraging LNP technology to enhance drug targeting, improve therapeutic effectiveness, and minimize potential adverse effects. Furthermore, it is crucial to recognize that we did not conduct clinical trials to validate our findings, which will be a primary focus of our upcoming research initiatives.

## Conclusion

In this study, we discovered the role of ARID1A deficiency in cancer progression and Osimertinib resistance in LUAD. Mechanistically, ARID1A deficiency affects the EZH2/PTEN/E2F1 axis, which in turn regulates the expression of MDM2 and leads to the degradation of P53, preventing programmed cell death. Additionally, we identified a new pathway, the MDM2/eIF5B/PD-L1 axis, downstream of the EZH2/PTEN/E2F1 axis, that controls the expression and nuclear translocation of PD-L1. We revealed the role of nPD-L1 complex in triggering RASGEF1A expression and Ras signaling pathway activity, leading to resistance to Osimertinib in ARID1A deficient LUAD.

## Supplementary Information

Additional file 1.

## Data Availability

No datasets were generated or analysed during the current study.

## Abbreviations

LUAD: Lung adenocarcinoma
TKIs: Tyrosine kinase inhibitors
EGFR: Epidermal growth factor receptor
SWI/SNF: Switch/sucrose nonfermenting
ARID1A: AT-rich interaction domain 1A
EZH2: Zeste 2 polycomb repressive complex 2 subunit
1G: First-generation
3G: Third-generation
PD-L1: Programmed cell death ligand 1
PD-1: Programmed cell death-1
nPD-L1: Nuclear PD-L1
NSCLC: Non-small cell lung cancer
RASGEF1A: RasGEF domain family member 1A
IHC: Immunohistochemical
RNA-seq: RNA sequencing
MS: Mass spectrometry
ATAC-seq: Assay for transposase-accessible chromatin using sequencing
RIP & RIP-seq: RNA immunoprecipitation and sequencing
TEM: Transmission electron microscopy
IC50: Inhibitory concentration
WB: Western blotting
qPCR: Quantitative PCR
Co-IP: Coimmunoprecipitation
IF: Immunofluorescence
PFS: Progression-free survival
CT: Computed tomography
MRI: Magnetic resonance imaging
CR/PR: Complete/partial response
ARID1A_kd: ARID1A-knockdown
NC: Negative control
DEPs: Differentially expressed proteins
Ub: Ubiquitin
MDM2-kd: MDM2 knockdown
TPS: Tumor proportion score
NoD: NucleOlar localization sequence Detector
NoLS: Nucleolar localization sequence
TSS: Transcription start site
LNP: Lipid nanoparticle
