# Supplementary material for: A cohort-based multi-omics identifies nuclear translocation of eIF5B /PD-L1/CD44 complex as the target to overcome Osimertinib resistance of ARID1A-deficient lung adenocarcinoma
Source: Exp Hematol Oncol. 2025 Jan 7;14:3. doi: 10.1186/s40164-024-00594-4 (PMC11705878; doi:10.1186/s40164-024-00594-4)
Supplement: Supplementary file 1 — Additional file 1. [file 40164_2024_594_MOESM1_ESM.zip › New folder/figure S1.pdf]

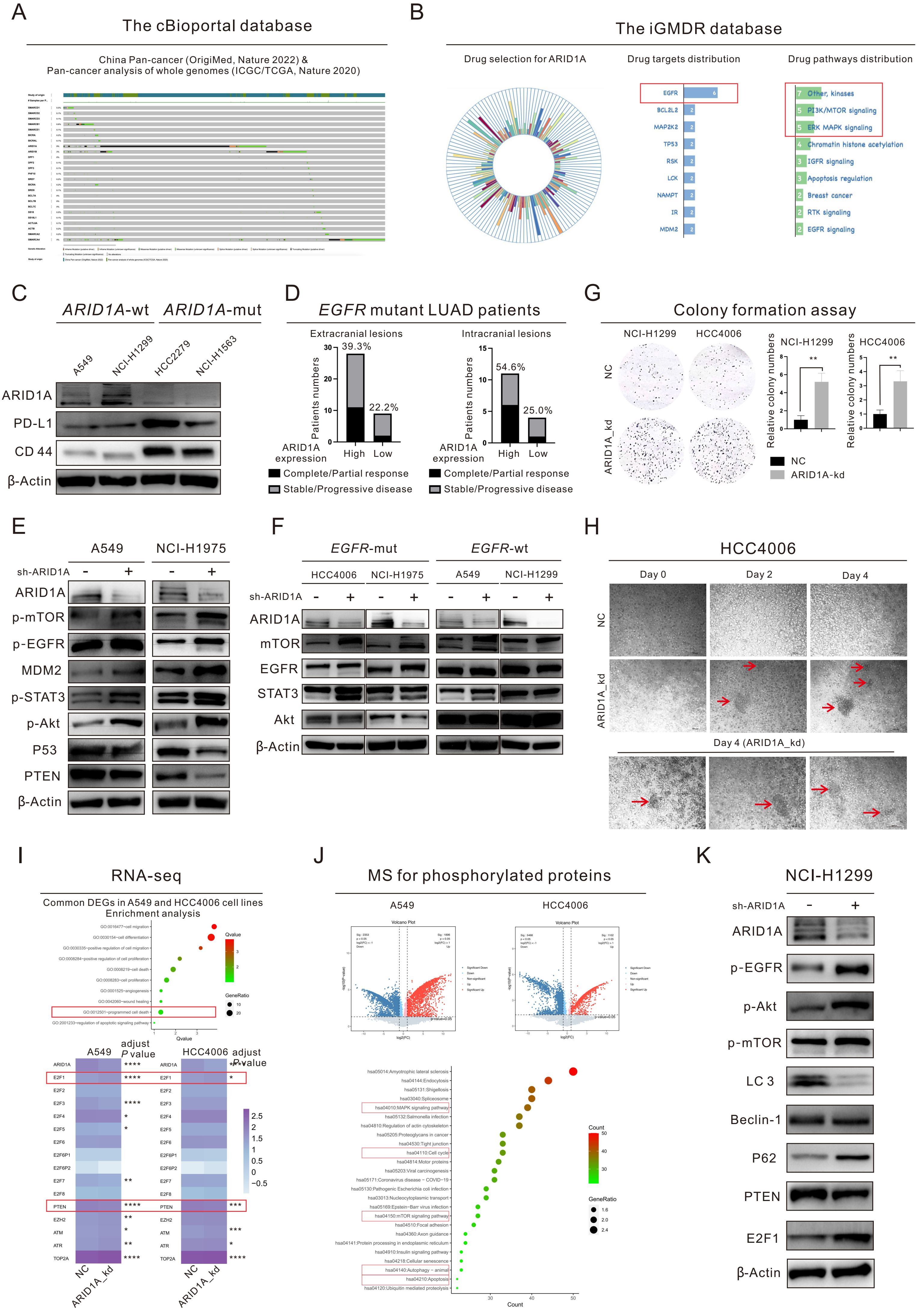

A
The cBioportal database
China Pan-cancer (OrigiMed, Nature 2022) &
Pan-cancer analysis of whole genomes (ICGC/TCGA, Nature 2020)
B
The iGMDR database
Drug selection for ARID1A
Drug targets distribution
Drug pathways distribution
EGFR
BCL2L2
MAP2K2
TP53
RSK
LCK
NAMPT
IR
MDM2
Other, kinases
PI3K/MTOR signaling
ERK MAPK signaling
Chromatin histone acetylation
IGFR signaling
Apoptosis regulation
Breast cancer
RTK signaling
EGFR signaling
C
ARID1A-wt
ARID1A-mut
A549
NCI-H1299
HCC2279
NCI-H1563
ARID1A
PD-L1
CD 44
β-Actin
D
EGFR mutant LUAD patients
Extracranial lesions
Intracranial lesions
Patients numbers
39.3%
22.2%
54.6%
25.0%
ARID1A expression
High
Low
Complete/Partial response
Stable/Progressive disease
G
Colony formation assay
NCI-H1299
HCC4006
NC
ARID1A_kd
Relative colony numbers
**
NC
ARID1A-kd
E
A549
NCI-H1975
sh-ARID1A
ARID1A
p-mTOR
p-EGFR
MDM2
p-STAT3
p-Akt
P53
PTEN
β-Actin
F
EGFR-mut
EGFR-wt
HCC4006
NCI-H1975
A549
NCI-H1299
sh-ARID1A
ARID1A
mTOR
EGFR
STAT3
Akt
β-Actin
H
HCC4006
Day 0
Day 2
Day 4
NC
ARID1A_kd
Day 4 (ARID1A_kd)
I
RNA-seq
Common DEGs in A549 and HCC4006 cell lines
Enrichment analysis
GO:0016477~cell migration
GO:0030154~cell differentiation
GO:0030335~positive regulation of cell migration
GO:0008284~positive regulation of cell proliferation
GO:0008219~cell death
GO:0008283~cell proliferation
GO:0001525~angiogenesis
GO:0042060~wound healing
GO:0012501~programmed cell death
GO:2001233~regulation of apoptotic signaling pathway
Qvalue
GeneRatio
A549
HCC4006
adjust P value
ARID1A
E2F1
E2F2
E2F3
E2F4
E2F5
E2F6
E2F6P1
E2F6P2
E2F7
E2F8
PTEN
EZH2
ATM
ATR
TOP2A
NC
ARID1A_kd
J
MS for phosphorylated proteins
A549
HCC4006
Volcano Plot
hsa05014:Amyotrophic lateral sclerosis
hsa04144:Endocytosis
hsa05131:Shigellosis
hsa03040:Spliceosome
hsa04010:MAPK signaling pathway
hsa05132:Salmonella infection
hsa04810:Regulation of actin cytoskeleton
hsa05205:Proteoglycans in cancer
hsa04530:Tight junction
hsa04110:Cell cycle
hsa04814:Motor proteins
hsa05203:Viral carcinogenesis
hsa05171:Coronavirus disease - COVID-19
hsa05130:Pathogenic Escherichia coli infection
hsa03013:Nucleocytoplasmic transport
hsa05169:Epstein-Barr virus infection
hsa04150:mTOR signaling pathway
hsa04510:Focal adhesion
hsa04360:Axon guidance
hsa04141:Protein processing in endoplasmic reticulum
hsa04910:Insulin signaling pathway
hsa04218:Cellular senescence
hsa04140:Autophagy - animal
hsa04210:Apoptosis
hsa04120:Ubiquitin mediated proteolysis
Count
GeneRatio
K
NCI-H1299
sh-ARID1A
ARID1A
p-EGFR
p-Akt
p-mTOR
LC 3
Beclin-1
P62
PTEN
E2F1
β-Actin
