# Supplementary material for: A cohort-based multi-omics identifies nuclear translocation of eIF5B /PD-L1/CD44 complex as the target to overcome Osimertinib resistance of ARID1A-deficient lung adenocarcinoma
Source: Exp Hematol Oncol. 2025 Jan 7;14:3. doi: 10.1186/s40164-024-00594-4 (PMC11705878; doi:10.1186/s40164-024-00594-4)
Supplement: Supplementary file 1 — Additional file 1. [file 40164_2024_594_MOESM1_ESM.zip › New folder/figure S4.pdf]

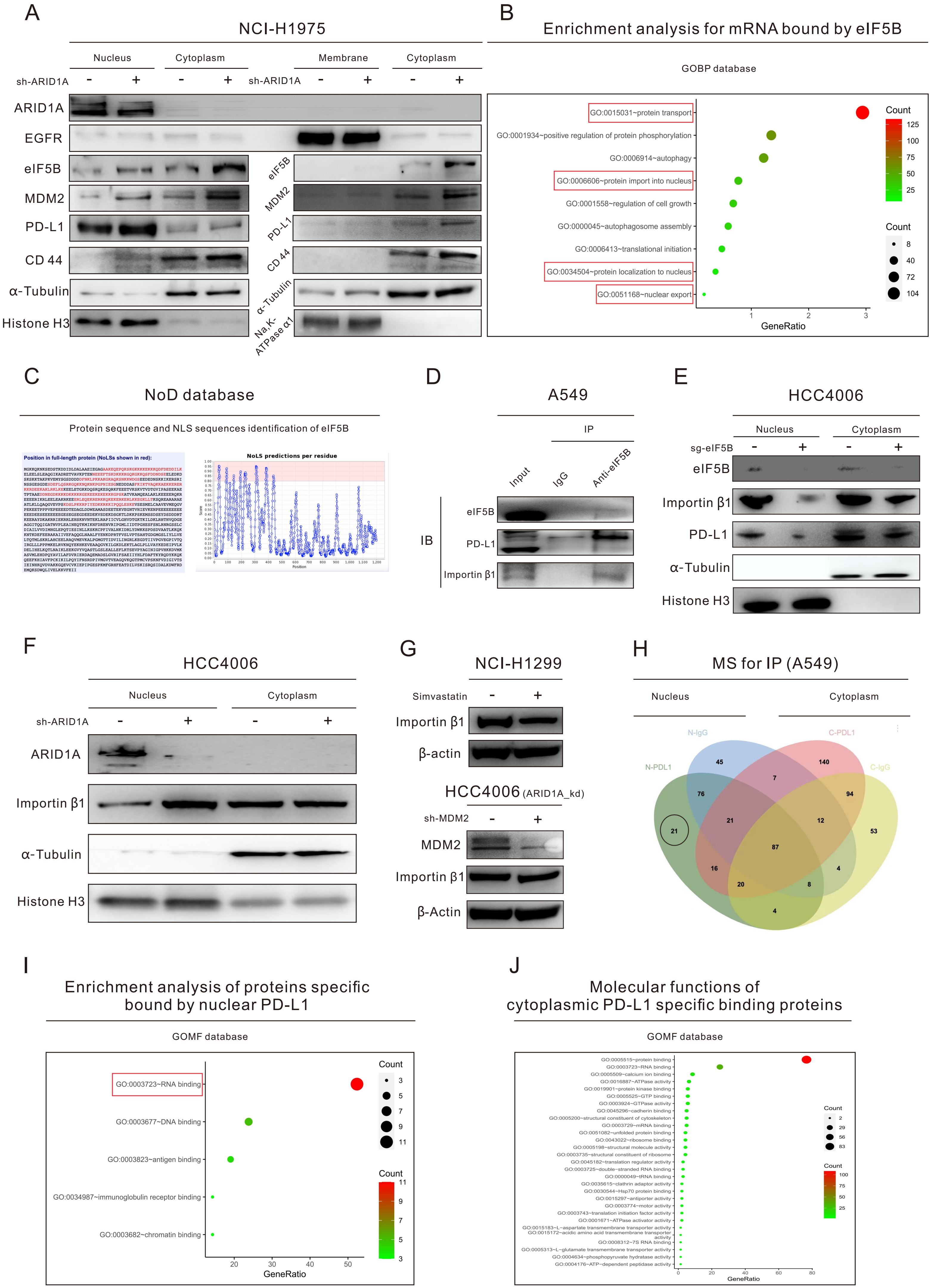
A
NCI-H1975
Nucleus
Cytoplasm
Membrane
Cytoplasm
sh-ARID1A - + - +
sh-ARID1A - + - +
ARID1A
EGFR
eIF5B
MDM2
PD-L1
CD 44
α-Tubulin
Histone H3
eIF5B
MDM2
PD-L1
CD 44
α-Tubulin
Na,K-ATPase α1
B
Enrichment analysis for mRNA bound by eIF5B
GOBP database
GO:0015031~protein transport
GO:0001934~positive regulation of protein phosphorylation
GO:0006914~autophagy
GO:0006606~protein import into nucleus
GO:0001558~regulation of cell growth
GO:0000045~autophagosome assembly
GO:0006413~translational initiation
GO:0034504~protein localization to nucleus
GO:0051168~nuclear export
GeneRatio
1
2
3
Count
125
100
75
50
25
Count
8
40
72
104
C
NoD database
Protein sequence and NLS sequences identification of eIF5B
Position in full-length protein (NoLSs shown in red):
NoLS predictions per residue
Position
D
A549
IP
Input
IgG
Anti-eIF5B
IB
eIF5B
PD-L1
Importin β1
E
HCC4006
Nucleus
Cytoplasm
sg-eIF5B - + - +
eIF5B
Importin β1
PD-L1
α-Tubulin
Histone H3
F
HCC4006
Nucleus
Cytoplasm
sh-ARID1A - + - +
ARID1A
Importin β1
α-Tubulin
Histone H3
G
NCI-H1299
Simvastatin - +
Importin β1
β-actin
HCC4006 (ARID1A_kd)
sh-MDM2 - +
MDM2
Importin β1
β-Actin
H
MS for IP (A549)
Nucleus
Cytoplasm
N-IgG
C-PDL1
N-PDL1
C-IgG
45
140
7
76
94
21
21
12
87
53
16
4
20
8
4
I
Enrichment analysis of proteins specific bound by nuclear PD-L1
GOMF database
GO:0003723~RNA binding
GO:0003677~DNA binding
GO:0003823~antigen binding
GO:0034987~immunoglobulin receptor binding
GO:0003682~chromatin binding
GeneRatio
20
30
40
50
Count
3
5
7
9
11
Count
11
9
7
5
3
J
Molecular functions of cytoplasmic PD-L1 specific binding proteins
GOMF database
GO:0005515~protein binding
GO:0003723~RNA binding
GO:0005509~calcium ion binding
GO:0016887~ATPase activity
GO:0019901~protein kinase binding
GO:0005525~GTP binding
GO:0003924~GTPase activity
GO:0045296~cadherin binding
GO:0005200~structural constituent of cytoskeleton
GO:0003729~mRNA binding
GO:0051082~unfolded protein binding
GO:0043022~ribosome binding
GO:0005198~structural molecule activity
GO:0003735~structural constituent of ribosome
GO:0045182~translation regulator activity
GO:0003725~double-stranded RNA binding
GO:0000049~tRNA binding
GO:0035615~clathrin adaptor activity
GO:0030544~Hsp70 protein binding
GO:0015297~antiporter activity
GO:0003774~motor activity
GO:0003743~translation initiation factor activity
GO:0001671~ATPase activator activity
GO:0015183~L-aspartate transmembrane transporter activity
GO:0015172~acidic amino acid transmembrane transporter activity
GO:0008312~7S RNA binding
GO:0005313~L-glutamate transmembrane transporter activity
GO:0004634~phosphopyruvate hydratase activity
GO:0004176~ATP-dependent peptidase activity
GeneRatio
0
20
40
60
80
Count
2
29
56
83
Count
100
75
50
25
