# Supplementary material for: A cohort-based multi-omics identifies nuclear translocation of eIF5B /PD-L1/CD44 complex as the target to overcome Osimertinib resistance of ARID1A-deficient lung adenocarcinoma
Source: Exp Hematol Oncol. 2025 Jan 7;14:3. doi: 10.1186/s40164-024-00594-4 (PMC11705878; doi:10.1186/s40164-024-00594-4)
Supplement: Supplementary file 1 — Additional file 1. [file 40164_2024_594_MOESM1_ESM.zip › New folder/figure S5.pdf]

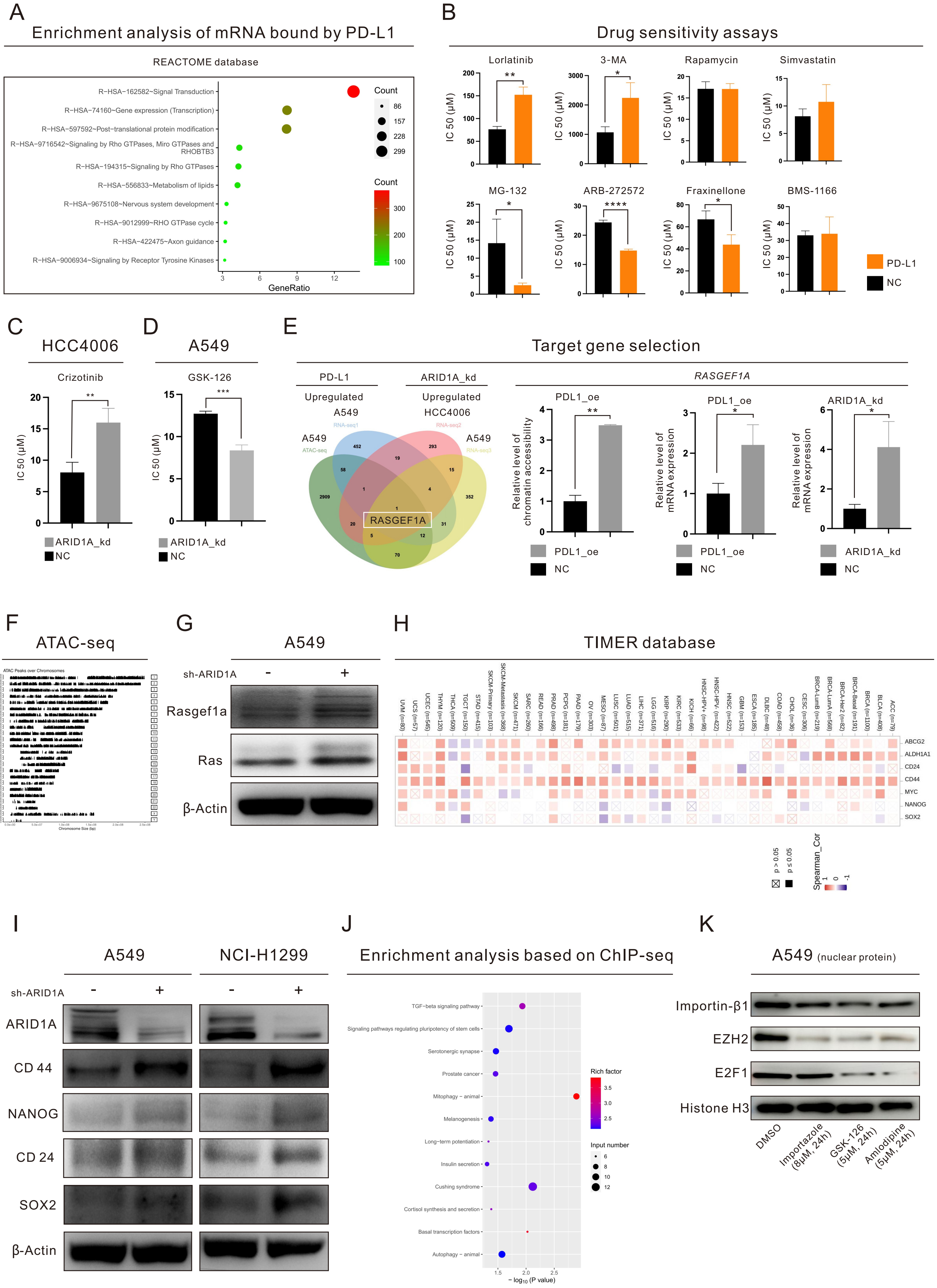

A
Enrichment analysis of mRNA bound by PD-L1
REACTOME database
R−HSA−162582~Signal Transduction
R−HSA−74160~Gene expression (Transcription)
R−HSA−597592~Post−translational protein modification
R−HSA−9716542~Signaling by Rho GTPases, Miro GTPases and RHOBTB3
R−HSA−194315~Signaling by Rho GTPases
R−HSA−556833~Metabolism of lipids
R−HSA−9675108~Nervous system development
R−HSA−9012999~RHO GTPase cycle
R−HSA−422475~Axon guidance
R−HSA−9006934~Signaling by Receptor Tyrosine Kinases
GeneRatio
Count
86
157
228
299
Count
300
200
100
B
Drug sensitivity assays
Lorlatinib
3-MA
Rapamycin
Simvastatin
MG-132
ARB-272572
Fraxinellone
BMS-1166
IC 50 (μM)
PD-L1
NC
C
HCC4006
Crizotinib
IC 50 (μM)
ARID1A_kd
NC
D
A549
GSK-126
IC 50 (μM)
ARID1A_kd
NC
E
Target gene selection
PD-L1
ARID1A_kd
Upregulated
A549
Upregulated
HCC4006
A549
ATAC-seq
RNA-seq1
RNA-seq2
A549
RNA-seq3
RASGEF1A
RASGEF1A
PDL1_oe
PDL1_oe
ARID1A_kd
Relative level of chromatin accessibility
Relative level of mRNA expression
Relative level of mRNA expression
PDL1_oe
NC
PDL1_oe
NC
ARID1A_kd
NC
F
ATAC-seq
ATAC Peaks over Chromosomes
Chromosome Size (bp)
G
A549
sh-ARID1A
Rasgef1a
Ras
β-Actin
H
TIMER database
ABCG2
ALDH1A1
CD24
CD44
MYC
NANOG
SOX2
p > 0.05
p ≤ 0.05
Spearman_Cor
I
A549
NCI-H1299
sh-ARID1A
ARID1A
CD 44
NANOG
CD 24
SOX2
β-Actin
J
Enrichment analysis based on ChIP-seq
TGF−beta signaling pathway
Signaling pathways regulating pluripotency of stem cells
Serotonergic synapse
Prostate cancer
Mitophagy − animal
Melanogenesis
Long−term potentiation
Insulin secretion
Cushing syndrome
Cortisol synthesis and secretion
Basal transcription factors
Autophagy − animal
Rich factor
Input number
− log10 (P value)
K
A549 (nuclear protein)
Importin-β1
EZH2
E2F1
Histone H3
DMSO
Importazole (8μM, 24h)
GSK-126 (5μM, 24h)
Amlodipine (5μM, 24h)
