# Supplementary material for: A cohort-based multi-omics identifies nuclear translocation of eIF5B /PD-L1/CD44 complex as the target to overcome Osimertinib resistance of ARID1A-deficient lung adenocarcinoma
Source: Exp Hematol Oncol. 2025 Jan 7;14:3. doi: 10.1186/s40164-024-00594-4 (PMC11705878; doi:10.1186/s40164-024-00594-4)
Supplement: Supplementary file 1 — Additional file 1. [file 40164_2024_594_MOESM1_ESM.zip › New folder/Supplementary figure legends.docx]

**Supplementary figure legends**

**Figure S1. Supplementary figure 1.** A. The cBioPortal database revealed the mutation frequencies of SWI/SNF complex components in pancancer datasets; B. Drug selection and mechanistic exploration of ARID1A using the iGMDR database; C. Western blot (WB) analysis demonstrated the differences in the expression of proteins between ARID1A mutant and ARID1A wild-type cell lines; D. Complete/partial response and stable/progressive disease rates in lung adenocarcinoma (LUAD) patients treated with Osimertinib (intracranial/extracranial lesions); E-F. WB analysis demonstrated the changes in the expression of proteins in LUAD cells with ARID1A knockdown (ARID1A_kd); G. Colony formation assays in NCI-H1299 and HCC4006 cells with or without ARID1A_kd (the cells were treated with Osimertinib for 14 days); H. 3D culture of HCC4006 cells with or without ARID1A_kd; I. Relative expression and adjusted P values of selected genes in A549 and HCC4006 cells with or without ARID1A_kd, based on RNA-seq; J. Mass spectrometry identification of phosphorylated proteins in A549 and HCC4006 cells with or without ARID1A_kd and the subsequent enrichment analysis; K. WB analysis demonstrated the changes in the expression of proteins in LUAD cells with or without ARID1A_kd.

**Figure S2. Supplementary figure 2.** A. Western blot (WB) analysis demonstrated the changes in the expression of proteins in lung adenocarcinoma (LUAD) cells with PTEN overexpression; B. Schematic diagram showing the relationship between the EZH2/PTEN/E2F1 axis and autophagy; C. Expression correlations according to the GEPIA database; D. Relative expression and adjusted P values for autophagic and apoptotic genes in A549 and HCC4006 cell lines, as determined by RNA-seq; E-F. WB analysis demonstrated the changes in the expression of proteins in LUAD cells with ARID1A knockdown (ARID1A_kd), MDM2 knockdown (MDM2_kd) or treatment with different agents (Simvastatin: 10 µM, 24 h; GSK-126: 5 µM, 24 h; MG-132: 15 µM, 24 h); G. Images of IHC staining for MDM2 in LUAD tissues; H. Prognostic value of MDM2 expression for progression-free survival (PFS) in LUAD patients treated with Osimertinib; I. Statistical analysis of wound healing assays in control HCC4006 cells, HCC4006 cells with ARID1A_kd and HCC4006 cells with combined ARID1A_kd/MDM2_kd; J. Comparison of Osimertinib IC50s in ARID1A_kd HCC4006 cells with or without MDM2_kd using an MTS assay; K. Schematic diagram showing the downstream signaling pathways of ARID1A.

**Figure S3. Supplementary figure 3.** A. Prognostic value of the PD-L1 tumor proportion score (TPS) for progression-free survival (PFS) in patients with first generation EGFR-TKI treatment; B. Prognostic value of nuclear PD-L1 status for PFS in patients with first generation EGFR-TKI treatment; C. Correlation between ARID1A and PD-L1 (CD274) expression according to TISIDB; D. Western blot (WB) analysis demonstrated the changes in the expression of proteins in tumor cells with ARID1A knockdown (ARID1A_kd); E. Immunofluorescence (IF) staining showing the changes in the IF intensity and localization of PD-L1 in tumor cells with ARID1A_kd; F. WB analysis demonstrated the changes in the expression of proteins in tumor cells with PTEN overexpression; G. The GEPIA database and TISIDB demonstrated the positive correlation between EZH2 and PD-L1 (CD274); H-I. WB analysis demonstrated the changes in the expression of proteins in lung adenocarcinoma (LUAD) cells treated with different agents (Simvastatin: 10 µM, 24 h; GSK-126: 5 µM, 24 h; MG-132: 15 µM, 24 h); J. Identification of downstream targets of MDM2 through mass spectrometry, RNA-seq and GEPIA database analyses; K-L. WB analysis demonstrated the changes in the expression of proteins in LUAD cells with and without MDM2 knockdown; M. WB analysis demonstrated the changes in the expression of proteins in LUAD cells with and without eIF5B knockout; N. Enrichment analysis based on the eIF5B RIP-seq data.

**Figure S4. Supplementary figure 4.** A. Separation of nuclear and membrane proteins in tumor cells and Western blot (WB) analysis; B. Enrichment analysis based on the eIF5B RIP-seq data; C.Nucleolar localization sequences (NoLSs) in eIF5B predicted by the NoD database; D. Co-IP of eIF5B in tumor cells; E-F. Separation of nuclear proteins in lung adenocarcinoma (LUAD) cells and WB analysis; G. WB analysis demonstrated the changes in the expression of proteins in LUAD cells with different treatments; H. Mass spectrometry analysis of nuclear and nonnuclear PD-L1 immunoprecipitates in LUAD cells; I. Enrichment analysis based on immunoprecipitation (IP) followed by mass spectrometry for nuclear PD-L1 specific binding proteins; J. Enrichment analysis based on IP followed by mass spectrometry for cytoplasmic PD-L1 specific binding proteins

**Figure S5. Supplementary figure 5.** A. Enrichment analysis of mRNAs bound to PD-L1 using RIP-seq; B-D. The MTS assay demonstrated the sensitivity to relative inhibitors in lung adenocarcinoma (LUAD) cells (B: A549 cell line); E. The downstream target gene for ARID1A and PD-L1 was discovered in LUAD cells through ATAC-seq and RNA-seq analysis; F. The peaks identified on chromosomes through ATAC-seq in PD-L1 overexpressed cells; G&I. Western blot (WB) analysis demonstrated the changes in the expression of proteins in LUAD cells with ARID1A knockdown; H. The correlation between the expressions of CD274 (PD-L1) and biomarkers of cancer stem cells (TIMER database); J. The enrichment analysis for DNA bounded by CD44 according to ChIP-seq analysis; K. Separation of nuclear proteins and WB analysis in LUAD cells with different treatments.

**Figure S6. Supplementary figure 6.** A-B. Western blot (WB) analysis demonstrated the changes in the expression of proteins in lung adenocarcinoma (LUAD) cells with different treatments (Amlodipine: 5 μM; LNP: 2.5 μg/mL); C. PD-L1 knockdown (PDL1_kd) successfully overcame the resistance to Osimertinib induced by ARID1A knockdown (ARID1A_kd); D. WB analysis revealed that PD-L1_kd inhibited the activation of the Osimertinib-resistance related pathway triggered by ARID1A_kd; E. The IC50 examination for ARID1A knockout (ARID1A_ko) or control group of NCI-H1975 cells; F-G. The WB analysis demonstrated that the ARID1A_ko alters the expression levels of proteins associated with Osimertinib resistance.
