# Supplementary material for: A cohort-based multi-omics identifies nuclear translocation of eIF5B /PD-L1/CD44 complex as the target to overcome Osimertinib resistance of ARID1A-deficient lung adenocarcinoma
Source: Exp Hematol Oncol. 2025 Jan 7;14:3. doi: 10.1186/s40164-024-00594-4 (PMC11705878; doi:10.1186/s40164-024-00594-4)
Supplement: Supplementary file 1 — Additional file 1. [file 40164_2024_594_MOESM1_ESM.zip › New folder/Supplementary file 1.pdf]

A549 cell STR identification report

1. Material handling and inspection methods

Take appropriate **A549** cells (× 10. 1·^{6}) using PureLink® the Genomic the DNA the Mini Kit (U. S. Life K182001 genomic) ahead of the DNA, using the PowerPlex® 18D system (U.S. Promega DC1802) kit was amplified in ABI3500 Genetic Analyzer (U. S. Life3500) Perform testing.

2. Test results

Both the negative and positive control results in the experiment were correct.

The genotyping results of the STR locus and Amelogenin locus of the **A549** cell line are shown in the attached table, and the type map is shown in the attached figure.

Three, analysis description

After amplification of the genomic DNA of the **A549** cell line , the map is clear and the typing results are good.

4. Inspection conclusion

1. The results of cellular STR typing of **A549** cell line DNA showed that no human cells were found in the cell line.

Fork contamination.

2. The cell line DNA typing is found in the ATCC cell bank that matches 100% of its cell typing

The cell line name is **A549** .

Procell
www.procell.com.cn

Attached Table **1** : Genotyping results of **STR** locus and **Amelogenin** locus of cell line **A549**

Cell **A549** (picture number is PC73)		
Marker	***Allele 1***	***Allele 2***
D3S1358	16	16
**THO1**	8	9.3
D21S11	29	29
D18S51	14	17
Penta E	7	11
**D5S818**	11	11
**D13S317**	11	11
**D7S820**	8	11
**D16S539**	11	12
**CSF1PO**	10	12
Penta D	9	9
**AMEL**	X	Y
**vWA**	14	14
D8S1179	13	14
**TPOX**	8	11
FGA	twenty three	twenty three
D6S1043	11	13
D2S1338	twenty four	twenty four
D12S391	18	18
D19S433	13	13
D1S1656	17	18.3

Procell www.procell.com.cn

Figure **1** : ATCC official website **A549** cell **STR** site information

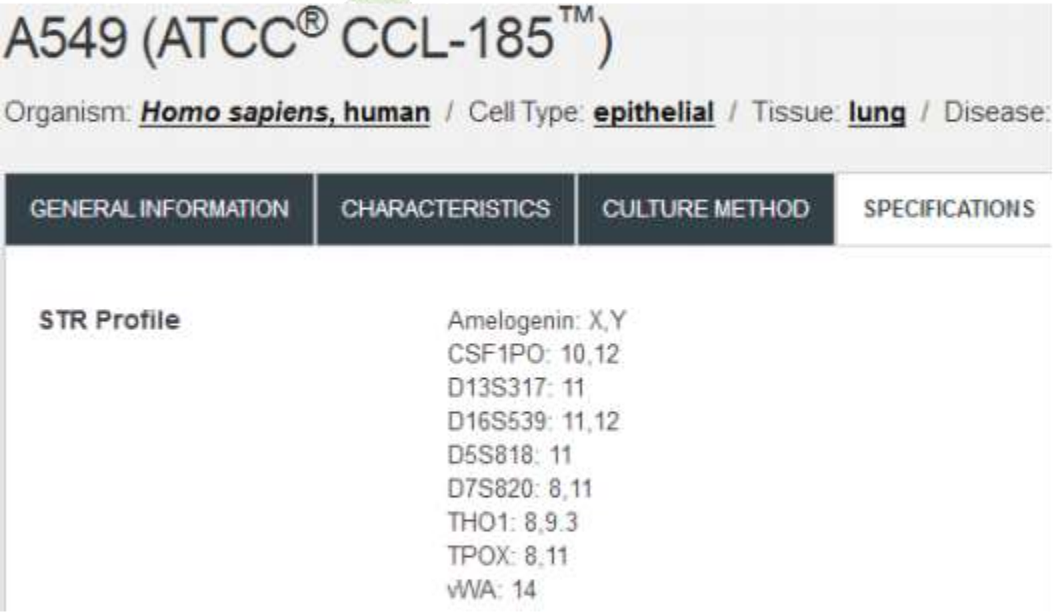

Figure **2** : Genotyping results of **STR** locus and **Amelogenin** locus of **A549** cells

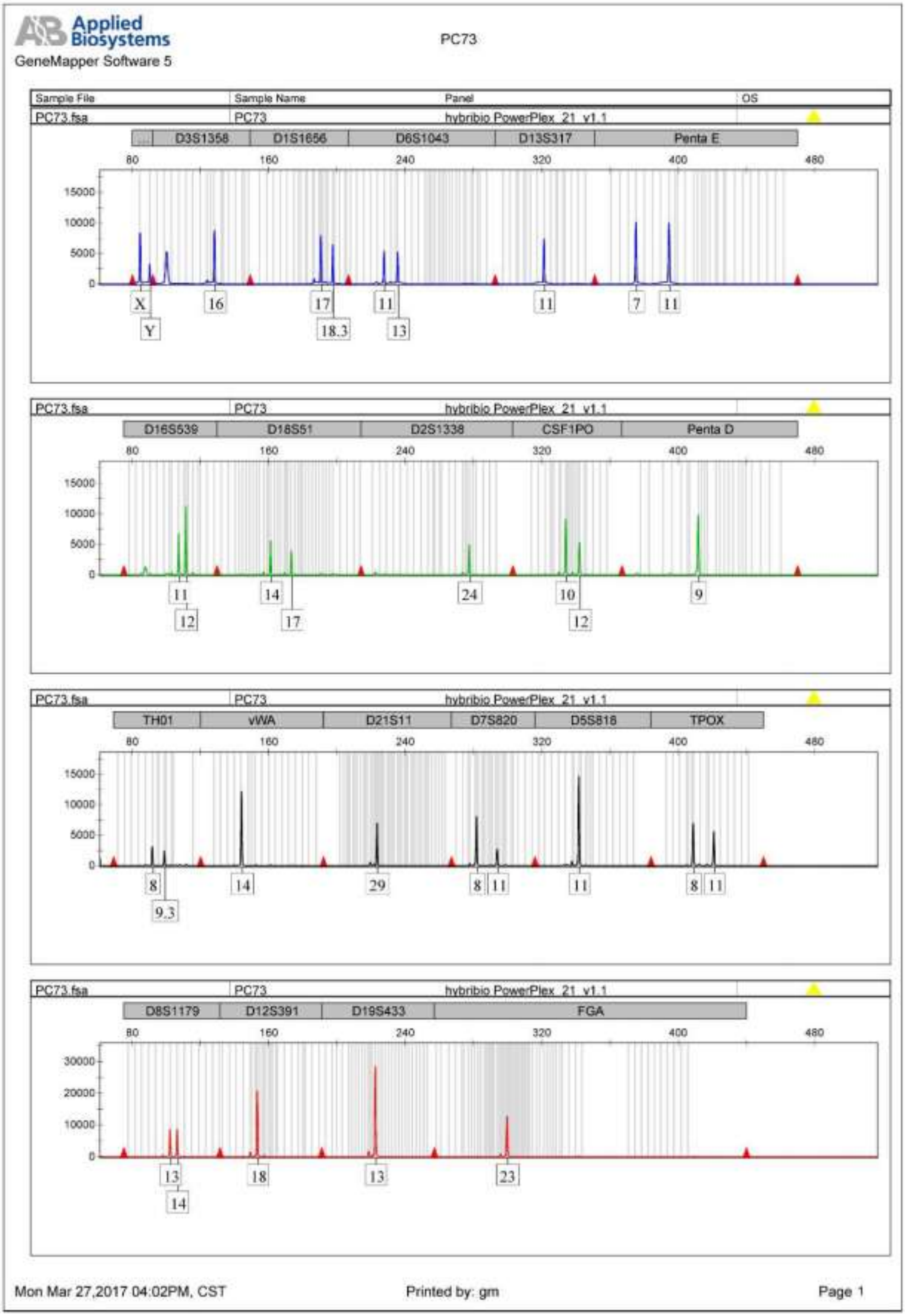

Website: ***www.procell.com.cn***
Phone: 400-650-3656
Email: sales@procell.com.cn

HAKATA®

Cell STR test report

Shanghai Chuanqiu Biological Technology Co., Ltd.

www.chuanqiubio.com

HAKATA®

Cell STR test report

Client : Shanghai Chuanqiu Biological Technology Co., Ltd.

Commission Date : 2020- 11-08

Identification Date : 2020- 11- 12 is

A , the material being tested:

2020- 11-08 received client . 1 cell lines precipitation :

The serial number is "XB-2011091", and the package is marked as "H1299".

It is required to identify whether the cell line is a single-source cell line and whether there is cross-contamination . **Two , sample timber handling and test methods:**

Proper amount of sample material (with microread the Genomic the DNA Kit extract the DNA ,) using MicroReader (TM) 21 is ID the System amplification 20 is th STR loci and gender identification locus , using the ABI 3730xl Genetic Analyzer into rows PCR product was detected using GeneMapperID-X software (Applied Biosystems) for the detection result into the line analysis , with ATCC and DSMZ for comparison database .

Three , test results:

1 Both the negative and positive control results in the experiment are correct;

2 After the DNA amplification of the test sample cell , the pattern is clear , and the typing result is good . The STR site and

The genotyping results of Amelogenin locus are shown in the attached table , and the typing map is shown in the attached figure;

3 The cell does not have multiple allele [1] phenomenon in the 8 alignment sites ;

4 database alignments [2] (show the highest degree of matching results):

	ATCC				DSMZ				Test sample			
Cell name	NCI-H1299Lung CarcinomaHuman				NCI-H1299				H1299			
Cell number	CRL-5803				CRL-5803				XB-2011091			
Amelogenin	X				X	X			X			
D5S818	11				11	11			11			

D13S317	12				12	12			12				
D7S820	10				10	10			10				

HAKATA®

D16S539	12	13			12	13			12	13		
vWA	16	17	18		16	17	18		16	18		
TH01	6	9.3			6	9.3			6	9.3		
TPOX	8				8	8			8			
CSF1PO	12				12	12			12			
Match [3]	92%				0.97							
Matching instructions [4]	Relevant				-							

Description :

1 . In the ATCC and DSMZ cell bank 8 is greater than or equal to a ratio of sites .3 a multi-allelic suggests the possibility of cross-species homology sewage

Pollution ; the presence of 1-2 multiple alleles may be due to cell mutation and is not considered as contamination .

2.Genotyping results included in the test cell with ATCC and DSMZ cell bank (DSMZ Cell library contains a URL connection from ATCC , DSMZ ,

JCRB , and RIKEN this .4 cells library 2455 Cell Lines STR cell line type) in the STR data matches , is

not included in the narrow cell library cells will not match .

.3 . ATCC matching algorithm to match the database is the number of peaks in all tested cell lines / ATCC number of all peaks × 100% , the DSMZ database

The matching algorithm , the EV = number of all peaks match test cell × 2 / (measured cells + number database) all peaks .

4 . Match description : the ATCC cell bank according to the ratio of the ANSI develop international standards , matching cell lines ≥80% when considered with their

There is a correlation , that is , it is derived from a common ancestor cell ; the matching degree is

between 56% and 79% , and the correlation needs to be further verified ; less than 56% , indicating

that the two are not related . When the STR typing of the cell line to be tested is compared with the

DSMZ cell bank , there are only two STR scores

When the types are exactly the same , the matching description is " exact match " , otherwise the matching description is " -" .

Four , conclusions of the examination:

H1299: ① The cell DNA for cell STR typing results show , no cells found in the human small cell cross-contamination . ②The matching rate of STR data between this cell and NCI- H1299Lung CarcinomaHuman cell was 92% . ③The DNA typing of this cell is not found in the DSMZ cell bank that matches 100% of its typing

Beij

Cell .

Operator : Yi Yunhui

(This result is only responsible for this inspection)

Reviewer : Ding Rong

Yuewei Gene Technology Co., Ltd.

Remarks :

1.Effective peak real PCR bands , small peak and non-specific bands in the calculation negligible .

2.This experiment meet the ISO 9001 : , 2015 and the ISO 13485: 2016 quality standards , in accordance with MicroReader (TM) 21 is ID the System the STR kit

The provided experimental methods and analytical methods are tested , and the results are for scientific research use only .

.3.Standards for the Cell Line Authentications : the To Standardize the STR Analysis for Human Cell Line authentication, the American Tissue Culture Collection (ATCC) Standards Development Organization Workgroup published ASN-0002-2011, which recommends the use of at least eight STR loci (TH01, TPOX, vWA, CSF1PO, D16S539, D7S820, D13S317 and D5S818) plus Amelogenin for gender identification for human cell line authentication .

HAKATA®

Attached table : Genotyping results of STR locus and Amelogenin locus of cell H1299

Cell H1299 (Picture No. XB-2011091)		
Marker	Allele 1	Allele 2
D19S433	14	14
D5S818	11	11
D21S11	32.2	32.2
D18S51	16	16
D6S1043	11	11
AMEL	X	X
D3S1358	17	17
D13S317	12	12
D7S820	10	10
D16S539	12	13
CSF1PO	12	12
Penta D	13	13
D2S441	11	13
vWA	16	18
D8S1179	10	13
TPOX	8	8
Penta E	11	11
TH01	6	9.3
D12S391	twenty one	twenty two
D2S1338	twenty three	twenty four
FGA	20	20

BRIEF : Cells H1299 of STR typing pattern

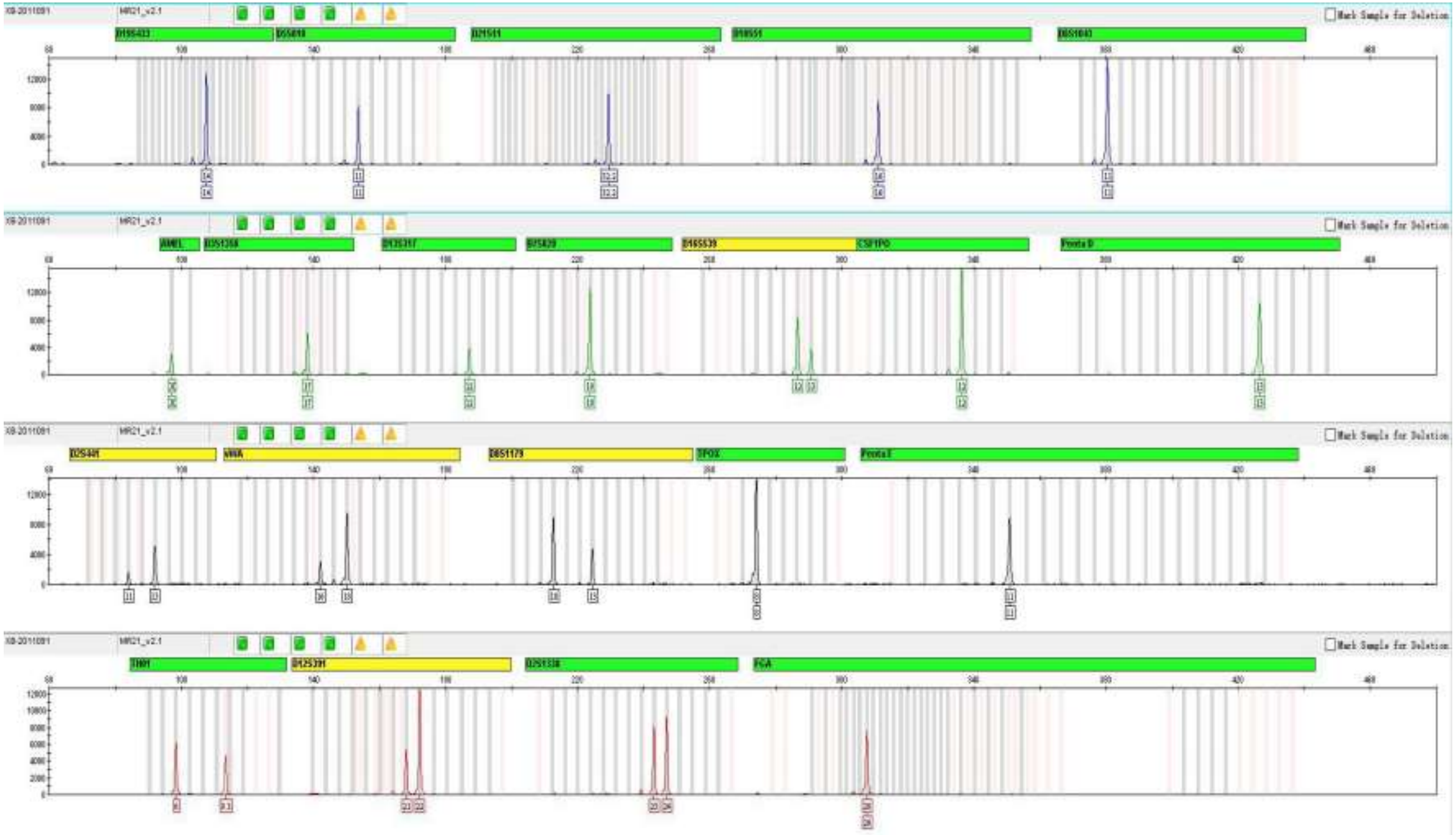

www.chuanqiubio.com

Cell Line Authentication Report

Cell Line Authentication Report

Customer: Weizheng Wu

Quotation Number: 80-1076809638_R2

Completion Date: 11/30/2022

1. **Sample ID: S3**
2. **Original Material: Cell pellet**
3. **Methods:**

1).Genomic DNA was extracted from the cell pellets provided by the customer.

2).Samples, together with positive and negative control were amplified using GenePrint 10 System (Promega).

3). Amplified products were processed using the ABI3730xl Genetic Analyzer.

4). Data were analyzed using GeneMapper4.0 software and then compared with the ATCC, DSMZ, JCRB and RIKEN databases for reference matching.

4. **Results:**

1) 10 Loci STR Profile：

Genetic Site	Customer sample	
(Locus)	S3	
Amelogenin	X	X
CSF1PO	11	12
D13S317	12	12
D16S539	11	13
D5S818	9	12
D7S820	10	11
THO1	6	7
TPOX	11	12
vWA	16	17
D21S11	29	29

AZENTA
LIFE SCIENCES

<<< Search for reference matching with the Cell Bank databases and add the match results. >>>

Result of STR matching analysis by your data.
-DSMZ Profile Database-

EV	Cell No.	Cell name	Locus names								
			D5S818	D13S317	D7S820	D16S539	VWA	TH01	AM	TPOX	CSF1PO
	Query(Your Cell)		9,12	12,12	10,11	11,13	16,17	6,7	X,X	11,12	11,12
1.00(36/36)	CRL-1739	AGS	9,12	12,12	10,11	11,13	16,17	6,7	X,X	11,12	11,12
0.72(26/36)	CRL-2466	CCD-1118SK	12,13	11,12	9,10	11,13	16,17	6,9	X,X	8,11	11,12
0.72(26/36)	CRL-2614	Ect1/E6E7	11,12	12,12	10,10	10,13	16,17	6,10	X,X	8,11	11,12
0.72(26/36)	JCRB1093	PSVK1	12,13	12,12	10,11	9,13	16,16	6,7	X,Y	8,11	11,12
0.72(26/36)	RCB1549	YSCCC	10,12	12,12	10,11	10,11	16,17	7,9	X,X	11,11	10,11
0.72(26/36)	RCB1724	JHSK-rec	11,12	9,9	10,11	11,13	14,16	6,7	X,X	8,12	11,12
0.70(26/37)	426	SW-1710	12,12	12,12	8,11	8,11	16,17	7,9.3,8	X,X	9,11	11,12
0.67(24/36)	654	GI-ME-N	12,12	8,12	10,11	9,12	16,19	6,7	X,X	11,11	11,12
0.67(24/36)	CCL-76	citrullinemia	9,12	10,12	8,11	11,12	17,20	7,9.3	X,X	8,11	11,12
0.67(24/36)	CRL-1622	KLE	9,12	12,12	11,12	11,12	16,16	6,7	X,X	8,11	13,14
0.67(24/36)	CRL-2346	HCC 38BL	9,12	12,14	10,10	10,14	16,17	7,9.3	X,X	9,12	11,12
0.67(24/36)	CRL-2439	CCD 1113SK	12,13	12,12	11,13	11,13	13,17	6,7	X,X	8,9	12,12
0.67(24/36)	CRL-2553	Panc 02.03	12,13	12,12	9,10	11,11	17,17	6,6	X,X	9,12	11,12
0.67(24/36)	CRL-2615	End1/E6E7	11,12	9,12	10,10	10,13	16,17	6,6	X,X	8,11	11,12
0.67(24/36)	CRL-2846	CHON-001	11,12	12,14	12,12	11,13	16,17	6,7	X,X	8,11	10,11
0.67(24/36)	CRL-2847	CHON-002	11,12	12,14	12,12	11,13	16,17	6,7	X,X	8,11	10,11
0.67(24/36)	CRL-2848	CHON-003	11,12	12,14	12,12	11,13	16,17	6,7	X,X	8,11	10,11
0.67(24/36)	CRL-2856	CHON-004	11,12	12,14	12,12	11,13	16,17	6,7	X,X	8,11	10,11
0.67(24/36)	CRL-2857	CHON-005	11,12	12,14	12,12	11,13	16,17	6,7	X,X	8,11	10,11

Summary

Your cell line is considered “identical” to the reference cell line AGS in the ATCC STR database, as the STR profile yields a 100% match.

2) Electrophoretogram

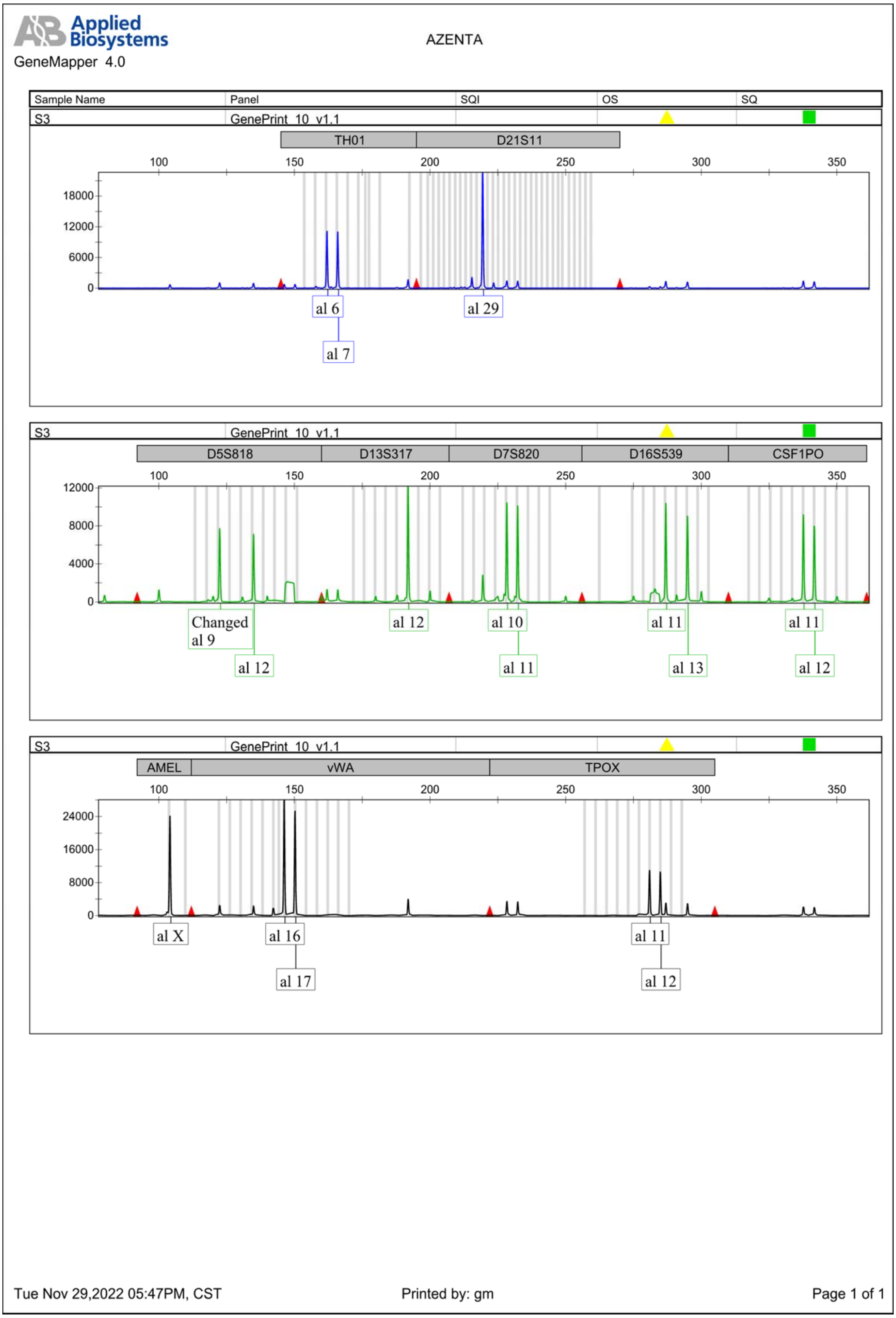

Note：Raw data in appendix

MeisenCTCC

出库质检单

一、产品信息

细胞名称：NCI-H1563

二、检测项目

生长特性：贴壁 ☑ / 悬浮 ☐ / 贴壁+悬浮 ☐

细胞形态：表皮细胞

细胞总量：~ 1×10^6

细胞活力：> 90%

支原体：有 ☐ 无 ☑

三、STR鉴定

Result of STR matching analysis by your data.

DSMZ Profile Database

A graphical presentation is shown at the bottom of this page.

EV	Cell No.	Cell name	D5S818	D13S317	D7S820	D16S539	VWA	TH01	AM	TPOX	CSF1PO	Figures
		Query (Your Cell)	12,13	9,14	7,8	9,13	17,18	6,6	x,y	8,11	10,11	
1.00(36/36)	CRL-5875	NCI-H1563 [H1563]	12,13	9,14	7,8	9,13	17,18	6,6	X,Y	8,11	10,11	-
0.72(26/36)	595	DM-3	12,13	11,11	8,10	9,13	16,17	6,6	X,Y	8,8	10,11	-
0.72(26/36)	IFO50433	KP-N-SI9s	13,13	11,14	8,11	9,13	17,17	6,6	X,Y	8,11	10,10	-
0.67(24/36)	652	WILL-2	9,12	8,9	10,11	9,13	17,18	6,6	X,X	11,11	10,11	-
0.67(24/36)	CRL-2211	CCD-1100Sk	12,13	9,11	7,9	9,11	17,18	6,9.3	X,X	8,10	10,11	-
0.67(24/36)	CRL-3024	HBMEC-3	9,13	9,12	9,11	9,11	15,18	6,6	X,Y	8,11	10,11	-
0.67(24/36)	CRL-7026	Hs 46	12,13	9,12	8,9	9,11	17,20	8,8	X,Y	8,11	10,11	-
0.67(24/36)	CRL-7255	Hs 365.Ct	12,13	8,11	7,10	13,13	14,17	6,6	X,Y	8,11	11,13	-
0.67(24/36)	JCRB1156	Yub631	12,13	10,11	8,10	9,9	14,17	6,6	X,Y	8,12	10,11	-
0.67(24/36)	JCRB3002	Dch1	12,13	11,13	10,12	9,13	17,18	6,9	X,Y	8,11	10,12	-
0.61(22/36)	105	KARPAS-45	11,12	9,14	8,11	9,13	14,18	9,9	X,Y	8,8	11,14	-
0.61(22/36)	247	OCI-AML5	12,13	11,14	11,12	11,13	16,18	7,9.3	X,Y	8,11	10,11	-
0.61(22/36)	398	BD-215	12,13	11,11	7,12	9,13	18,18	8,9	X,Y	10,11	10,11	-
0.61(22/36)	408	GOS-1	11,13	9,13	9,11	9,12	17,18	6,9.3	X,Y	8,9	10,12	-
0.61(22/36)	530	L-1236	11,13	13,13	9,10	9,13	17,18	6,6	X,Y	8,12	10,14	-
0.61(22/36)	CCL-215	CCD-25Lu	12,12	10,14	8,11	9,11	17,18	6,7	X,Y	11,12	10,12	-
0.61(22/36)	CCL-233	SW1116 [SW 1116, SW-1116]	11,12	11,14	12,12	9,12	14,19	6,6	X,Y	8,11	10,11	-
0.61(22/36)	CRL-1474	CCD-25Sk	12,12	10,14	8,11	9,11	17,18	6,7	X,Y	11,12	10,12	-
0.61(22/36)	CRL-2260	HT	11,13	13,14	8,10	11,13	17,18	6,7	X,Y	11,11	10,10	-
0.61(22/36)	CRL-2769	JSC-1 [JHU-32]	12,12	9,14	9,9	11,12	14,18	6,9	X,Y	8,11	10,11	-
0.61(22/36)	CRL-3001	Z-138	11,13	9,12	8,8	11,11	15,18	6,6	X,Y	8,8	10,11	-
0.61(22/36)	CRL-5836	NCI-H226 [H226]	11,12	13,14	8,10	9,12	17,17	8,9.3	X,Y	8,11	10,11	-
0.61(22/36)	CRL-7265	Hs 889(A).Lu	11,11	9,11	8,10	11,13	16,17	6,8	X,Y	8,11	10,11	-
0.61(22/36)	HTB-104	Cates-1B	12,13	12,12	7,12	12,13	16,17	6,6	X,Y	8,8	10,10	-
0.61(22/36)	RCB0697	GM2-1TKB	12,13	9,10	10,12	9,12	17,18	6,6	X,X	8,11	9,12	-
0.61(22/36)	RCB1022	T3M-11	10,13	9,12	8,12	10,13	18,18	6,7	X,Y	8,9	10,11	-
0.61(22/36)	RCB2239	HE19	12,13	11,11	8,10	10,11	17,18	6,9	X,Y	8,8	10,11	-

浙江美森细胞科技有限公司
磐安，浙江， 中国

电话：0571-86027729
邮箱：meisencell@ctcc.online

www.ctcc.online

Applied Biosystems

GeneMapper ID v3.2

210527-1

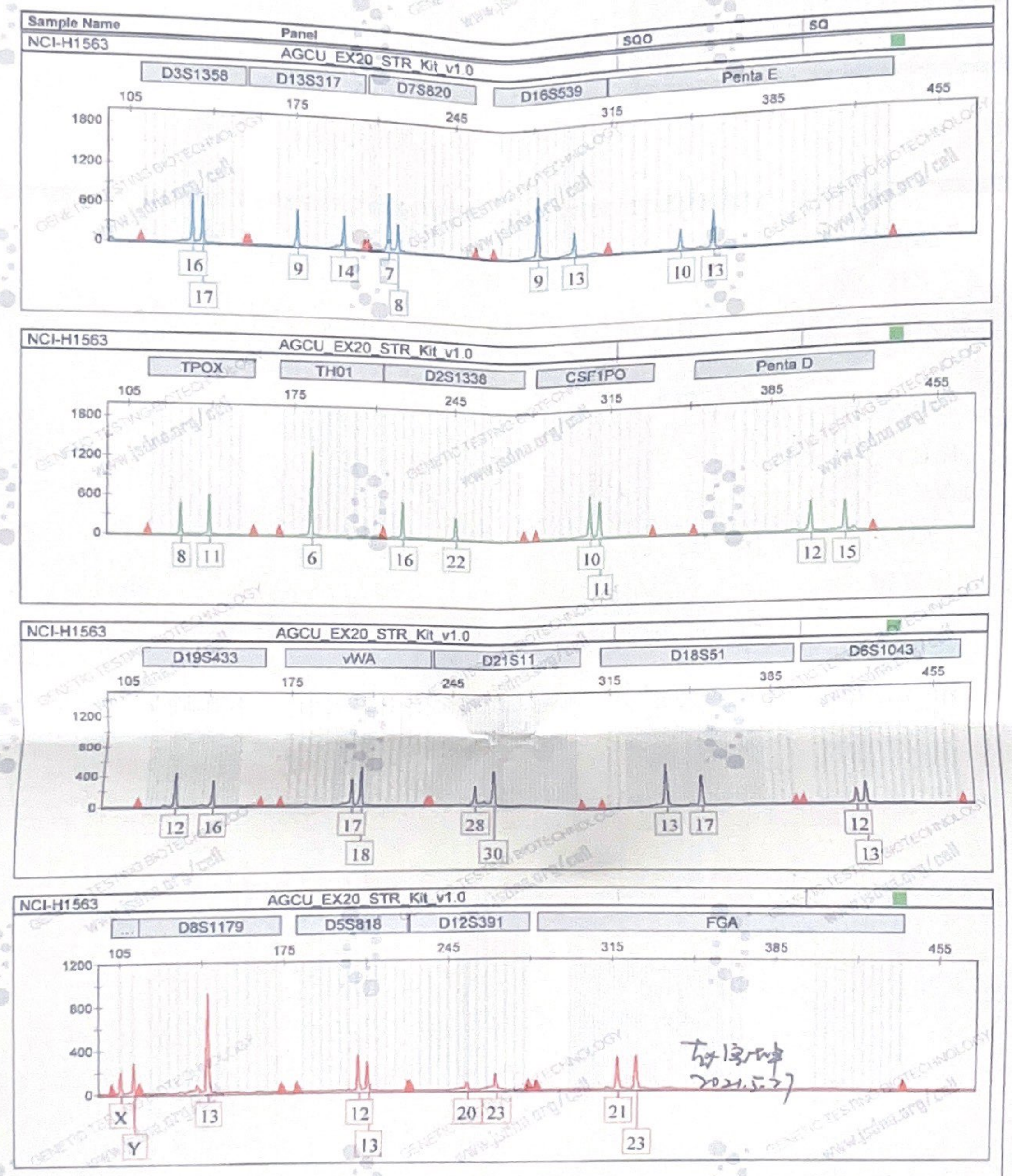

Thu May 27,2021 02:34PM CST　　Printed by: gmid　　Page 1 of 1

浙江美森细胞科技有限公司 质检专用章 33072710017776

浙江美森细胞科技有限公司
磐安, 浙江， 中国

电话: 0571-86027729
邮箱: meisencell@ctcc.online

www.ctcc.online

Meisen CTCC

出库质检单

一、产品信息

细胞名称：HCC2279

二、检测项目

生长特性：贴壁 ☑ / 悬浮 ☐ / 贴壁+悬浮 ☐

细胞形态：上皮细胞

细胞密度：＞75%

细胞总量：~ 1×10^6

细胞活力：＞90%

支原体：有 ☐ 无 ☑

三、STR鉴定

Result of STR matching analysis by your data.

- DSMZ Profile Database -

A graphical presentation is shown at the bottom of this page.

EV	Cell No.	Cell name	Locus names: D5S818	D13S317	D7S820	D16S539	VWA	TH01	AM	TPOX	CSF1PO	Figures
	Query (Your Cell)		10,11	12,12	11,11	11,11	14,17	9,9	X,X	11,11	12,12	
1.00(36/36)	CRL-2870	HCC2279	10,11	12,12	11,11	11,11	14,17	9,9	X,X	11,11	12,12	-
0.78(28/36)	JCRB0746	YKG-1	10,11	8,12	10,11	11,11	16,17	6,9	X,X	11,11	12,12	-
0.78(28/36)	RCB2110	YKG1	10,11	8,12	10,11	11,11	16,17	6,9	X,X	11,11	12,12	-
0.72(26/36)	CRL-5924	NCI-H2110	12,12	12,12	8,11	11,11	15,17	9,9	X,X	8,11	12,12	-
0.67(24/36)	237	IGR-37	11,12	12,12	10,11	11,11	17,21	9,9	X,Y	8,11	11,12	-
0.67(24/36)	649	HCEC-H9C1	11,11	8,11	10,11	11,11	14,17	9,9	X,X	8,8	12,12	-
0.67(24/36)	CRL-1595	C-4 II	9,11	11,12	10,11	11,11	14,14	9,9.3	X,X	10,11	12,12	-
0.67(24/36)	CRL-5922	NCI-H2087 [H2087]	11,11	12,12	8,10	9,11	17,17	7,9	X,X	11,11	12,12	-
0.67(24/36)	CRL-7914	C-4 II	9,11	11,12	10,11	11,11	14,14	9,9.3	X,X	10,11	12,12	-
0.67(24/36)	HTB-94	SW 1353 [SW 1353, SW-1353]	10,11	12,13	9,11	11,12	16,17	6,9	X,X	8,11	12,12	-
0.67(24/36)	IFO50488	OUMS-27	10,11	12,12	10,12	14,14	14,14	9,9	X,X	11,11	10,12	-
0.67(24/36)	JCRB0169	Lu-134-B	11,11	9,9	11,11	9,9	17,17	9,9	X,X	11,11	12,12	-
0.67(24/36)	JCRB0198	OSC-19	10,13	12,12	9,11	9,12	14,14	9,9	X,Y	11,11	12,12	-
0.67(24/36)	JCRB0235	Lu-134-A-H	11,11	9,9	11,11	9,9	17,17	9,9	X,X	11,11	12,12	-
0.67(24/36)	JCRB1074	FU97	10,12	10,12	11,11	9,11	17,17	9,9	X,X	12,12	12,12	-
0.67(24/36)	RCB0466	Lu-134-A	11,11	9,9	11,11	9,9	17,17	9,9	X,X	11,11	12,12	-
0.67(24/36)	RCB0467	Lu-134-B	11,11	9,9	11,11	9,9	17,17	9,9	X,X	11,11	12,12	-
0.67(24/36)	RCB1316	CTB-1	11,13	8,9	11,11	11,12	16,17	9,9	X,X	8,11	12,12	-
0.65(24/37)	239	IGR-39	11,12	12,12	10,11	11,11	17,21	9,9	X,Y	8,11	11,12,11	-
0.61(22/36)	41	LP-1	11,11	12,12	11,12	11,12	17,17	7,8	X,X	11,11	11,12	-
0.61(22/36)	47	DOHH-2	11,12	12,13	11,11	11,14	14,19	9,9	X,Y	8,11	11,12	-
0.61(22/36)	206	COLO-720L	10,11	8,11	10,10	11,11	14,14	9,9	X,X	11,11	11,13	-
0.61(22/36)	279	BHT-101	10,11	12,12	10,11	9,11	19,19	9,9.3	X,X	8,8	12,12	-
0.61(22/36)	524	DERL-7	10,11	11,12	11,12	10,11	17,19	9,9	X,Y	8,11	11,12	-

浙江美森细胞科技有限公司
磐安, 浙江， 中国

电话: 0571-86027729
邮箱: supports@ctcc.org

www.ctcc.org

Applied Biosystems

210413

GeneMapper ID v3.2

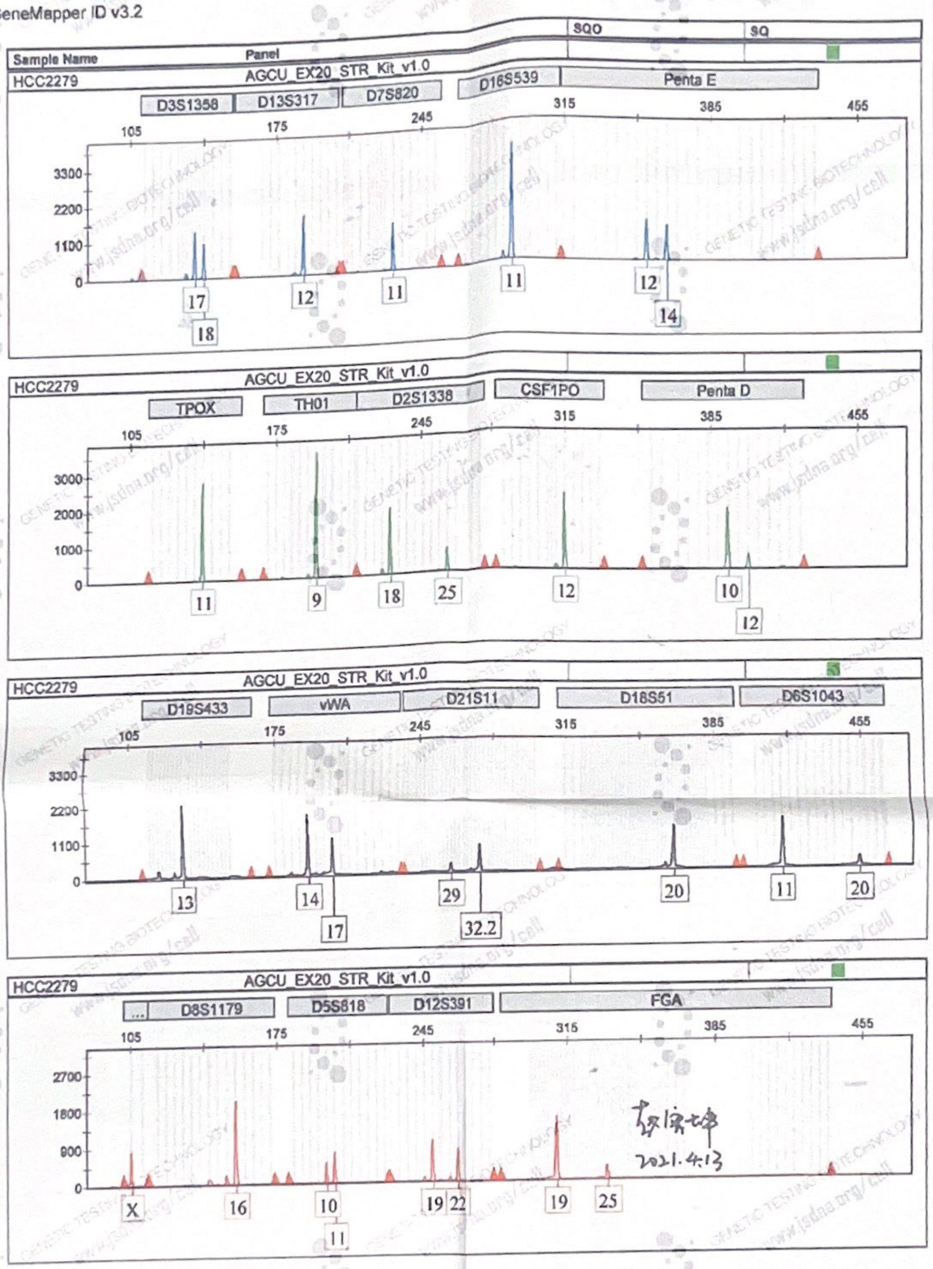

Tue Apr 13,2021 02:54PM, CST

Printed by: gmid

Page 1 of 1

浙江美森细胞科技有限公司
磐安, 浙江, 中国

www.ctcc.org

电话: 0571-86027729
邮箱: supports@ctcc.org

Cell Line Authentication Service

STR Profiling Report

Sample Code: HCC4006

Sample Type: Cell Line

Testing Method: STR Genotyping

Report Time: April 15, 2021

COMPANY STATEMENT

1. THIS REPORT IS ONLY RESPONSIBLE FOR THE SAMPLES ANALYZED.
2. THE TESTING RESULTS AND THE ORGANIZATION NAME WILL NOT BE USED FOR ADVERTISEMENT, COMMERCIAL EXHIBITIONS, COMMERCIAL PERFORMANCE AND OTHER COMMERCIAL ACTIVITIES.
3. OBJECTIONS SHOULD BE RAISED WITHIN FIFTEEN DAYS AFTER THE RECEIPT OF THIS REPORT.
4. THE PAPER REPORT WITH CONTENT ALTERING, ADDING ARE INVALID.

Testing Company: Shanghai Biowing Applied Biotechnology Co. Ltd
Address: Room 502, NO.1015 Longteng Rd , Songjiang District, Shanghai
Tel: +86-021-33559491
Contact: Wenyao Zhang
E-mail: market@biowing.com.cn

Cell Line Authentication – STR Profiling Report

Sample code

Table 1. Sample Code

Customer's code	Company Code
HCC4006	20210410-01

Sample Number :1

Sample Type: Cell line

Testing Type: STR

Testing Method:

DNA was extracted by a commercial kit from CORNING (AP-EMN-BL-GDNA-250G). The twenty STRs including Amelogenin locus were amplified by six multiplex PCR and separated on ABI 3730XL Genetic Analyzer. The signals were then analyzed by the software GeneMapper.

Data Interpretation:

Cell lines were authenticated using Short Tandem Repeat (STR) analysis asdescribed in 2012 in ANSI Standard (ASN-0002) by the ATCC Standards Development Organization (SDO) and in Capes-Davis et al., Match criteria for human cell line authentication: Where do we draw the line? Int J Cancer.2013;132(11):2510-9.

Test Results

1. STR profile

Table 2. STR and Amelogenin Genotyping Results of Cell line.

Loci	**Sample information**			**Cell Bank information**		
	Sample name: HCC4006			Cell line name: HCC4006		
	**Allele1**	**Allele2**	**Allele3**	**Allele1**	**Allele2**	**Allele3**
D5S818	12	12		12	12	
D13S317	11	12		11	12	
D7S820	9	12		9	12	
D16S539	11	12		11	12	
VWA	16	17		16	17	
TH01	7	7		7	7	
AMEL	X	X		X	X	
TPOX	8	9		8	9	
CSF1PO	10	10		10	10	
D12S391	21	22				
FGA	21	22	23			
D2S1338	17	24				
D21S11	31	31				
D18S51	15	19				
D8S1179	10	14				
D3S1358	16	18				
D6S1043	17	17				
PENTAE	7	13				
D19S433	12	13				
PENTAD	9	14				

2. database annotation

Figure 1. STR matching analysis

EV	Cell No.	Cell name	Locus names								
			D5S818	D13S317	D7S820	D16S539	VWA	TH01	AM	TPOX	CSF1PO
	Query (Your Cell)		*12,12*	*11,12*	*9,12*	*11,12*	*16,17*	*7,7*	*X,X*	*8,9*	*10,10*
1.00(36/36)	CRL-2871	HCC4006	12,12	11,12	9,12	11,12	16,17	7,7	X,X	8,9	10,10

Note: The STR online match analysis of the test cell against DSMZ database, showing cell number (Cell No.) and cell name.

3. Authentication

☐ The submitted sample profile is human, but not a match for any profile in the DSMZ STR database.

☑ The submitted profile is exact match for the following human cell line(s) in the DSMZ STR database (8 core loci plus Amelogenin): **HCC4006**.

☐ The submitted profile is similar to the following DSMZ human cell line: /.

- **Note:** A cell line can be considered to be authenticated when 80% (exact match) of the alleles in its STR profile match profiles from tissue or other cell line samples from that donor or from database. Cell lines with between a 55% to 80% (similar) match require further profiling for investigation of relatedness.

Appendix

1. Genotyping Strategy and Site Distribution

Table S1. Experimental Strategy and Sites

	**Strategy 1**	**Strategy 2**	**Strategy 3**	**Strategy 4**
1	D3S1358	D8S1179	D19S433	AMEL
2	VWA	D21S11	TH01	D6S1043
3	D7S820	D16S539	D13S317	D5S818
4	CSF1PO	D2S1338	TPOX	D12S391
5	PENTAE	PENTAD	D18S51	FGA

The allele match algorithm compares the 8 core loci plus amelogenin only, even though alleles from all lociwill be reported when available.

2. DSMZ tools was used to carry on the cell line comparison, which contains 2455 cell lines STR data from ATCC, DSMZ, JCRB ,ECACC， GNE and RIKEN databases. If the cell is not included in the above cell library, users need to compared with other databases.

Technician: Jianan Zhang

Checked by: Ning Qian

Issued by: Yang Bai

Issue date: April 15, 2021

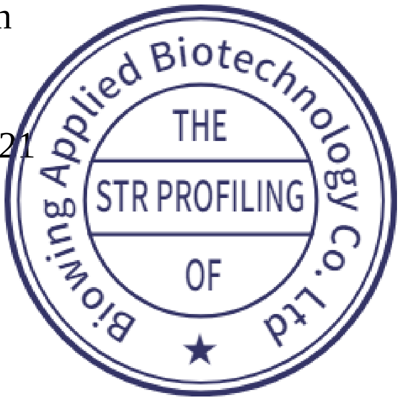

NCI-H1299 细胞 STR 鉴定报告

一、 材料处理和检验方法

取适量 **NCI-H1299** 细胞(编号 XB0421-1757,1×10^{6})使用 Microread Genomic DNA Kit 提取 DNA，采用 Microreader^{TM}21 ID System 扩增 20 个 STR 位点和性别鉴定位点，使用 ABI 3130x1 型遗传分析仪进行 PCR 产物检测，使用 GeneMapperID-X 软件（Applied Biosystems）对检测结果进行分析，并与 ATCC、DSMZ、Cellosaurus 等数据库进行比对。

二、 检测结果

实验中阴性及阳性对照结果均正确。

NCI-H1299 细胞株的 STR 位点和 Amelogenin 位点的基因分型结果见附表，分型图谱见附图。

三、 分析说明

NCI-H1299 细胞株基因组 DNA 扩增后图谱清晰，分型结果良好。

四、 检验结论

1. **NCI-H1299** 细胞株 DNA 进行细胞 STR 分型结果显示，细胞株中未发现人类细胞交叉污染。
2. 该细胞株 DNA 分型在细胞库中找到与其细胞分型 98.04%相匹配的细胞株，细胞株名称为 **NCI-H1299**。

www.procell.com.cn

附表 1：细胞株 NCI-H1299 的 STR 位点和 Amelogenin 位点的基因分型结果

STR Loci	样品名称：XB0421-1757	数据库名称：NCI-H1299
Amelogenin	X	X
CSF1PO	12	12
D2S1338	23,24	23,24
D3S1358	17	17
D5S818	11	11
D7S820	10	10
D8S1179	10,13	10,13
D13S317	12	12
D16S539	12,13	12,13
D18S51	16	16
D19S433	14	14
D21S11	32.2	32.2
FGA	20	20
PentaD	13	13
PentaE	11	11
TH01	6,9.3	6,9.3
TPOX	8	8
vWA	16,18	16,18
D6S1043	11,13	
D12S391	21,22	21
D2S441	11,13	11,13
Cellosaurus 数据库匹配度 98.04%，匹配位点数 19（https://web.expasy.org/cellosaurus-str-search/）		

备注：

1. 根据国际细胞鉴定委员会(ICLAC)制定的细胞 STR 鉴定标准，细胞系的匹配度≥80% 时，认为它们具有相关性，即衍生于共同的祖先细胞；匹配度在 55% 至 80% 之间，需要进一步验证相关性；小于 55%，表明两者不具有相关性。
2. 图谱有效峰为真实的 PCR 条带;小峰和非特异性条带在计算中忽略不计。
3. STR 数据比对结果默认 ExPASy，数据来源包括 ATCC，DSMZ，JCRB 等细胞库以及文献和资料记载，数据库入口 https://web.expasy.org/cellosaurus-str-search/。

Copyright ©2019-2020 Procell Life Science&Technology Co.,Ltd. All Rights Reserved

附图 1：NCI-H1299 细胞 STR 位点和 Amelogenin 位点的基因分型结果

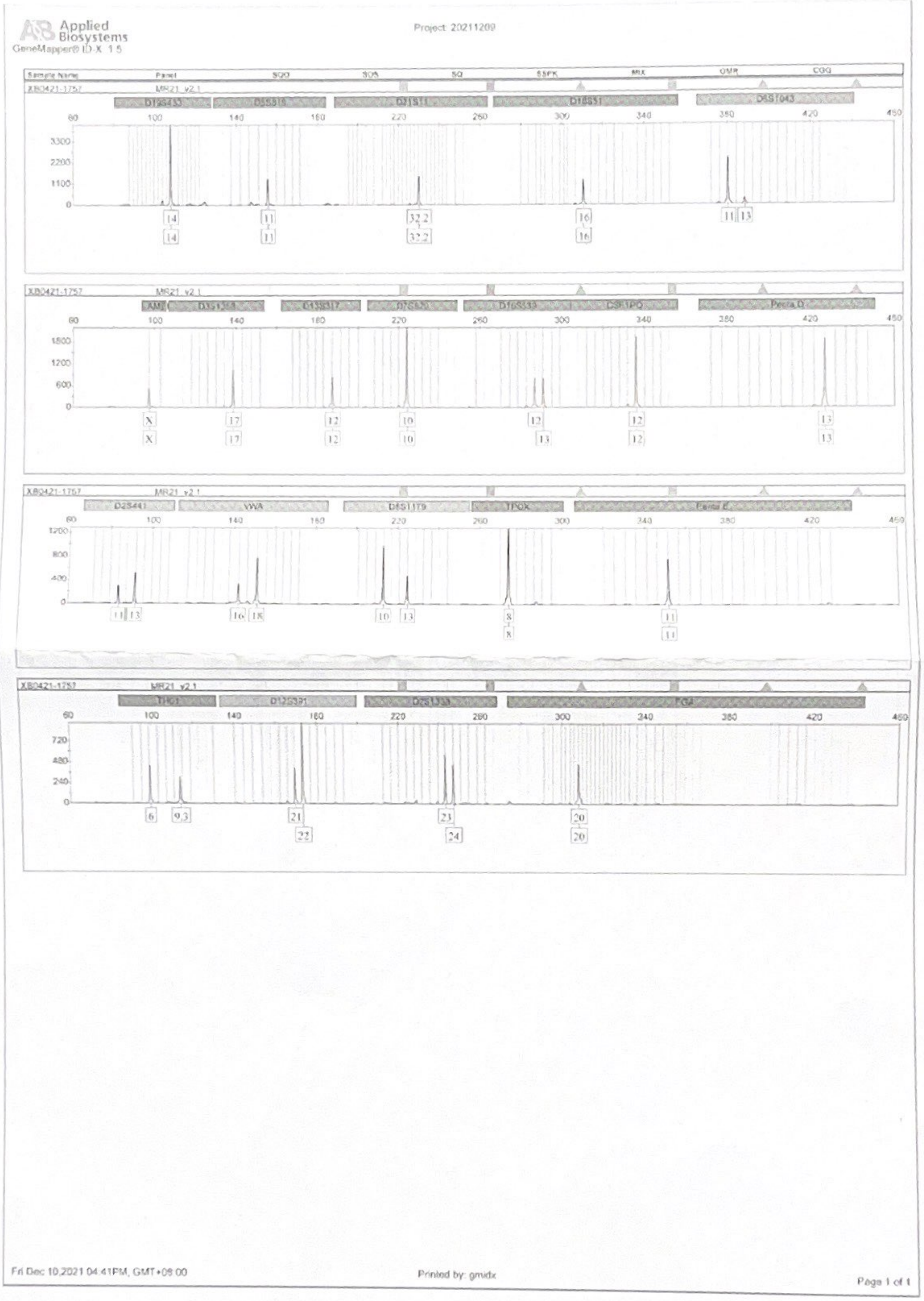

NCI-H1975 细胞 STR 鉴定报告

一、 材料处理和检验方法

取适量 **NCI-H1975** 细胞(编号 PC-H2022072501,1×10^6)使用 Microread Genomic DNA Kit 提 取 DNA，采用 Microreader™21 ID System 扩增 20 个 STR 位点和性别鉴定位点，使用 ABI 3730xl 型遗 传分析仪进行 PCR 产物检测，使用 GeneMapper Software6 软件（Applied Biosystems）对检测结果进行分析，并与 ATCC、DSMZ、JCRB、ExPASy 等数据库进行比对。

二、 检测结果

实验中阴性及阳性对照结果均正确。

NCI-H1975 细胞株的 STR 位点和 Amelogenin 位点的基因分型结果见附表，分型图谱见附图。

三、 分析说明

NCI-H1975 细胞株基因组 DNA 扩增后图谱清晰，分型结果良好。

四、 检验结论

1. **NCI-H1975** 细胞株 DNA 进行细胞 STR 分型结果显示，细胞株中未发现人类细胞交叉污染。
2. 该细胞株 DNA 分型在细胞库中找到与其细胞分型 98.31%相匹配的细胞株，细胞株名称为 **NCI-H1975**。

普诺赛® | Pricella Procell

网站：www.procell.com.cn
电话：400-999-2100；027-87287608
邮箱：sales@procell.com.cn
地址：湖北省武汉市高新大道858号生物医药产业园三期C4栋

普诺赛® | Pricella
Procell
武汉普诺赛生命科技有限公司
Procell Life Science&Technology Co.,Ltd.

附表 1：细胞株 NCI-H1975 的 STR 位点和 Amelogenin 位点的基因分型结果

STR Loci	样品名称：PC-H2022072501	数据库名称：[illegible]
Amelogenin	X	X
CSF1PO	12	12
D2S1338	17	17
D3S1358	14,15	14,15
D5S818	11,12	11,12
D7S820	8	8
D8S1179	13,16	13,16
D13S317	10,13	10,13
D16S539	9,12	9,12
D18S51	13	13
D19S433	15,15.2	15,15.2
D21S11	28	28
FGA	21,24	21,24
PentaD	12,13	12,13
PentaE	12,16	12,16
TH01	7	7
TPOX	8,11	8,11
vWA	18	18
D6S1043	12	
D12S391	17	17
D2S441	11	10,11
ExPASy 数据库匹配度 98.31%，匹配位点数 19（https://web.expasy.org/cellosaurus-str-search/）		

备注：

1. 根据国际细胞鉴定委员会(ICLAC)制定的细胞 STR 鉴定标准，细胞系的匹配度≥80% 时，认为它们具有相关性，即衍生于共同的祖先细胞；匹配度在 55% 至 80% 之间，需要进一步验证相关性；小于 55%，表明两者不具有相关性。
2. 图谱有效峰为真实的 PCR 条带;小峰和非特异性条带在计算中忽略不计。
3. STR 数据比对结果默认 ExPASy，数据来源包括 ATCC，DSMZ，JCRB 等细胞库以及文献和资料记载，数据库入口 https://web.expasy.org/cellosaurus-str-search/。

网站：www.procell.com.cn
电话：400-999-2100；027-87287608
邮箱：sales@procell.com.cn
地址：湖北省武汉市高新大道858号生物医药产业园三期C4栋

普诺赛® | Pricella
Procell
武汉普诺赛生命科技有限公司
Procell Life Science&Technology Co.,Ltd.

附表 1：NCI-H1975 细胞 STR 位点和 Amelogenin 位点的基因分型结果。

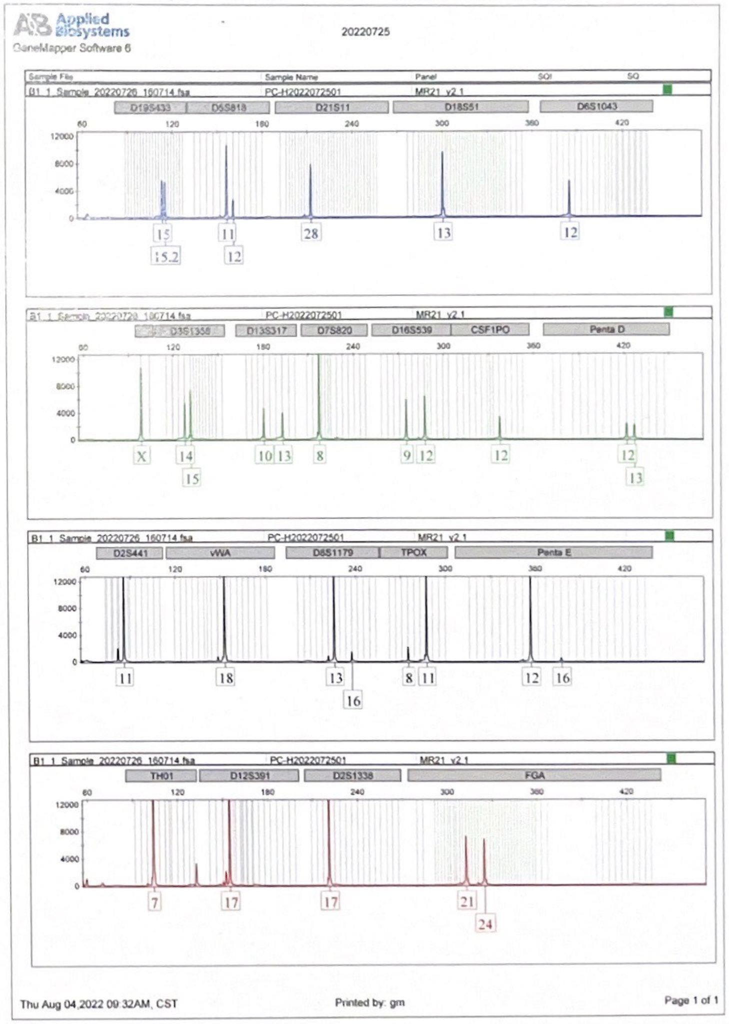

网站：www.procell.com.cn
电话：400-999-2100；027-87287608
邮箱：sales@procell.com.cn
地址：湖北省武汉市高新大道858号生物医药产业园三期C4栋
