# Supplementary material for: A cohort-based multi-omics identifies nuclear translocation of eIF5B /PD-L1/CD44 complex as the target to overcome Osimertinib resistance of ARID1A-deficient lung adenocarcinoma
Source: Exp Hematol Oncol. 2025 Jan 7;14:3. doi: 10.1186/s40164-024-00594-4 (PMC11705878; doi:10.1186/s40164-024-00594-4)
Supplement: Supplementary file 1 — Additional file 1. [file 40164_2024_594_MOESM1_ESM.zip › New folder/Supplementary materials and methods.docx]

**Supplementary materials and methods**

1. **Bioinformatic analyses**

We utilized the cBioPortal for Cancer Genomics (cBioPortal) database [1-2] to examine the mutation frequency of SWI/SNF family member genes in two pancancer datasets: China-pan-cancer (OrigiMed, Nature 2022) and Pan-cancer analysis of whole genomes (ICGC/TCGA, Nature 2020). The Investigating Genetic Model of Drug Response (iGMDR) database [3] was utilized in the current study to investigate the genetic model of drug response and identify potential targets and associated signaling pathways related to ARID1A in tumors. The enrichment analysis was performed using data obtained from The Cancer Genome Atlas (TCGA) database with the aim of predicting potential pathways in lung adenocarcinoma (LUAD) patients grouped based on ARID1A expression. Furthermore, the Gene Expression Profiling Interactive Analysis (GEPIA) database [4] was utilized to evaluate prognosis based on the expression levels of different molecules. The Kaplan‒Meier method was employed to conduct survival analysis via the SangerBox online platform [5]. In addition, correlations between the expression of various genes were assessed using the Tumor Immune Estimation Resource 2.0 (TIMER 2.0) database [6]. In addition, we used the NucleOlar localization sequence Detector (NoD) database [7-8] to explore the nucleolar localization sequences (NoLSs) in the targeted proteins.

1. **Patient selection and follow-up**

The current study involved 101 patients diagnosed with LUAD harboring sensitizing EGFR mutations (19del or 21L858R) who were admitted to our cancer center between August 2012 and September 2021 and received EGFR-tyrosine kinase inhibotor (EGFR-TKI) treatment. Comprehensive data from 77 patients who received Osimertinib treatment, which included the patients' demographic information, EGFR mutation profile, T790M status, and treatment history, were collected (as shown in **Table S1**). All patients were diagnosed with stage IV LUAD according to hematoxylin and eosin (H&E) staining and immunohistochemical (IHC) staining in the pathology department in our hospital, and the disease status was evaluated using the Response Evaluation Criteria in Solid Tumors 1.1 (RECIST 1.1) standard. Tumor staging was performed in accordance with the 2019 American Joint Committee on Cancer (AJCC) TNM staging system for lung cancer [9]. Ethical approval for the study was obtained from the Ethics Committee of the Cancer Hospital, Chinese Academy of Medical Sciences (No. NCC-007421), and all experiments were conducted according to the tenets of the Declaration of Helsinki and the guidelines of the National Health and Family Planning Commission of the Professional Regulation Commission (PRC). The last follow-up for all patients was in March 2023.

1. **IHC assay for protein expression**

The IHC slides utilized to assess ARID1A, MDM2 and PD-L1 expression were obtained with the explicit consent of all enrolled patients. IHC analysis was conducted on five-micrometer-thick sections of formalin-fixed, paraffin-embedded (FFPE) tissues. Antigen retrieval was achieved by boiling the slides in 10 mM citrate buffer (pH 9.0) for 10 min, followed by cooling at room temperature for 20 min. Each section was incubated with a primary antibody against ARID1A (1:500), MDM2 (1:100) or PD-L1 (1:100) at the appropriate concentration overnight at 4 °C. The IHC slides were independently reviewed by two investigators, and five randomly selected fields of view on each slide were evaluated for IHC scoring with a previously reported scoring method [10-13]. The staining intensity for ARID1A and MDM2 was scored as 0 (no staining), 1 (weak staining), 2 (medium staining), or 3 (strong staining), while the percentile score were assigned as 0 (< 5%), 1 (5-25%), 2 (26-50%), 3 (51-75%), or 4 (76-100%). The final score of each slide was calculated as the average score of the 5 fields and ranged from 0 to 12 (intensity score x percentage score). Based on the selection of the best cutoff value for the prognosis of Osimertinib treatment by SangerBox, the best cutoff value for ARID1A expression in Osimertinib-treated patients was defined as a final score greater than 4, while the best cutoff value for MDM2 was defined as a final score greater than 6. We analyzed the relationship between ARID1A expression levels and occurrences of LUAD metastasis, as well as resistance to first- and third-generation EGFR-TKI, drawing on insights from our previously published studies [11-12] alongside the findings of the current investigation. This relationship is illustrated through a numerical axis diagram to provide guidance for clinical treatment. The tumor proportion score (TPS) was used for evaluation of PD-L1 expression, and nuclear staining of PD-L1 was identified. The patients who were enrolled in the study were categorized according to the ARID1A IHC score (threshold: 9) to assess the proportions of positive nuclear PD-L1 (nPD-L1) staining. Further information regarding the antibodies used is provided in **Table S2**.

1. **Proteomic analysis based on clinical samples**

In this study, 101 samples (FFPE sections) were selected for proteomic analysis by Shanghai iProteome Biotechnology Co., Ltd. (Shanghai, China). These enrolled samples included 68 treatment-naive samples, with 55 samples collected before 1G EGFR-TKI treatment and 13 samples collected before first-line Osimertinib treatment. Additionally, 33 post-treatment samples, specifically, 22 samples collected after 1G EGFR-TKI treatment and 11 samples collected after Osimertinib treatment, were included.

**4.1 Sample preparation**

Sample preparation was performed with the FFomic strategy. Accurate evaluation of tumor cellularity was performed using the middle section of each tumor tissue block, which was resected and subjected to H&E staining. For preparation of samples for proteomic and phosphoproteomic analyses, sections (5 μm thick) were sliced, deparaffinized with xylene, and washed in an ethanol gradient. Specimens selected according to H&E staining were scraped using a dissecting microscope and were then stored at − 80 °C until needed.

**4.2 Protein extraction from FFPE tissues and trypsin digestion**

Samples were lysed in TCEP buffer (2% deoxycholic acid sodium salt, 40 mM 2-chloroacetamide, 100 mM Tris–HCl, 10 mM Tris(2-chloroethyl) phosphate, 1 mM PFSM; pH 8.5) supplemented with protease and phosphatase inhibitors at 99 °C for 30 min. After cooling to room temperature, trypsin was added, and the tissues were digested for 18 h at 37 °C. Then, 10% formic acid was added, and the samples were vortexed for 3 min and precipitated by centrifugation for 5 min (12,000 × g). Next, a new 1.5-mL tube containing extraction buffer (0.1% formic acid in 50% acetonitrile) was used to extract the supernatant (vortexing for 3 min followed by precipitation by centrifugation at 12,000 × g for 5 min). The collected supernatant was transferred into a new tube and dried using a SpeedVac.

**4.3 Nano-liquid chromatography–tandem mass spectrometry (nano-LC–MS/MS) analysis**

Peptides in the proteome profiling samples were analyzed on an Orbitrap Fusion Lumos Hybrid Quadrupole-Orbitrap mass spectrometer (Thermo Fisher Scientific) coupled to a high-performance liquid chromatography system (EASY nLC 1200, Thermo Fisher Scientific). Dried peptide samples redissolved in solvent A (0.1% formic acid in water) were loaded onto a 2-cm in-house-packed trap column (100 μm inner diameter, 3 μm ReproSil-Pur C18-AQ beads, Dr. Maisch GmbH) in solvent A and separated on a 150 μm-inner-diameter column with a length of 30 cm (1.9 μm ReproSil-Pur C18-AQ beads, Dr. Maisch GmbH) over a 150 min gradient (solvent A: 0.1% formic acid in water; solvent B: 0.1% formic acid in 80% acetonitrile (can)) at a constant flow rate of 600 nl/min (0-150 min, 0 min, 4% B; 0-10 min, 4%-15% B; 10-125 min, 15%-30% B; 125-140 min, 30%-50% B; 140-141 min, 50%-100% B; 141-150 min, 100% B). Eluted peptides were ionized at 2 kV and injected into the mass spectrometer. MS was performed in data-dependent acquisition (DDA) mode. For the MS1 spectrum full scan, ions with an m/z ranging from 300 to 1,400 were acquired in an Orbitrap mass analyzer at a high resolution of 120,000. The automatic gain control (AGC) target value was set to 3×10^6^. The maximal ion injection time was 80 ms. MS2 spectral acquisition was performed in an ion trap in rapid speed mode with a 1.5 s cycle time. Precursor ions were selected and fragmented by higher energy collision dissociation (HCD) with a normalized collision energy of 30%. Fragment ions were analyzed by an ion trap mass analyzer with the AGC target value set to 5E+04. The maximal ion injection time for the MS2 scans was 20 ms. Peptides that triggered MS/MS scans were dynamically excluded from further MS/MS scans for 18 s. The coefficient of variation (CV) values for field asymmetric waveform ion mobility spectrometry (FAIMS) were -45 V and -65 V.

**4.4 Data analysis**

The original MS data were in RAW files, and the iProteome one-stop data analysis cloud platform was used for qualitative and quantitative analysis.

**4.5 Spatial proteomic analysis**

In this research, we selected 8 samples with positive nPD-L1 expression. These samples were then sent to Westlake Omics (Hangzhou, China) for spatial proteomic analysis. The methodology for this analysis was previously described in a study [14].

1. **10x Genomics spatial transcriptomics based on clinical samples**

**5.1 Sample preparation and RNA quality assessment**

The FFPE sections were stored at 4 °C and were protected from exposure to direct light to ensure even chilling and to preserve RNA integrity. The RNA quality of the FFPE tissues was assessed before proceeding with sectioning by calculating the percentage of total RNA fragments containing >200 nucleotides (DV200) among the RNA extracted from the FFPE tissue sections.

**5.2 Deparaffinization, H&E staining and decrosslinking**

Tissues were sectioned as described in the Visium CytAssist Spatial Gene Expression for FFPE protocol. Five-micrometer sections were placed on a Superfrost™ Plus Microscope Slide (Fisherbrand™) and stained with H&E following deparaffinization. The sections were imaged and subjected to decrosslinking. The glass slides with tissue sections were processed with a Visium CytAssist instrument to transfer analytes to a Visium CytAssist Spatial Gene Expression slide. The probe extension and library construction steps followed the standard Visium CytAssist Spatial Gene Expression workflow outside the instrument.

**5.3 Probe hybridization, RNA digestion and probe release**

The whole-transcriptome probe panel, consisting of a pair of specific probes for each targeted gene, was added to the deparaffinized, stained, and decrosslinked tissues. Together, the probe pairs hybridized to their complementary target RNA. After hybridization, a ligase was added to seal the junction between the probe pairs that have hybridized to RNA molecules, forming a ligation product. The single-stranded ligation products were released from the tissue upon RNase treatment and permeabilization and were then captured on Visium slides.

**5.4 Reverse transcription**

Probes were extended by the addition of a unique molecular identifier (UMI), spatial barcode and a partial read 1 sequence. RT Master Mix containing reverse transcription reagents was added to the permeabilized tissue sections. By this process, spatially barcoded, ligated probe products were generated, and these products were then carried forward for library preparation.

**5.5 Construction of the gene expression library and sequencing**

The spatially barcoded, ligated probe products were released from the slide and harvested for quantitative PCR (qPCR) to determine the Sample Index PCR cycle number. The products were then indexed via Sample Index PCR. The final libraries contained the P5 and P7 primers used for amplification on the Illumina platform. Library molecules were cleaned with SPRIselect, assessed on a bioanalyzer or a similar instrument, quantified, and then sequenced. Sequencing was carried out in PE150 mode on the Illumina NovaSeq platform in Berry Genomics Corporation (Beijing, China).

1. **Reagents**

Inhibitors, including Lorlatinib (S7536), Osimertinib (S7297), GSK-126 (S7061), Simvastatin (S1792), Crizotinib (S1068), BMS-1166 (S8859), Fraxinellone (S9100), Amlodipine (S1905), MG-132 (S2619), Importazole (S8446), Rapamycin (S1039) and 3-Methyladenine (3-MA, S2767), were procured from Selleck. ARB-272572 (HY-142221) was purchased from MedChemExpress (MCE). These inhibitors were dissolved in dimethyl sulfoxide (DMSO) to make stock solutions, which were further diluted to suitable concentrations as required for experimental purposes. The inhibitor solutions were preserved in a -80 °C freezer.

1. **Culture and stable transduction of cell lines**

ARID1A wild-type cell lines, including the A549 (KRAS mutation), NCI-H1299 (wild type), NCI-H1975 (EGFR 21L858R/20T790M) and HCC4006 (EGFR 19 del) LUAD cell lines, the AGS gastric cancer cell line, and ARID1A mutant cell lines, including the HCC2279 (EGFR 19 del) and NCI-H1563 cell lines, were purchased from the Cell Bank of the Chinese Academy of Sciences (Shanghai, China), Procell Life Science & Technology Co. (Wuhan, China) and Meisen Chinese Tissue Culture Collections (Zhejiang, China). Information regarding the short tandem repeat (STR) Cell ID assays is provided in **Supplementary file 1.** All LUAD cell lines were cultured in RPMI-1640 medium (F12K medium for the AGS cell line) supplemented with 10% fetal bovine serum (FBS) and 1% penicillin/streptomycin (P/S; 100 IU/mL penicillin and 100 IU/mL streptomycin) in a 37 °C humidified atmosphere with 5% CO_2_. Lentiviral vectors encoding short hairpin RNAs (shRNAs) for ARID1A, MDM2 and PD-L1, single-guide RNAs (sgRNAs) for eIF5B and ARID1A, lentiviral vectors containing the ARID1A and PD-L1 coding sequences, and the corresponding vector controls (negative control [NC] vectors) were purchased from GeneChem (Shanghai, China). Using helper solution (GeneChem, Shanghai, China), cells were infected with lentiviruses according to the manufacturer’s instructions, after which the infection efficiency was verified by fluorescence microscopy. As a preliminary evaluation of the infection efficiency, cell counting revealed that more than 90% of the cells expressed fluorescent protein, which was considered an appropriate expression efficiency. We established Osimertinib-resistant (OR) cells by sustained exposure to a low-dose drug (0.5 µM) for over six months. The increase in IC50 for Osimertinib (24 h) was measured using an MTS assay, and a more than twofold increase was defined as relative resistance. For subsequent culture, we maintained the cells in 0.5 µM Osimertinib, and prior to conducting phenotypic experiments, we reverted the medium to standard culture conditions 72 hours before the assays. Subsequently, proteins expression was examined using Western blotting (WB). Stably infected cell lines were selected by culture for seven days with 2 μg/mL puromycin (Solarbio, Beijing, China) [6, 8]. The sh-ARID1A, sh-MDM2, sh-PD-L1, sg-eIF5B, sg-ARID1A and corresponding vector control sequences are listed in **Table S2**.

1. **Generating LUAD cell lines with integrated tet-inducible PTEN expression**

LUAD cell lines, including parental cells and ARID1A knockdown (ARID1A_kd) cells, were routinely cultured and prepared for infection with lentiviruses containing the Tet-on system and modified rt-TA protein (GeneChem, Shanghai, China) using helper solution. Following the establishment of LUAD cell lines with tet-inducible PTEN expression, doxycycline (GeneChem, Shanghai, China) was utilized to initiate the expression of PTEN. To this end, various concentrations of doxycycline were employed, and a final concentration of 1 µg/mL was used for cell treatment and evaluation of protein expression levels.

1. **3D cell culture**

3D cell culture was carried out using Biozellen®3D cell culture matrix. A 24-well culture plate was placed on ice for 30 min. A 1:1 mixture of 2×10^5^ to 2×10^7^ cells in 0.5 ml of 37 °C cell culture medium and 0.5 mL of 37 °C extracellular matrix gel was prepared, resulting in a final cell density of 1×10^5^ ~1×10^7^ cells/mL. Next, 20-40 μL of the mixture was pipetted onto the prechilled culture plate and allowed to solidify for approximately 5 min. After solidification, 1 mL of chilled buffer solution was added, and the plate was incubated for 15 min. The buffer solution was carefully removed, and a suitable culture medium was added for cell growth. The gel containing cells was then incubated in a 37 °C carbon dioxide incubator for 4 days allowing observation of the formation of cell spheres.

1. **Colony formation assay**

For the colony formation assay, six-well plates were seeded with 3,000 cells per well. Following incubation of the cells for 14 days, colonies containing over 50 cells were counted. The cells were fixed with paraformaldehyde for 15 min and stained with crystal violet solution for 30 min. Using a light microscope, images were acquired, and the numbers of colonies on the plates were determined.

1. **IncuCyte real-time dynamic cell imaging**

Cells (5,000 cells per well) were seeded into 96-well plates and incubated overnight in the IncuCyte system. The following day, varying concentrations of Osimertinib ranging from 0 µM to 100 µM were added to the medium. The cells were then monitored in the IncuCyte system for three days, and proliferation curves for each concentration of Osimertinib were generated. Additionally, dynamic images of the cells were acquired by the system.

1. **Wound healing assay**

To assess the migratory potential of the cells, a wound healing assay was executed. Scratches were made in the surface of cells in 6-well plates using a 20 µL pipette tip. The wounded regions were imaged under a light microscope at 0 h, 24 h, and 72 h post-incubation with FBS-free medium. The wound healing assay results are expressed as ratios calculated based on the difference in the scratch area in the treatment group compared with that in the vector control group at 72 h [7].

1. **Transwell cell migration and invasion assays**

To assess cell migration and invasion, we employed Transwell compartments with eight-micrometer pore membranes obtained from Corning (NY). Specifically, for the migration assay, we seeded 6×10^4^ cells into the upper compartment in serum-free medium containing 0.5 µM or 1 µM Osimertinib and incubated them for 48 h in a 37 °C humidified atmosphere containing 5% CO_2_. For the invasion assay, we added Matrigel (BD Biosciences, San Jose, CA) to each well, seeded 15×10^4^ cells in medium containing 0.5 µM or 1 µM Osimertinib in each well, and incubated the plate for 72 h. Following incubation, formalin solution (Solarbio, 10% neutral buffered formalin) was used to fix the migrated cells in the lower chamber of each Transwell compartment, and these cells were then stained for 20 min at room temperature with 0.5% crystal violet. Subsequently, we calculated the average number of migrated cells in five random fields of view (at 40× magnification) using an upright fluorescence microscope from Nikon (Tokyo, Japan).

1. **RNA sequencing (RNA-seq) library construction, sequencing and data analysis**

RNA-seq analysis and high-throughput sequencing and data analysis were conducted by Seqhealth Technology Co., Ltd. (Wuhan, China). Total RNA was extracted from cultured cells using TRIzol Reagent (Invitrogen, 15596026). DNA digestion was carried out with DNase I after RNA extraction. RNA quality was determined by measuring the A260/A280 ratio with a Nanodrop^TM^ OneC spectrophotometer (Thermo Fisher Scientific Inc.). RNA integrity was confirmed by 1.5% agarose gel electrophoresis. Qualified RNAs were finally quantified with a Qubit 3.0 fluorometer with a Qubit^TM^ RNA Broad Range Assay Kit (Life Technologies, Q10210). Total RNA (2 µg) was used for stranded RNA-seq library preparation using the KCTM Stranded mRNA Library Prep Kit for Illumina^®^ (DR08402, Wuhan Seqhealth Co., Ltd., China) following the manufacturer’s instructions. PCR products with lengths of 200-500 bp were enriched, quantified and finally sequenced on a NovaSeq 6000 sequencer (Illumina) in PE150 mode. Raw sequencing data were first filtered by Trimmomatic (version 0.36), low-quality reads were discarded, and reads contaminated with adaptor sequences were trimmed. Clean reads were mapped to the Homo sapiens reference genome using STRA software (version 2.5.3a) with default parameters. Reads mapped to the exon regions of each gene were counted by featureCounts (Subread-1.5.1; Bioconductor), and reads per kilobase per million mapped reads (RPKM)s values were then calculated. Genes differentially expressed between groups were identified using the edgeR package (version 3.12.1). A p value threshold of 0.05 and fold change threshold of 2 were used to assess the statistical significance of gene expression differences.

1. **MS**

**15.1 Protein extraction**

Frozen samples were quickly ground into a fine and uniform powder in liquid nitrogen and were then homogenized in 1 mL of phenol extraction buffer, after which 1 mL of saturated phenol with Tris-HCl (pH 7.5) was added. After several cycles of shaking, the mixture was incubated at 4 °C for 30 min. The upper phenolic phase was separated from the aqueous phase by centrifugation at 7,100 × g and 4 °C for 10 min, transferred to a fresh tube and mixed with five volumes of precooled 0.1 M ammonium acetate-methanol. After incubation at -20 °C overnight, the mixture was centrifuged at 12,000 × g for 10 min at 4 °C to pellet precipitated proteins. For the washing steps, the pellet was resuspended and washed twice with precooled methanol and twice with ice-cold acetone. Following another round of centrifugation, the pellet was collected, air-dried and resuspended in 300 µL of lysis buffer. After incubation for 3 h at room temperature, the solution was centrifuged to remove any insoluble fraction, and the resulting supernatant contained the total extractable protein. The total protein concentration was quantified by a bicinchoninic acid assay.

**15.2 Protein digestion**

According to the measured protein concentration, the same quantity of protein was taken from each sample, and the different groups of samples were diluted to the same concentration and volume. The corresponding volume of 25 mM dithiothreitol (DTT) was added to the above protein solutions to a final DTT concentration of approximately 5 mM, and the solutions were incubated at 55 °C for 30 min. Then, the corresponding volume of iodoacetamide was added to a final concentration of approximately 10 mm, and the samples were placed in the dark for 15 min at room temperature. Then, 6 volumes of precooled acetone were added to the above reaction systems to precipitate proteins, and the reaction systems were incubated at -20 °C for more than four hours or overnight. After this precipitation step, the samples were removed and centrifuged at 8,000 × g for 10 min at 4 °C to collect the precipitate. According to the amount of protein, the corresponding volume of enzymolysis diluent (protein:enzyme = 50:1 (m/m); 100 µg of protein and 2 µg of enzyme) was added to redissolve the protein precipitate, and the solutions were then incubated for digestion at 37 °C for 12 h. Finally, the samples were lyophilized or evaporated after enzymolysis.

**15.3 Enrichment of phosphorylated peptides**

Immobilized metal affinity chromatography (IMAC) was performed to enrich phosphorylated peptides. The lyophilized peptides were first suspended in 200 µL of binding/wash buffer. Next, the columns were balanced. The peptide suspensions (200 µL) were then added to the equilibrated columns and incubated for 30 min. Then, the columns were placed in a centrifuge tube and centrifuged at 1,000 × g for 30 s. The columns were then placed into a new centrifuge tube, 100 µL of elution buffer was added, and the columns were subjected to two cycles of centrifugation at 1,000 × g for 30 s each. The elution buffer was dried in a high-speed vacuum concentrator.

**15.4 LC–MS**

Proteomic data analysis was performed by Shanghai Luming Biological Technology Co., Ltd. (Shanghai, China). All analyses were performed with a timsTOF Pro mass spectrometer (Thermo, Bruker) equipped with an EASY-Spray ionization source (Thermo, USA). Nanoflow reverse-phase chromatography was performed on an EASY-nLC 1200 system (Thermo Fisher Scientific). Peptides were separated over 90 min at a flow rate of 300 nL/min on a 25 cm × 75 µm column (1.6 µm C18, IonOpticks). Mobile phases A and B were 0.1 vol% formic acid solution and 80:20:0.1 vol% ACN:water:formic acid, respectively. The total run time was 90 min (0~66 min, 3-27% B; 66~73 min, 27-46% B; 73~84 min, 46-100% B; 84~90 min, 100% B). The LC system was coupled online to a hybrid TIMS quadrupole TOF mass spectrometer (Bruker timsTOF Pro) via a CaptiveSpray nano-electrospray ion source. The capillary voltage was 1.5 kV, the dry gas temperature was 180 °C, and the dry gas flow rate was 3.0 L/minute. The dual TIMS analyzer was operated at a fixed duty cycle of approximately 100% using equal accumulation and ramp times of 100 ms each. We performed DDA in parallel accumulation–serial fragmentation (PASEF) mode with 10 PASEF scans per topN acquisition cycle. The full MS scan range was set from 100 to 1700 m/z. The ion mobility range was 0.6-1.6 vs/cm2, and the collision energy range was 20-59 eV.

**15.5 Database search**

MS/MS spectra were searched using MaxQuant against the UniProt Mus musculus database. The database-specific search parameters were set as follows: fixed modifications: carbamidomethyl (C); variable modifications: phospho (STY), oxidation (M) and acetyl (protein N-term); digestion: trypsin; first search peptide tolerance: 20 ppm; main search peptide tolerance: 10 ppm; max missed: 2.

1. **Transmission electron microscopy (TEM)**

Cells were fixed with Fixative for TEM (Servicebio, G1102) for 4 h at 4 °C, washed with phosphate-buffered saline (PBS), and postfixed with 1% OsO4 buffer for 2 h at 4 °C. The cells were then washed and dehydrated in a graded series of ethanol solutions and embedded in pure EMBed 812 overnight at 37 °C. Ultrathin (80 nm) sections were mounted on copper grids, double-stained with 2% uranyl acetate and 2.6% lead citrate solution, and examined with a Hitachi HT7800 TEM and Hitachi TEM system.

1. **WB**

Cells were collected from culture dishes and lysed using NP40 cell lysis reagent combined with proteinase and phosphatase inhibitors (the Beyotime P0027 Kit and P0033 Kit were used for the extraction of nuclear proteins and membrane proteins, respectively, from cells). The lysates were centrifuged, and the protein concentrations in the supernatants were determined using the Bradford method. The supernatants were mixed with SDS loading buffer and heated, and total protein was then separated by sodium dodecyl sulfate–polyacrylamide gel electrophoresis (SDS‒PAGE) and transferred to nitrocellulose membranes. The membranes were blocked and were then incubated overnight with primary antibodies at appropriate dilutions, as shown in **Table S2**, and then with horseradish peroxidase (HRP)-conjugated secondary antibodies. Target protein expression was evaluated using Image J software.

1. **Immunofluorescence (IF) staining and confocal microscopy**

Cells were seeded in 6-well plates containing round glass slides. 24 h later, when the cells had reached 40–50% confluence, they were washed with PBS. The cells were then fixed with 4% paraformaldehyde for 30 min and stored at 4 °C with PBS. The slides were placed in 3 changes of xylene for 10 min each, dehydrated in 3 changes of pure ethanol for 5 min each, and washed in distilled water. During the antigen repair process, excessive evaporation of buffer solution was prevented, and the slides were not dried. After antigen repair was complete, the slides were allowed to cool naturally. The slides were washed with PBS (pH 7.4) and on a decoloring shaker 3 times for 5 min each. For blocking, a circle was drawn on the tissue with a Liquid Blocker PAP Pen after the section was slightly dried, and BSA was added (binding to the primary antibody was blocked with 10% goat serum and 3% BSA from other sources) for blocking for 30 min. A mixture of the first and second primary antibodies was added. The slides were washed three times with PBS (pH 7.4) in a decoloring shaker for 5 min each. After removing the PBS, DAPI solution was dripped into the circle and incubated at room temperature for 10 min in the dark. The slides were placed in PBS (pH 7.4) and washed on a decoloring shaker 3 times for 5 min each. Autofluorescence quencher B solution was added for 5 min and was then removed by rinsing with running water for 10 min. Visualization and image acquisition were performed with a fluorescence microscope. 4’,6-Diamidino-2-phenylindole (DAPI) is visualized as blue fluorescence at a UV excitation wavelength of 330-380 nm and emission wavelength of 420 nm; Alexa Fluor 488 is visualized as green fluorescence at an excitation wavelength of 465-495 nm and emission wavelength of 515-555 nm; and CY3 is visualized as red fluorescence at an excitation wavelength of 510-560 nm and emission wavelength of 590 nm. The cells were also visualized with a Nikon Eclipse Ti (Nikon, Japan) with an Eclipse C2 system for confocal microscopy analysis. It is particularly noteworthy that in the IF staining for PD-L1, after the PD-L1 overexpression, there is actually an enhancement of signals both in the cell membrane and the nucleus. In our figure, we have selected the cells exhibiting pronounced nPD-L1 signals for display.

1. **Luciferase reporter assay**

HEK293T cells were inoculated in a 96-well plate to establish a cotransfection system consisting of 100 µL/well. The cells were transiently transfected with 0.1 μg of the firefly luciferase plasmid, 0.05 μg of the transcription factor (E2F1) or PD-L1 (CD274) overexpression plasmid, and 0.02 μg of the Renilla luciferase plasmid. In another experiment, wild-type plasmids for the RASGEF1A promoter, as well as plasmids overexpressing PD-L1 or CD44 in conjunction with the RASGEF1A promoter, were separately constructed for co-transfection, to preliminarily determine whether PD-L1 or CD44 has transcription factor activity on RASGEF1A. After 24 h to 48 h, the cell lysates were subjected to further measurement of luciferase activity with a Dual-Luciferase Reporter Assay Kit (Promega).

1. **Immunoprecipitation (IP) and Co-immunoprecipitation (Co-IP)**

Following incubation, cells were lysed in NP40 lysis buffer. The resulting products were then incubated with anti-PD-L1, anti-eIF5B and anti-MDM2 antibodies, as detailed in **Table S2**, overnight at 4 °C with rotation. The following day, the mixture was combined with Protein A/G PLUS-Agarose (Santa Cruz Biotechnology) and incubated with rotation at room temperature for 6 h. Then, the solution was centrifuged at 3,000 rpm and 4 °C and washed twice with PBS. The resulting anti-MDM2 and anti-PD-L1 antibody-conjugated Protein A/G PLUS-Agarose was finally subjected to proteomic analysis, while the PD-L1 and eIF5B immunoprecipitates were collected for subsequent analysis.

1. **Chromatin immunoprecipitation sequencing (ChIP-seq)**

ChIP-seq was conducted by Seqhealth Technology Co., LTD (Wuhan, China). The cells was fixed in 1% formaldehyde for 10 min at room temperature, after which 0.125 M glycine was added and the mixture was sat for 5 min to terminate the crosslinking reaction. The cells was treated with cell lysis buffer and nucleus was collected by centrifuging at 2,000g for 5min. Then, nucleus was treated with nucleus lysis buffer and sonicated to fragment chromatin DNA. The 10% lysis sonicated chromatin was stored and named “Input”, and 80% was used in immunoprecipitation reactions with anti-CD44 antibody and named “IP”, and 10% was incubated with rabbit IgG (Cell Signaling Technology) as a NC and named “IgG”，respectively. The DNA of input and IP was extracted by phenol-chloroform method. The high-throughput DNA sequencing libraries were prepared by using VAHTS Universal DNA Library Prep Kit for Illumina V3 (Catalog NO. ND607, Vazyme). The library products corresponding to 200-500 bps were enriched, quantified and finally sequenced on Novaseq 6000 sequencer (Illumina) with PE150 model.

1. **qPCR**

RNA was isolated from cultured cells using TRIzol (Invitrogen, Carlsbad, CA) and reverse transcribed to cDNA using the PrimeScript™ RT Kit (TaKaRa, Otsu, Japan). qPCR was performed using SYBR Premix EX Taq™ (TaKaRa, Otsu, Japan) on an FTC-3000p Real-Time PCR System (Funglyn Biotech, Shanghai, China). The comparative 2^−ΔΔCT^ method was used to determine relative gene expression levels. The PCR primers utilized are listed in **Table S2**.

1. **Assay for transposase-accessible chromatin using sequencing (ATAC-seq)**

ATAC and high-throughput sequencing and data analysis were conducted by Seqhealth Technology Co., Ltd. (Wuhan, China). A total of 10,000-50,000 cells were treated with cell lysis buffer, and nuclei were collected by centrifugation for 5 min at 500 × g. Transposase and high-throughput DNA sequencing libraries were generated using the TruePrep DNA Library Prep Kit V2 for Illumina (TD501, Vazyme). The library products were enriched, quantified and finally sequenced on a NovaSeq 6000 sequencer (Illumina) in PE150 mode. Raw sequencing data were first filtered by fastp (version 0.23.1), low-quality reads were discarded, and reads contaminated with adaptor sequences were trimmed. Clean reads were mapped to the reference genome using Bowtie2 (version 2.2.6) with default parameters. Sambamba (version 0.7.1) was used for sam/bam format conversion and PCR duplicate read removal. RSeQC (version 2.6) was used for read distribution analysis. The insert length was determined by the Collect Insert Size Metrics tool in Picard software (version 2.8.2). DeepTools (version 2.4.1) was used to visualize the distribution of reads upstream and downstream of transcription start sites (TSSs). MACS2 software (version 2.1.1) was used for peak calling. Bedtools (version 2.30.0) was used for peak annotation and peak distribution analysis. The differential peaks were identified by csaw (version 1.24.3). Homer (version 4.10) was used for motif analysis.

1. **RNA immunoprecipitation and sequencing (RIP & RIP-seq)**

RIP and high-throughput sequencing and data analysis were conducted by Seqhealth Technology Co., Ltd. (Wuhan, China). Cells were treated with cell lysis buffer. Then, 10% of the lysate was stored and named the “Input” sample, 80% was used for IP with an anti-eIF5B or anti-PD-L1 antibody and named the “IP” sample, and 10% was incubated with rabbit or mouse IgG (Cell Signaling Technology) as a NC and named the “IgG” sample. RNA was extracted from the input and IP samples using TRIzol reagent (Invitrogen, 15596026). The stranded RNA sequencing library was constructed by using the KC-DigitalTM Stranded mRNA Library Prep Kit for Illumina® (DR08502, Wuhan Seqhealth Co., Ltd., China) following the manufacturer’s instructions. The kit eliminates duplication bias in the PCR and sequencing steps by using a UMI of 8 random bases to label the preamplified cDNA molecules. The library products with lengths of 200-500 bp were enriched, quantified and finally sequenced on a NovaSeq 6000 sequencer (Illumina) in PE150 mode. Raw sequencing data were first filtered by Trimmomatic (version 0.36), low-quality reads were discarded, and reads contaminated with adaptor sequences were trimmed. Clean reads were further processed with in-house scripts to eliminate duplication bias introduced in library preparation and sequencing. In brief, clean reads were first clustered according to the UMI sequence; reads with the same UMI sequence were grouped into the same cluster. Reads in the same cluster were compared to each other by pairwise alignment, and reads with sequence identity of greater than 95% were then extracted to a new subcluster. After all subclusters were generated, multiple sequence alignment was performed to obtain one consensus sequence for each subcluster. After these steps, any errors and biases introduced by PCR amplification or sequencing were eliminated. The deduplicated consensus sequences were used for protein binding site analysis. These sequences were mapped to the reference genome using STAR software (version 2.5.3a) with default parameters. RSeQC (version 2.6) was used for read distribution analysis. exomePeak (version 3.8) software was used for peak calling. Peaks were annotated using bedtools (version 2.25.0). deepTools (version 2.4.1) was used for peak distribution analysis. The differential binding peaks were identified by a Python script using Fisher’s exact test. Sequence motifs enriched in peak regions were identified using Homer (version 4.10).

1. **MTS assay**

Cells were seeded into 96-well plates (5,000 cells per well) to facilitate cellular adhesion overnight for subsequent assays. Dilution gradients of the inhibitors were prepared and subsequently added to the culture medium for measurement of the half-maximal inhibitory concentration (IC50). After 24 h of incubation with inhibitors, MTS solution (diluted 1:9 with FBS-free RPMI-1640 medium) was added to each well, and the plate was incubated at 37 °C for an additional hour. The absorbance (A) at 490 nm was measured using an ELISA plate reader (Thermo Fisher). Additionally, we employed MTS assays to confirm cellular viability following treatment with various drug formulations, assessing the effects after 6 (cell adhesion), 24, 48, and 72 h of incubation.

1. **In vivo xenograft model**

Six-week-old female BALB/c nude mice were procured from Beijing Vital River Laboratory Animal Technology (Beijing, China). The mice were acclimated for 7 days and were then subcutaneously injected with 500,000 tumor cells to establish the subcutaneous xenograft model. The body weights of the mice were measured every 3 days. After 1 week, tumor growth at the injection site was observed daily. The tumor dimensions, including the longest diameter (a) and shortest diameter (b), were measured using a caliper. The tumor volume was calculated using the formula V = a*b^2 / 2. When the tumors reached a maximum diameter of 1 cm, the mice were divided into groups and injected (Amlodipine: intraperitoneal injection, 10 mg/kg/d) with or orally administered (Osimertinib: oral gavage, 5 mg/kg/d; Simvastatin: oral gavage, 30 mg/kg/d, the ARID1A_kd mouse model consists of 9 subjects in each treatment group, while the control group comprises 7 subjects per treatment group) the indicated drugs once daily. In addition to orally or abdominally injected drugs, this section of the study refers to the Onpattro prescription formula, with Dlin-MC3-DMA, DSPC, cholesterol, and DMG-PEG2000 as the main lipids, to prepare lipid nanoparticles (LNPs) loaded with siRNA. Dlin-MC3-DMA, DSPC, cholesterol, and DMG-PEG2000 are lipids that can be used in the human body, forming cationic lipids under acidic conditions, combining with negatively charged siRNA through electrostatic interactions to form LNPs carrying siRNA. In this experiment, microfluidic mixing was used to allow the lipid solution and siRNA solution to rapidly form uniformly sized LNPs in a micro-mixer. Since the lipids are dissolved in ethanol and the nucleic acids are dissolved in acidic buffer solution, residual ethanol was removed by ultrafiltration, and the solution system was replaced with PBS. After the preparation of LNPs was completed, the mice were treated by peritumoral injection (0.5 mg/kg/d, each group consists of five specimens). The animal experiments were carried out in strict accordance with the guidelines of the Committee on the Ethics of Animal Experiments of Cancer Hospital, Chinese Academy of Medical Sciences (No. NCC2023A316).

1. **Statistical analysis**

Nonparametric tests were utilized, and P values were determined via two-tailed tests. Statistical significance was defined as P<0.05 (*), P<0.01 (**), P<0.001 (***), or P<0.0001 (****) and determined using GraphPad Prism 9.0 software (GraphPad, La Jolla, CA, USA). Images were generated using GraphPad Prism 9.0 software (GraphPad, La Jolla, CA, USA) and Bioinfo Intelligent Cloud (BIC) [15].

**References**

1. Gao J, Aksoy BA, Dogrusoz U, Dresdner G, Gross B, Sumer SO, Sun Y, Jacobsen A, Sinha R, Larsson E, Cerami E, Sander C, Schultz N. Integrative analysis of complex cancer genomics and clinical profiles using the cBioPortal. Sci Signal. 2013, 6(269): pl1.
2. Cerami E, Gao J, Dogrusoz U, Gross BE, Sumer SO, Aksoy BA, Jacobsen A, Byrne CJ, Heuer ML, Larsson E, Antipin Y, Reva B, Goldberg AP, Sander C, Schultz N. The cBio cancer genomics portal: an open platform for exploring multidimensional cancer genomics data. Cancer Discov. 2012, 2(5): 401-404.
3. Chen X, Guo Y, Chen X. iGMDR: Integrated pharmacogenetic resource guide to cancer therapy and research. Genomics Proteomics Bioinformatics. 2020, 18(2): 150-160.
4. Tang Z, Li C, Kang B, Gao G, Li C, Zhang Z. GEPIA: a web server for cancer and normal gene expression profiling and interactive analyses. Nucleic Acids Res. 2017, 45(W1): W98-W102.
5. Shen W, Song Z, Zhong X, Huang M, Shen D, Gao P, Qian X, Wang M, He X, Wang T, Li S, Song X. Sangerbox: A comprehensive, interaction-friendly clinical bioinformatics analysis platform. iMeta. 2022, 1: e36.
6. Li T, Fu J, Zeng Z, Cohen D, Li J, Chen Q, Li B, Liu XS. TIMER2.0 for analysis of tumor-infiltrating immune cells. Nucleic Acids Res. 2020, 48(W1): W509-W514.
7. Scott MS, Troshin PV, Barton GJ. NoD: a Nucleolar localization sequence detector for eukaryotic and viral proteins. BMC Bioinformatics. 2011, 12: 317.
8. Scott MS, Boisvert FM, McDowall MD, Lamond AI, Barton GJ. Characterization and prediction of protein nucleolar localization sequences. Nucleic Acids Res. 2010, 38(21): 7388-7399.
9. Detterbeck FC, Chansky K, Groome P, Bolejack V, Crowley J, Shemanski L, Kennedy C, Krasnik M, Peake M, Rami-Porta R. The IASLC lung cancer staging project: methodology and validation used in the development of proposals for revision of the stage classification of NSCLC in the forthcoming (eighth) edition of the TNM classification of lung cancer. J Thorac Oncol. 2016, 11: 1433-1446.
10. Sun D, Wu W, Wang L, Qu J, Han Q, Wang H, Song S, Liu N, Wang Y, Hou H. Identification of MET fusions as novel therapeutic targets sensitive to MET inhibitors in lung cancer. J Transl Med. 2023, 21(1): 150.
11. Sun D, Zhu Y, Zhao H, Bian T, Li T, Liu K, Feng L, Li H, Hou H. Loss of ARID1A expression promotes lung adenocarcinoma metastasis and predicts a poor prognosis. Cell Oncol (Dordr). 2021, 44(5): 1019-1034.
12. Sun D, Feng F, Teng F, Xie T, Wang J, Xing P, Qian H, Li J. Multiomics analysis revealed the mechanisms related to the enhancement of proliferation, metastasis and EGFR-TKI resistance in EGFR-mutant LUAD with ARID1A deficiency. Cell Commun Signal. 2023, 21(1): 48.
13. Sun D, Qian H, Wang J, Xie T, Teng F, Li J, Xing P. ARID1A deficiency reverses the response to anti-PD(L)1 therapy in EGFR-mutant lung adenocarcinoma by enhancing autophagy-inhibited type I interferon production. Cell Commun Signal. 2022, 20(1): 156.
14. Li L, Sun C, Sun Y, Dong Z, Wu R, Sun X, Zhang H, Jiang W, Zhou Y, Cen X, Cai S, Xia H, Zhu Y, Guo T, Piatkevich KD. Spatially resolved proteomics via tissue expansion. Nat Commun. 2022, 13(1): 7242.
15. Chen, Tong, Yong-Xin Liu, Luqi Huang. ImageGP: An easy-to-use data visualization web server for scientific researchers. iMeta, 2022, 1: e5.
