# Supplementary material for: A cohort-based multi-omics identifies nuclear translocation of eIF5B /PD-L1/CD44 complex as the target to overcome Osimertinib resistance of ARID1A-deficient lung adenocarcinoma
Source: Exp Hematol Oncol. 2025 Jan 7;14:3. doi: 10.1186/s40164-024-00594-4 (PMC11705878; doi:10.1186/s40164-024-00594-4)
Supplement: Supplementary file 1 — Additional file 1. [file 40164_2024_594_MOESM1_ESM.zip › New folder/Table S1.docx]

**Table S1. Basic characteristics and information for patients selected in this study.**

| **Characteristics** | | **Patients number (%)** | **Correlation with the prognosis of Osimertinib treatment (P value)** |
| --- | --- | --- | --- |
|  |  |  |  |
| **Gender** | |  |  |
|  | Male | 32 (41.56%) | 0.6822 |
|  | Female | 45 (58.44%) |  |
| **EGFR mutation types** | |  |  |
|  | Exon 19 deletion | 38 (49.35%) | 0.1642 |
|  | Exon 21 L858R | 39 (50.65%) |  |
| **T790M status** | |  |  |
|  | positive | 54 (70.13%) | 0.1642 |
|  | negative | 23 (29.87%) |  |
| **Age** | |  |  |
|  | <= 60 years | 37 (48.05%) | 0.3726 |
|  | > 60 years | 40 (51.95%) |  |
| **ECOG** | |  |  |
|  | 0 | 24 (31.17%) | 0.5660 |
|  | 1-2 | 53 (68.83%) |  |
| **Treatment line of Osimertinib** | |  |  |
|  | First line | 12 (15.58%) | 0.0191 |
|  | Non-first line | 65 (84.42%) |  |
|  | **ARID1A expression** |  |  |
|  | High expression (IHC>4) | 56 (72.73%) | 0.0183 |
|  | Low expression (IHC≤4) | 21 (27.27%) |  |
| **MDM2 expression** | |  |  |
|  | High expression (IHC>6) | 38 (49.35%) | 0.0402 |
|  | Low expression (IHC≤6) | 39 (50.65%) |  |
|  | **Nuclear PD-L1** |  |  |
|  | Positive | 12 (15.58%) | 0.0035 |
|  | Negative | 65 (84.42%) |  |

**Abbreviations:**

ECOG: Eastern Cooperative Oncology Group; ARID1A: AT-rich interaction domain 1A; MDM2: murine double minute 2; PD-L1: programmed cell death-ligand 1.
