# Supplementary figures and images for: A cohort-based multi-omics identifies nuclear translocation of eIF5B /PD-L1/CD44 complex as the target to overcome Osimertinib resistance of ARID1A-deficient lung adenocarcinoma
Source: Exp Hematol Oncol. 2025 Jan 7;14:3. doi: 10.1186/s40164-024-00594-4 (PMC11705878; doi:10.1186/s40164-024-00594-4)

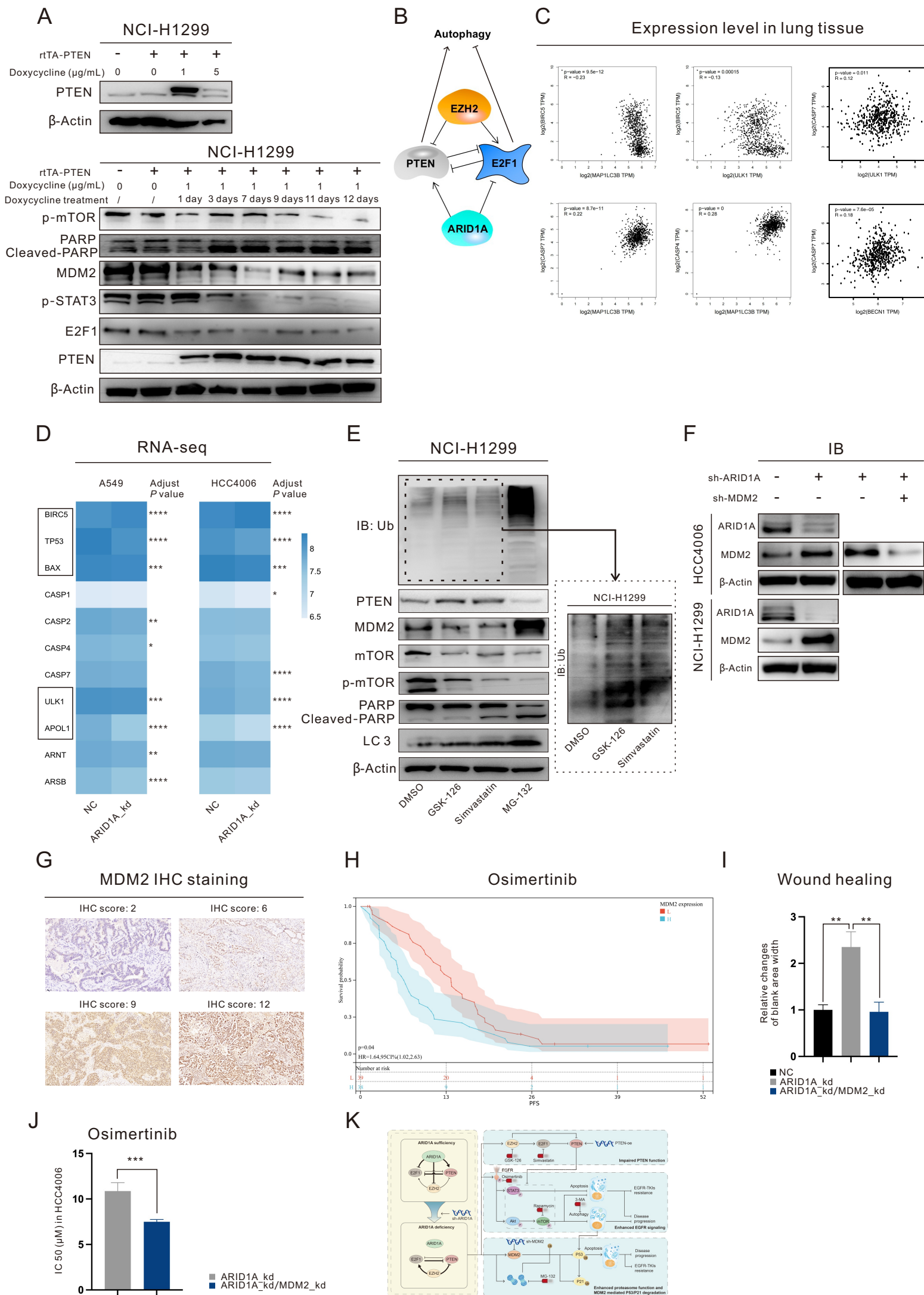

Supplement: Supplementary file 1 — Additional file 1. [file 40164_2024_594_MOESM1_ESM.zip › New folder/figure S2.pdf]

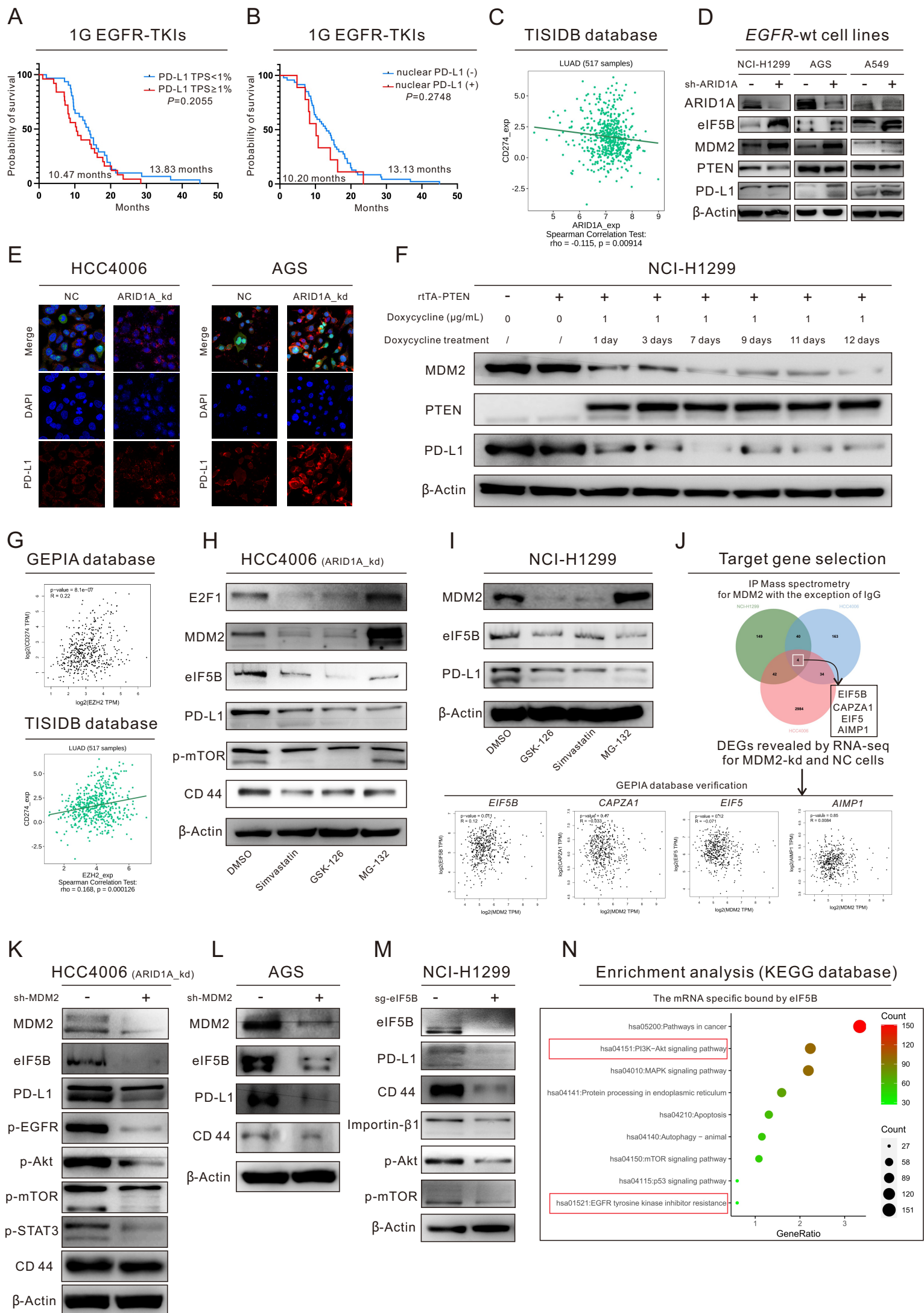

Supplement: Supplementary file 1 — Additional file 1. [file 40164_2024_594_MOESM1_ESM.zip › New folder/figure S3.pdf]

A

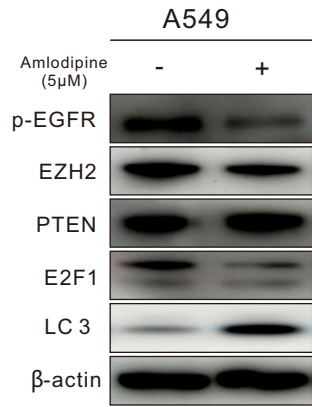

B

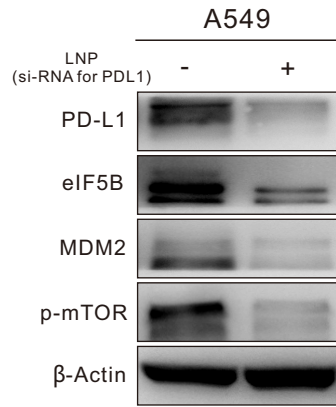

C

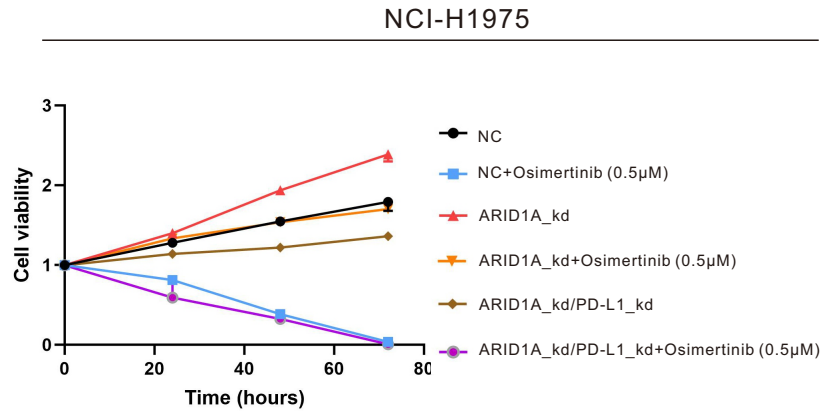

D

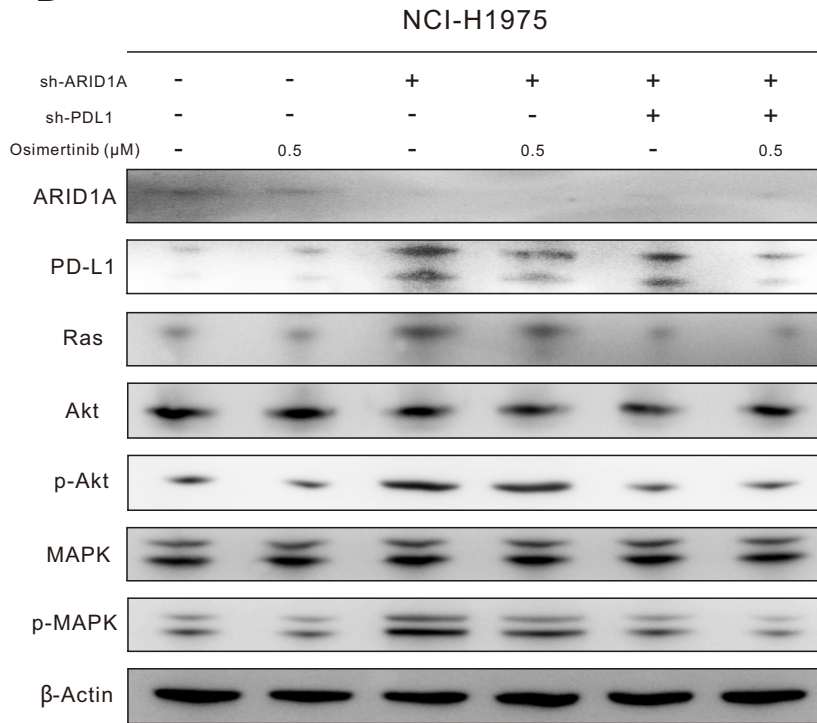

E

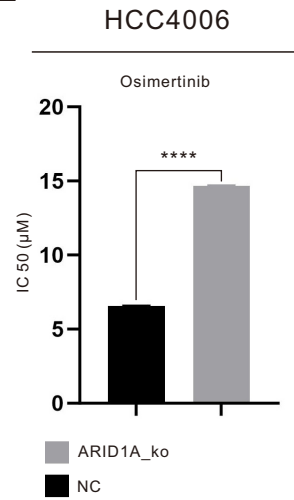

F

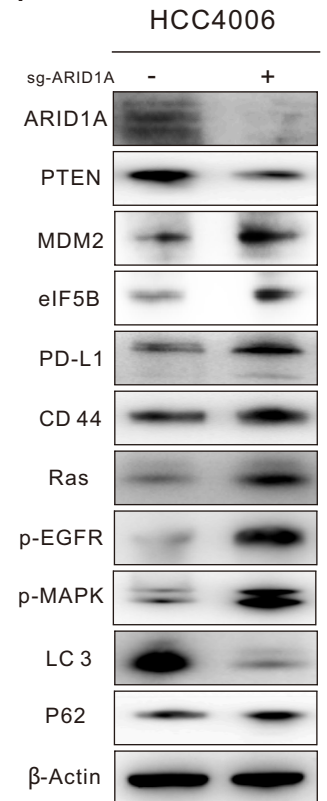

G

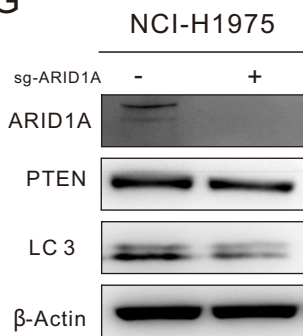

Supplement: Supplementary file 1 — Additional file 1. [file 40164_2024_594_MOESM1_ESM.zip › New folder/figure S6.pdf]
