# Supplementary material for: A cohort-based multi-omics identifies nuclear translocation of eIF5B /PD-L1/CD44 complex as the target to overcome Osimertinib resistance of ARID1A-deficient lung adenocarcinoma
Source: Exp Hematol Oncol. 2025 Jan 7;14:3. doi: 10.1186/s40164-024-00594-4 (PMC11705878; doi:10.1186/s40164-024-00594-4)
Supplement: Supplementary file 1 — Additional file 1. [file 40164_2024_594_MOESM1_ESM.zip › New folder/Table S2.doc]

**Table S2. Antibodies information, lentivirus and PCR primers sequences**

| **Antibodies and kits** | | | |
| --- | --- | --- | --- |
| **Name** | **Manufacturer** | **Number** | **Dilution rate** |
| ARID1A | Abcam | ab182560 | 1:500 (IHC); 1:1000 (WB) |
| ARID1A | Cell Signaling Technology | 12354 | 1:100 (IP/IF) |
| mTOR | Cell Signaling Technology | 2983 | 1:1000 (WB) |
| phosphorylated-mTOR | Cell Signaling Technology | 5536 | 1:1000 (WB) |
| EGFR | Cell Signaling Technology | 4267 | 1:1000 (WB) |
| phosphorylated-EGFR | Cell Signaling Technology | 3777 | 1:1000 (WB) |
| PTEN | Cell Signaling Technology | 9188 | 1:1000 (WB) |
| Beclin-1 | Cell Signaling Technology | 3495 | 1:1000 (WB) |
| P62 (SQSTM1) | Cell Signaling Technology | 88588 | 1:1000 (WB) |
| LC3 | Cell Signaling Technology | 12741 | 1:1000 (WB) |
| pan-AKT | Cell Signaling Technology | 4685 | 1:1000 (WB) |
| phosphorylated-AKT | Cell Signaling Technology | 4060 | 1:2000 (WB) |
| STAT3 | Cell Signaling Technology | 12640 | 1:1000 (WB) |
| phosphorylated-STAT3 | Cell Signaling Technology | 9145 | 1:2000 (WB) |
| E2F1 | Abcam | ab288369 | 1:1000 (WB) |
| PARP | Cell Signaling Technology | 9532 | 1:1000 (WB) |
| EZH2 | Cell Signaling Technology | 5246 | 1:1000 (WB) |
| H3K27me3 | Cell Signaling Technology | 9733 | 1:1000 (WB) |
| P53 | Cell Signaling Technology | 2524 | 1:1000 (WB) |
| β-actin | Sigma-Aldrich | A3854 | 1:5000 (WB) |
| Ubiquitin | Cell Signaling Technology | 3936 | 1:1000 (WB) |
| P21 | ZSGB-BIO | ZS-6246 | 1:1000 (WB) |
| PD-L1 | PROTEINTECH | 66248-1-Ig | 1:1000 (WB) |
| CD44 | Cell Signaling Technology | 3570 | 1:1000 (WB) |
| CD24 | PROTEINTECH | 18330-1-AP | 1:1000 (WB) |
| NANOG | PROTEINTECH | 14295-1-AP | 1:1000 (WB) |
| SOX2 | PROTEINTECH | 11064-1-AP | 1:1000 (WB) |
| Ras | Cell Signaling Technology | 3965 | 1:1000 (WB) |
| eIF5B | SANTACRUZ | sc-393564 | 1:1000 (WB) |
| Importin-β1 | Cell Signaling Technology | 51186 | 1:1000 (WB) |
| RASGEF1A | Abcam | ab229272 | 1:1000 (WB) |
| MDM2 | Cell Signaling Technology | 86934 | 1:1000 (WB) |
| α-Tubulin | Cell Signaling Technology | 2144 | 1:1000 (WB) |
| Histone H3 | Cell Signaling Technology | 9715 | 1:1000 (WB) |
| Na, K-ATPase α1 | Cell Signaling Technology | 23565 | 1:1000 (WB) |
| **Lentivirus sequences (5’-3’)** | | | |
| sh-ARID1A | GTTGATGAACTCATTGGTT | | |
| sh-PD-L1 | CACCACCAATTCCAAGAGAGA | | |
| vector control (sh-ARID1A/PD-L1) | TTCTCCGAACGTGTCACGT | | |
| sh-MDM2 | TACCTACTGATGGTGCTGTAA | | |
| vector control (sh-MDM2) | GTTCTCCGAACGTGTCACGTA | | |
| sg-eIF5B | GAATTGTCTTTGGAAGCTCA | | |
| sg-ARID1A | ATGGTCATCGGGTACCGCTG | | |
| vector control (sg-eIF5B/ARID1A) | CGCTTCCGCGGCCCGTTCAA | | |
| **PCR primers sequences** | | | |
| ARID1A-F | GAA ACC TCT GGA CCT CTA T | | |
| ARID1A-R | GTT GTT CTT GTT GAC CTG AG | | |
| Actin-F | CAT GTA CGT TGC TAT CCA GGC | | |
| Actin-R | CTC CTT AAT GTC ACG CAC GAT | | |
| CD274-F | TGG CAT TTG CTG AAC GCA TTT | | |
| CD274-R | TGC AGC CAG GTC TAA TTG TTT | | |
| **si-RNA sequence** | | | |
| si-PD-L1 | 5’-CCUAUAUGUGGUAGAGUAUTT-3’ 5’-AUACUCUACCACAUAUAGGTT-3’  CD274-2 GACAUUCAUCUUCCGUUUATT UAAACGGAAGAUGAAUGUCTT  CD274-3 CCUAUAUGUGGUAGAGUAUTT AUACUCUACCACAUAUAGGTT | | |
| NC | 5’-UUCUCCGAACGUGUCACGUTT-3’ 5’-ACGUGACACGUUCGGAGAA-3’ | | |
